# Variance estimation for effective coverage measures: A simulation study

**DOI:** 10.7189/jogh-10-010506

**Published:** 2020-06

**Authors:** Sara M Sauer, Thomas Pullum, Wenjuan Wang, Lindsay Mallick, Hannah H Leslie

**Affiliations:** 1 Department of Biostatistics, Harvard T.H. Chan School of Public Health, Boston, Massachusetts, USA; 2 The Demographic and Health Surveys (DHS) Program, Division of International Health and Development, ICF, Rockville, Maryland, USA; 3 Division of AIDS, Behavioral, and Population Sciences; Center for Scientific Review, National Institutes of Health; Bethesda, Maryland, USA*; 4 The DHS Program, Avenir Health; Glastonbury, Connecticut, USA; 5 Department of Global Health and Population, Harvard T.H. Chan School of Public Health, Boston, Massachusetts, USA

## Abstract

**Background:**

Effective coverage research is increasing rapidly in global health and development, as researchers use a range of measures and combine data sources to adjust coverage for the quality of services received. However, most estimates of effective coverage that combine data sources are reported only as point estimates, which may be due to the challenge of calculating the variance for a composite measure. In this paper, we evaluate three methods to quantify the uncertainty in the estimation of effective coverage.

**Methods:**

We conducted a simulation study to evaluate the performance of the exact, delta, and parametric bootstrap methods for constructing confidence intervals around point estimates that are calculated from combined data on coverage and quality. We assessed performance by computing the number of nominally 95% confidence intervals that contain the truth for a range of coverage and quality values and data source sample sizes. To illustrate these approaches, we applied the delta and exact methods to estimates of adjusted coverage of antenatal care (ANC) in Senegal. We used household survey data for coverage and health facility assessments for readiness to provide services.

**Results:**

With small sample sizes, when the true effective coverage value was close to the boundaries 0 or 1, the exact and parametric bootstrap methods resulted in substantial over or undercoverage and, for the exact method, a high proportion of invalid confidence intervals, while the delta method yielded modest overcoverage. The proportion of confidence intervals containing the truth in all three methods approached the intended 95% with larger sample sizes and as the true effective coverage value moved away from the 0 or 1 boundary. Confidence intervals for adjusted ANC in Senegal were largely overlapping across the delta and exact methods, although at the sub-national level, the exact method produced invalid confidence intervals for estimates near 0 or 1. We provide the code to implement these methods.

**Conclusions:**

The uncertainty around an effective coverage estimate can be characterized; this should become standard practice if effective coverage estimates are to become part of national and global health monitoring. The delta method approach outperformed the other methods in this study; we recommend its use for appropriate inference from effective coverage estimates that combine data sources, particularly when either sample size is small. When used for estimates created from facility type or regional strata, these methods require assumptions of independence that must be considered in each example.

Progress towards global development goals, including Sustainable Development Goal (SDG) 3: good health and well-being, is dependent on the operation of complex systems in both high and low-resource settings [1]. Achieving the ambitious health targets in Goal 3 demands that health systems, in order to successfully manage population health, must prevent disease, treat acute and chronic illness, and respond to emergencies and disasters [2].

While global monitoring early in the 2000s focused on health system coverage and the proportion of those in need who receive a service, there is increasing recognition that monitoring in the SDG era must evaluate the effectiveness of service in order to capture the true value of health systems [3,4]. This is reflected in the growing research on effective coverage of health interventions [5]. Effective coverage can be defined as the fraction of potential health gain successfully delivered [3], or more operationally, as the “people who need health services obtain[ing] them in a timely manner and at a level of quality necessary to obtain the desired effect and potential health gains” [6].

Effective coverage research is increasing rapidly in global health, as researchers use a range of measures to adjust coverage for the quality and effects of care received [5]. The calculation of measures of effective coverage frequently requires combining data from different sources, such as administrative records that provide service coverage and clinic data for the content or quality of services [7]. A recent review and framework for effective coverage recommended reserving the term “effective coverage” for outcome-adjusted coverage and specifying input-adjusted or process-adjusted coverage based on the type of measure used for service delivery [5]. Here we use “effective coverage” generally for any adjusted coverage estimate, given its use in existing literature, and refer to specific estimates as suggested in this framework.

As an example, multiple studies have attempted to quantify quality-adjusted coverage of antenatal care (ANC). All pregnant women are encouraged to attend formal health care during pregnancy for assessment, preventive measures, and counseling. The focused ANC model recommended by the World Health Organization (WHO) in 2002 included four visits for basic care, with the first visit taking place ideally within the first 12 weeks of gestation [8]. The Millennium Development Goal to improve maternal health included attendance at four ANC visits as a key target for service coverage [9]. In 2016, the WHO released guidelines that increased the recommended contacts to eight [10], although this has not yet been widely adopted as a measure of coverage. Studies on quality-adjusted coverage have used both one visit and four visits to quantify ANC coverage, which is based typically on maternal self-report in household surveys [11-19]. Facility measures have similarly followed WHO guidance on the expectations for ANC and have generally focused on inputs to care, with studies that define structural quality measures for health facilities such as functional diagnostics and medications [11,15,16]. Across all studies, despite the variation in measurement, crude coverage of ANC substantially exceeded the adjusted coverage. Failing to consider either facility readiness or quality of care can dramatically overstate health system performance.

Multinational and academic groups have called for a transition from coverage measures to effective coverage measures in order to appropriately benchmark and monitor national progress towards the SDGs [2,4,20]. Effective coverage metrics require accurate estimation of uncertainty around each estimate. While it is routine to report variance for effective coverage estimates that use one data source [19,21,22], most research that integrates multiple data sources to estimate effective coverage provides only point estimates. A 2018 literature review [5] found that of six papers that combined population and health facility data sources, four did not report an estimate of the variance of the point estimate [11,12,15,16]; one used the exact method for the product of two variables [17], and the sixth used a Taylor series expansion [19]. Recently published work on the effective coverage of childbirth defined and employed the delta method for variance calculation [23,24].

This paper aims to provide a formal definition of effective coverage; describe the exact, delta, and bootstrap methods of constructing confidence intervals around effective coverage estimates; test the methods’ performance with a simulation study; demonstrate their application to the estimation of effective coverage of ANC in Senegal; and provide guidance for applied research.

## METHODS

Effective coverage can be defined at three levels: *first*, for a specific combination of facility type *f* and region *r*; *second*, for region *r*, combining all facility types; *third*, at the national level, combining all regions. When combining two data sources without an exact link between individuals and health facilities, incorporating health facility type improves the accuracy of the overall estimates [25]. Here we present the analytical approaches for all three levels. We conducted the simulations at a single level to demonstrate method performance in the most simple setting.

### Calculating effective coverage

Estimates of effective coverage are specific to a particular type of intervention. For example, an intervention could be ANC for recently pregnant women age 15-49, and the relevant readiness measure could be a binary or continuous measure of facility preparedness to offer high-quality ANC. For illustration, we define a binary random variable X_rf_ that represents crude coverage and a binary random variable Y_rf_ that represents facility readiness, both of which are specific to sub-national region *r* and facility type *f.* (The full derivation of effective coverage is shown in Appendix S1 of the **Online Supplementary Document**). The distributions of these random variables are indexed by a mean parameter P(X_rf_ = 1) = P_xrf_ and P(Y_rf_ = 1) = P_yrf_, respectively. Since P_xrf_ and P_yrf_ are usually unknown in practice, these probabilities are estimated with sample means. Let *p_xrf_* be the estimated measure of coverage, such as the sample proportion of women who seek ANC in facilities of type *f* in region *r.* Let *p_yrf_* be the estimated measure of readiness, such as the sample proportion of facilities of type *f* in region *r* that satisfy minimal criteria for ANC. The estimated effective coverage *p_rf_*for this combination of *r* and *f* is a product:

*p_rf_ = p_xrf_ × p_yrf_* (1).

At the regional level, the effective coverage *p_r_* is obtained by adding the contributions from all facilities:

*p_r_* = Σ*_f_ p_rf_* (2).

This can also be viewed as a weighted sum of readiness *p_yrf_* where the weights are the proportion of women attending that facility type(*p_xrf_*, Σ*_f_ × p_rf_*  = 1). The national effective coverage *p* is a weighted average of the regional values. The weights *w_r_* (Σ*_r_ × w_r_* = 1) are proportional to the relevant denominator for each region, such as the number of women or the number of pregnancies:

*P* = Σ*_r_ w_r_ p_r_* (3).

### Calculating a confidence interval for effective coverage

We describe three methods for constructing a 95% confidence interval around an effective coverage estimate: two analytical approaches, the *exact* and the *delta* methods, and a computer-based approach, the *bootstrap* method, which involves repeated sampling with parameters estimated from the observed data. The derivation for confidence interval construction using the *delta* method was presented previously in Wang et al. [23,26].

#### Method 1: The exact method

The exact method of calculating the sampling variance of effective coverage estimates is based on a formula for the exact variance of the product of two independent random variables derived by Goodman in 1960 [27]. By definition, the variance of the product of two random variables Z and M is Var(ZM) = E [(ZM)^2^] – (E[ZM])^2^. If the two variables are independent, then E[Z^2^M^2^] = E[Z^2^] E[M^2^] and E[ZM] = E[Z] E[M], from which it follows that Var(ZM) = E[Z^2^] E [M^2^] – (E[Z] E[M])^2^. Since E[Z^2^] = Var(Z) + E[Z]^2^, and E[M^2^] = Var(M)+E[M]^2^, Var(ZM) can be re-expressed as

*Var*(*ZM*) *=* (*Var(Z)* *+* *E[Z]^2^*) *×* (*Var(M)+E[M]^2^*) *–* (*E[Z]* *E[M]*)*^2^* (4).

This formula can be used to calculate the variance of *p_rf_ = p_xrf_ × p_yrf_* denoted by *se*^2^(*p_rf_*):

*se*^2^(*p_rf_*) = [Var(*p_xrf_*) + E[*p_xrf_*]^2^] [Var(*p_yrf_*) + E[*p_yrf_*]^2^] – E[*p_rf_*]^2^ (5).

Assuming a normal approximation to the distributions of *p_xrf_* and *p_xrf_* yields Var(*p_xrf_*) = (*P_xrf_* (1–*P_xrf_*))/*n_xrf_* and Var(*p_yrf_*) = (*P_yrf_* (1–*P_yrf_*))/*n_yrf_*, where *n_xrf_* and *n_yrf_* are the sample sizes if the samples were collected via simple random sampling, or the effective sample sizes, if the samples were collected through a complex survey design. These quantities can be estimated using *p_xrf_* and *p_yrf_* in place of *P_xrf_* and *P_yrf_*, respectively. The *estimated* variance of *p_rf_* = *p_xrf_p_yrf_*, denoted by *s*^2^(*p_rf_*), is then given by:

*s*^2^(*p_rf_*) = [(*p_xrf_* (1–*p_xrf_*))/*n_xrf_* + *p^2^_xrf_*][(*p_yrf_* (1 – *p_yrf_*))/*n_yrf_* + *p^2^_yrf_*] – *p^2^_rf_* (6).

The approach under the exact method is to calculate the estimated standard error *s_rf_* = s*(p_rf_*) using (6), and then use a normal approximation to the distribution of *p_rf_* to calculate

*L_prf_*  = *p_rf_* – 1.96*s_rf_* and *U_prf_*  =  *p_rf_* + 1.96*s_rf_* (7).

as the lower and upper ends, respectively, of the 95% confidence interval for effective coverage.

To summarize, this method is exact in equations (4) and (5); equation (6) provides an estimate of the variance based on observed sample proportions; and (7) gives a symmetric Wald-type 95% confidence interval. When *p_rf_* is close to either 0 or 1, it is possible for this confidence interval to cross the 0,1 boundary and yield an invalid confidence interval.

Expression (6) can be modified to take into account the facility and regional levels as follows:

*s*^2^*_rf_* = [(*p_xrf_* (1 – *p_xrf_*))/*n_xrf_* + *p^2^_xrf_*] [(*p_yrf_* (1 – *p_yrf_*))/*n_yrf_* + *p^2^_yrf_*] – *p^2^_rf_*

*s*^2^*_r_* = Σ *_f_*[(*p_xrf_* (1 – *p_xrf_*))/*n_xrf_* + *p^2^_xrf_*] [(*p_yrf_* (1 – *p_yrf_*))/*n_yrf_* + *p^2^_yrf_*] – *p^2^_rf_*

*s*^2^ = Σ*_f_* *w*^2^*_r_* Σ*_f_* [(*p_xrf_* (1 – *p_xrf_*))/*n_xrf_* + *p^2^_xrf_*] [(*p_yrf_*  (1 – *p_yrf_*))/*n_yrf_* + *p^2^_yrf_*] – *p^2^_rf_*

Note that we assume independence (covariance = 0) between the effective coverage estimates for different facility/regional levels in the expressions above (as well as in the following methods). This is an assumption that should be evaluated in each application of these methods.

#### Method 2: The delta method

An alternative approach to constructing a 95% confidence interval for a proportion is to transform the proportion to a logit scale, calculate the confidence interval on that scale, and then take antilogits of the confidence interval endpoints to return to the original scale. Specifically, consider the proportion *P,* which is estimated by *p*. Define

*F* = *logit*(*p*) = *b* (8).

In this representation, *b* is the intercept or constant term from a logit regression with no covariates. Let *s_b_* be the standard error of *b*. Under the assumption that *b*, rather than *p*, has a normal sampling distribution, a 95% confidence interval for the population value of *b* with lower and upper limits *L_F_* and *U_F_*, respectively, is calculated as

*L_F_* = *F* – 1.96*s_b_* and *U_F_* = *F* + 1.96*s_b_* (9).

We calculate the inverse transformation of the logit, or the antilogit, to obtain the lower and upper limits of the 95% confidence interval for *P*:

L = exp(*L_F_*)/[1 + exp(*L_F_*)] and *U =* exp(*U_F_*)/[1 + exp(*U_F_*)] (10).

If *n*_0_ and *n*_1_ are the number of cases with X = 0 and X = 1, respectively, then *b* = log(*n_1_/n_0_*) and *s_b_* =sqrt[(1/*n_0_*) + (1/*n_1_*)]. Standard statistical software will produce *b*, *s_b_*, *L_F_*, and *U_F_*, and can adjust for sample weights, clustering, and stratification, if applicable.

When *p* represents the effective coverage estimate, we cannot use an exact formula to calculate *s_b_*, the standard error of *logit*(*p*), because *logit*(*p*) cannot be factored into a product of two variables. We can, however, calculate the *b*’s and *s*’s from the readiness and coverage data separately, and use these to approximate *s_b_*. Suppose that *b*_x_ and *b*_y_ are the intercepts from logit regressions of *X* and *Y* separately, with sampling *s^2^_bx_* and *s^2^_by_*, respectively, all of which are produced with standard statistical packages. If *F* = *F*(*b_x_*,*b_y_*) is a joint function of *b*_x_ and *b*_y_, then an approximation to the sampling variance of *F* is given by


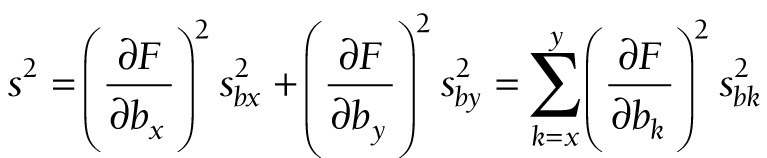
 (11).

This well-known approximation is sometimes described as the delta method [28].

Now we apply the delta method to the logit of effective coverage. The logit transformation is applied to three levels of effective coverage: *first*, for facility type *f* in region *r*; *second*, for region *r*; and *third*, at the national level, for which no subscripts are needed. The transformations are

*F_rf_* = *logit*(*p_rf_*) (12).

*F_r_* = *logit*(*p_r_*) (13).

*F* = *logit*(*p*) (14).

At these three levels, (11) takes the following forms:


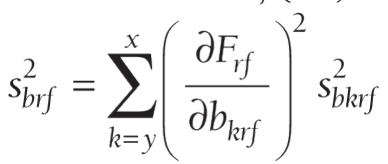
 (15).


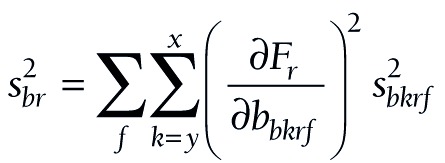
 (16).


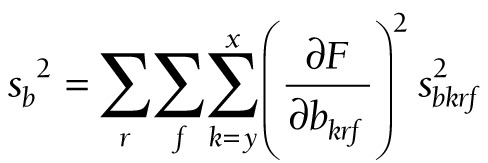
 (17).

The components for the chained partial derivatives are provided in Appendix S2 of the **Online Supplementary Document**. When the components are multiplied together as described, the partial derivatives required for equations (15), (16), and (17) are given by (18), (19), and (20), respectively:


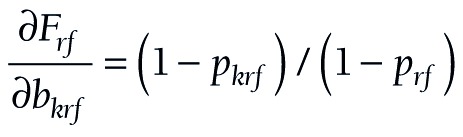
 (18).


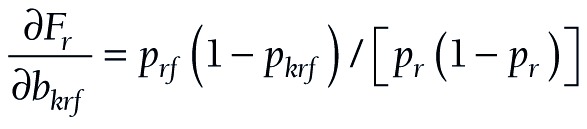
 (19).


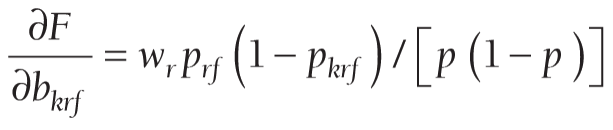
 (20).

To summarize this approach, partial derivatives are calculated from (18) or (19) or (20). The s’s are produced by logit regression software. At each level, a confidence interval is constructed for *F*, and the endpoints of that interval are converted to a confidence interval (*L*, *U*) for *P* by taking antilogits, as in (10).

**Figure 1:**
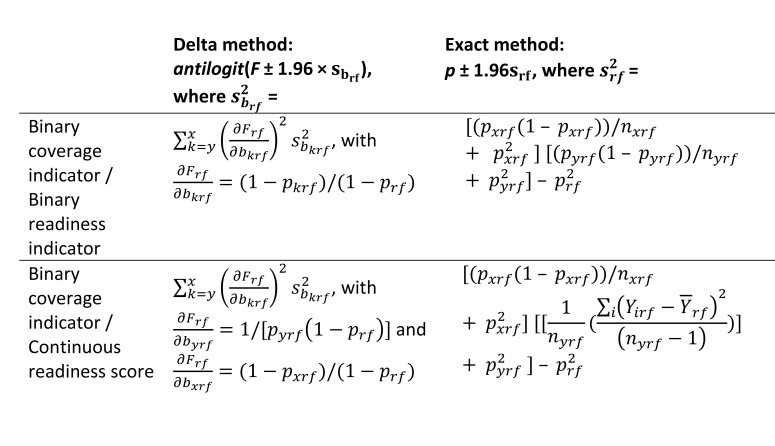
Delta and exact methods for construction of a 95% confidence interval around the effective coverage estimate for a specific facility type *f* in a specific region *r, p_rf_*.

In some cases, facility readiness may be a continuous score rather than a binary measure. **Figure 1** summarizes the variance calculations for the exact and delta methods in the case of a binary coverage indicator and a binary or a continuous readiness measure. Details of the modifications to the formulas in the case of a continuous readiness measure are shown in Appendix S3 of the **Online Supplementary Document**.

#### Method 3: The bootstrap method

The final approach we consider is a bootstrap method, a computer-based approach that involves repeated sampling from a distribution or the observed data [29]. Bootstrapping methods are used increasingly to generate variance estimates for complex parameters. We first compared a non-parametric bootstrap in which observations are re-sampled from observed data (with replacement) to a parametric bootstrap in which observations are generated with parameters estimated from the original sample. Initial comparisons revealed consistent undercoverage when using a nonparametric bootstrap. We therefore focus on the parametric bootstrap for the full analysis. When coverage and readiness are proportions as presented above, the steps for constructing a 95% confidence interval for the effective coverage estimate for a particular region *r* and facility type *f* based on a simple random sample for each data source are as follows:

- Obtain *p_xrf_* and *p_yrf_* from the observed data,
- Use these estimates to generate B samples from Binomial(*n_xrf_, p_xrf_*), and B samples from Binomial(*n_yrf_, p_yrf_*),
- Divide the sampled values by *n_xrf_*, and *n_yrf_* respectively to get the B-vectors *p_xrf_boot_* and *p_yrf _boot_*,
- Multiply the corresponding elements of *p_xrf_boot_* and *p_yrf _boot_* to get the B-vector *p_boot_*,
- Obtain the upper and lower limits of the 95% confidence interval using the 2.5th and 97.5th percentiles of the distribution of *p_boot_*,

If readiness is a continuous score, this procedure can be modified to generate data using an appropriate distribution.

### Simulation study

We designed a simulation study to test the performance of the confidence interval construction methods in settings with small and modest sample sizes. In many cases in applied research, the sample size for health facilities will be much smaller than the individual sample. We tested settings of both equal and unequal sample sizes.

We assume that the crude coverage of a service in the population is a binary measure with probability P_xrf_ and health system quality is a binary measure with probability P_yrf_. We test combinations of P_xrf_ and P_yrf_ over the range (0.02, 0.98) and with sample sizes for *n_xrf_*, the number of individuals sampled, and *n_yrf_*, the number of facilities sampled, that varied between 50 and 500 (all settings considered are presented in **Table 1**). In each case, the sample of individuals and the sample of facilities are simple random samples. As a further analysis, we consider the case in which the service readiness measure is a continuous score on 0,1.

**Table 1:** Simulation settings

Term	Definition	Simulation settings
P_xrf_	Proportion of individuals in need accessing service (crude coverage)	(0.02, 0.04, 0.06, 0.08, 0.1, 0.15, 0.2, 0.25, 0.30, 0.35, 0.40, 0.45, 0.50, 0.55, 0.6, 0.65, 0.70, 0.75, 0.80, 0.85, 0.90, 0.92, 0.94, 0.96, 0.98)
P_yrf_	Proportion of health facilities rated as ‘high-quality’	(0.02, 0.04, 0.06, 0.08, 0.1, 0.15, 0.2, 0.25, 0.30, 0.35, 0.40, 0.45, 0.50, 0.55, 0.6, 0.65, 0.70, 0.75, 0.80, 0.85, 0.90, 0.92, 0.94, 0.96, 0.98)
n_xrf_	Number of individuals sampled	(n_xrf_, n_yrf_) = (50, 50), (100, 100), (200, 200), (300, 300), (400, 400), (500, 500), (100, 50), (200, 50), (300, 50), (400, 50), (500, 50)
n_yrf_	Number of facilities sampled	

For each simulation setting, we generated 10 000 data sets. For each simulated data set, we computed the effective coverage estimate *p_rf_ = p_xrf_p_yrf_* and constructed 95% confidence intervals around *p_rf_* using the exact, delta, and bootstrap methods. For the bootstrap method, 10 000 bootstrap samples were taken to construct the confidence interval at each iteration. Across the 10 000 simulated iterations, we calculated the estimated coverage probabilities of the three methods for constructing a 95% confidence interval by computing the proportion of iterations in which the true effective coverage value was contained in the 95% confidence interval. When computing the estimated coverage probabilities for the exact and bootstrap methods, we removed the iterations that resulted in a degenerate confidence interval, which occurs when *p_rf_* = 0 or 1. When estimating the coverage probabilities for the delta method, we removed the iterations that resulted in an undefined confidence interval, which occurs when when *p_rf_* = 0 or when either *p_xrf_* or *p_yrf_*  = 1. We recorded the number of undefined confidence intervals for the delta method, which is the maximum number of iterations removed across the three methods, and the number of invalid estimates for the exact method (the instances in which the confidence interval bounds fall below 0 or above 1).

### Applied example

We demonstrate the application of these methods using real data on ANC coverage and facility readiness in the Republic of Senegal. The West African nation of Senegal is home to over 13 million individuals who live in 14 administrative regions [30]. Senegal is classified as a low-income country by the World Bank. The public health sector is organized into hospitals at the top, followed by intermediate health centers and peripheral health posts. Health extension huts (*cases de santé*) offer limited services, which are largely for reproductive, maternal, and child health, while the private sector also provides health services. From 2012 to 2017, the National Agency for Statistics and Demography partnered with ICF International to implement a simultaneous and continuous Demographic and Health Survey (DHS) and Service Provision Assessment (SPA). Each assessment was administered annually over the 5-year period. We relied on the 2017 surveys, using the DHS to define coverage and the SPA to define readiness.

The DHS is a population-based household sample, in which women age 15 to 49 answer questions about their reproductive history. We defined ANC coverage based on responses from women with a live birth in the past 2 years, and calculated three measures based on global and national standards of care:

- Coverage 1: self-report of any ANC with a formal health care provider
- Coverage 4: self-report of 4 or more ANC visits
- Coverage 8: self-report of 8 or more ANC visits

The SPA is a health facility assessment that includes an audit of service availability and readiness, as well as provider interviews and observations of care. We use the facility audit to define readiness to provide high-quality ANC, which was limited to health facilities that provide ANC. To best illustrate the variance estimation methods, we defined two binary indicators (one prevalent, one rare) and a bounded score as quality measures:

- Readiness 1: Availability of manual or digital blood pressure apparatus (binary)
- Readiness 2: Availability of hemoglobin and urine protein diagnostic capacity (binary)
- Readiness 3: ANC service readiness, the proportion of essential inputs in place across four domains as defined by the WHO: basic amenities (guidelines, visual aids, and provider training); equipment (manual or digital blood pressure apparatus); diagnostics (urine and anemia testing capacity); and medication (iron and folic acid, tetanus toxoid, and IPTp and ITNs for malaria) [31]. We weighted items evenly within domain and domains equally within the score.

To define adjusted coverage, we calculated service readiness by strata defined by the fourteen regions and five health facility types (government hospital, government health center, government health clinic/post, private hospital/health center/clinic, health hut). Coverage was estimated for the same strata. In this example, we assigned women who identified multiple sources of care the source with the highest readiness and assumed that all visits took place at that source or a similar source. This will overestimate adjusted coverage for women using multiple sources of care of varying readiness. We calculated adjusted coverage as the product of coverage and readiness within strata and estimated national adjusted coverage as the average of stratum-specific estimates weighted by population size (women reporting live births in the previous 2 years).

We repeated this procedure for the nine combinations of the coverage and quality measures and used the exact and delta methods to quantify variance in each case. The complex sample designs used for both DHS and SPA are accounted for by using the survey setting. For the DHS, the strata are based on sub-national region and urban/rural residence; the cluster is the primary sampling unit, and individual weights are the women’s sampling weight that account for non-response and sampling probability. For the SPA, the strata are based on sub-national region, health facility type, and managing authority; the primary sampling unit is the facility, and the individual weights are the facility sampling weight. Although the bootstrap method, particularly the non-parametric bootstrap, can be modified for complex survey data [32], expanding the parametric bootstrap method for a parameter derived from two complex surveys is beyond the scope of this paper and is an area for future research.

Simulations were conducted in R (The R Foundation for Statistical Computing, Vienna, Austria). Applied analyses were conducted with Stata version 15 (StataCorp, College Station, Texas, USA). A program for calculating point estimates and confidence intervals in Stata for national and sub-national analyses like the applied example as well as sub-sample analyses to assess equity is included in the **Online Supplementary Document** and at this link (https://osf.io/9nsaf/?view_only=681d595548014a17a4a666690e708336), as is the simulation code in R.

### Ethical approval

The original survey implementers obtained ethical approvals for data collection; the Harvard University Research Protection Program deemed this analysis exempt from human subjects review.

## RESULTS

### Simulation study

**Figure 2:**
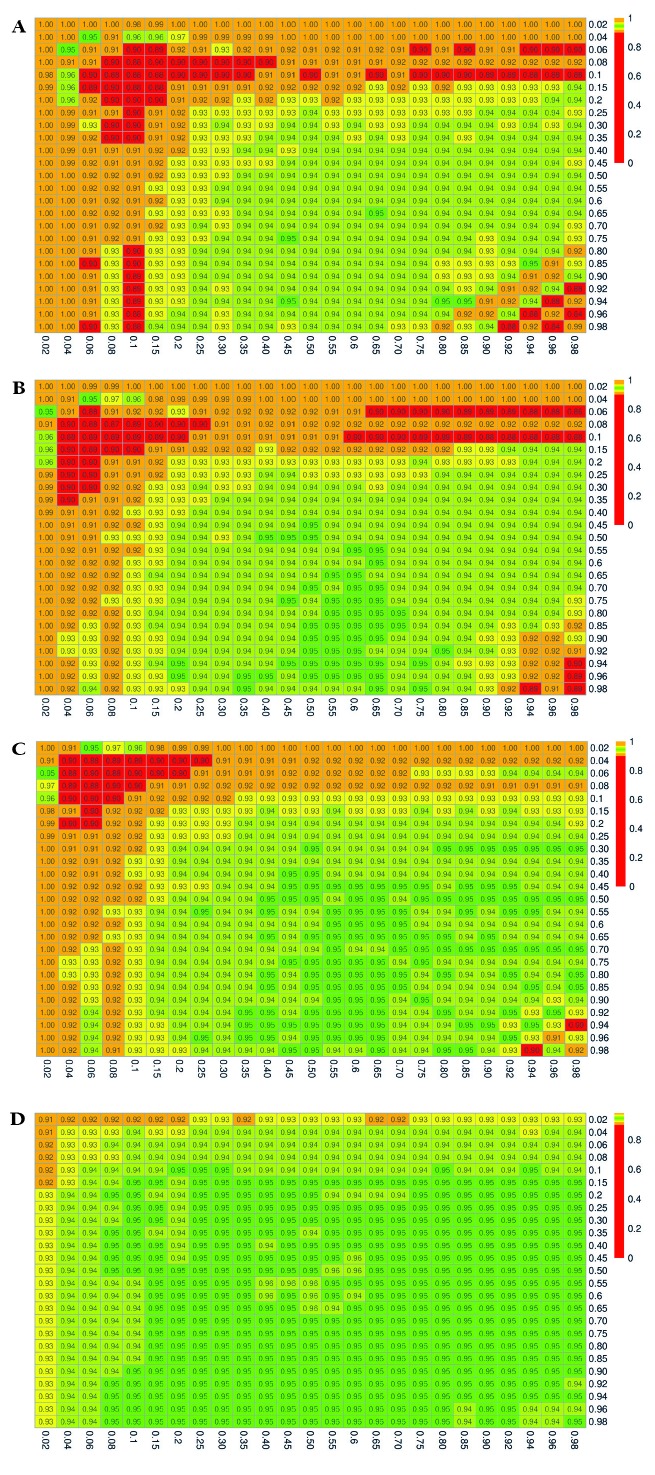
Estimated 95% coverage probabilities for the confidence intervals constructed using the exact method. P_xrf_ and P_yrf_ vary over the range (0.02, 0.98). **Panel A.** (n_xrf_, n_yrf_) = (50, 50). **Panel B.** (n_xrf_, n_yrf_) = (100, 50). **Panel C.** (n_xrf_, n_yrf_) = (100, 100). **Panel D.** (n_xrf_, n_yrf_) = (500, 500).

**Figure 3:**
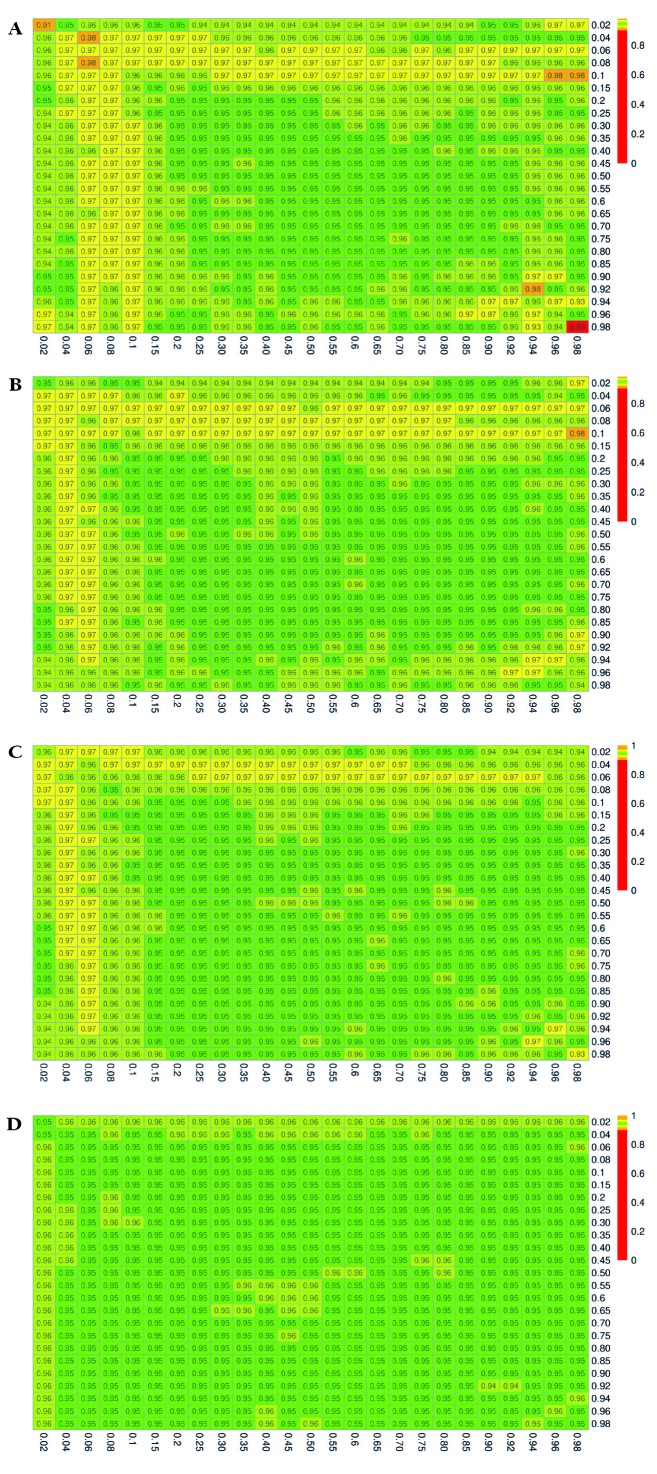
Estimated 95% coverage probabilities for the confidence intervals constructed using the delta method. P_xrf_ and P_yrf_ vary over the range (0.02, 0.98). **Panel A.** (n_xrf_, n_yrf_) = (50, 50). **Panel B.** (n_xrf_, n_yrf_) = (100, 50). **Panel C.** (n_xrf_, n_yrf_) = (100, 100). **Panel D.** (n_xrf_, n_yrf_) = (500, 500).

**Figure 4:**
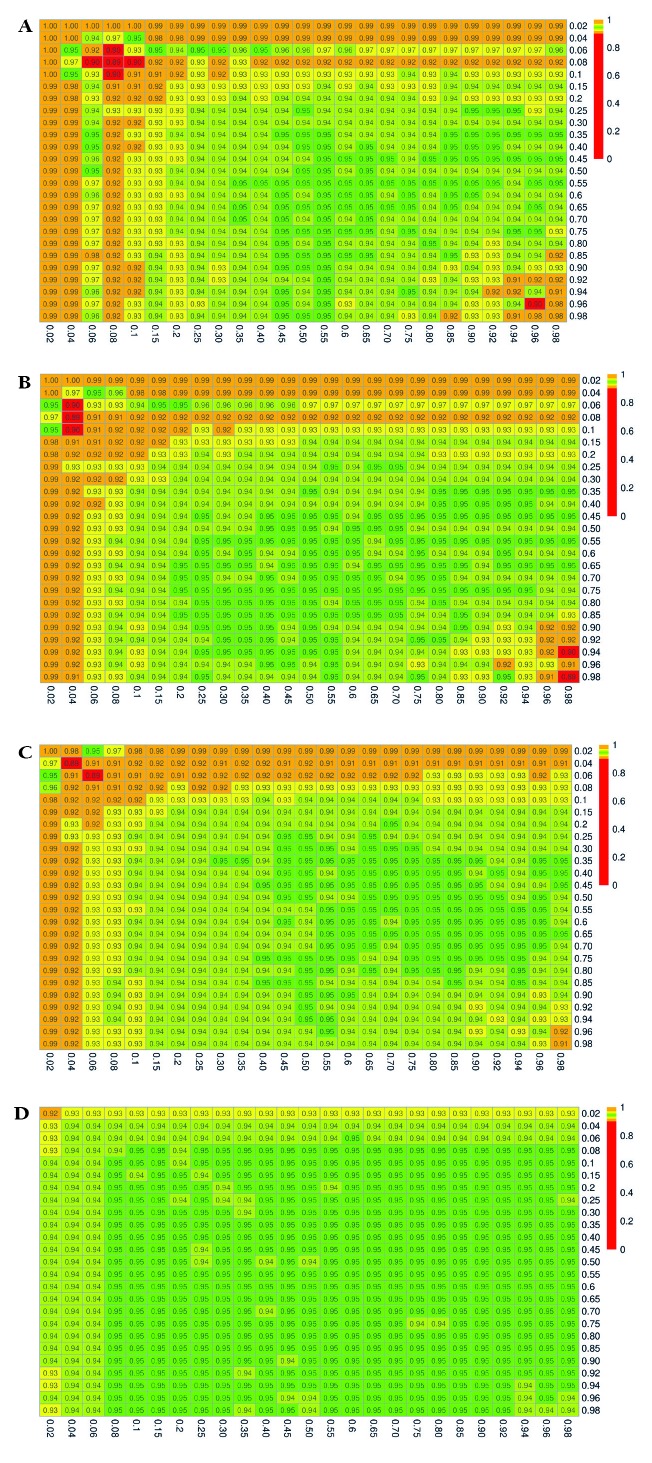
Estimated 95% coverage probabilities for the confidence intervals constructed using the parametric bootstrap method. P_xrf_ and P_yrf_ vary over the range (0.02, 0.98). **Panel A.** (n_xrf_, n_yrf_) = (50, 50). **Panel B.** (n_xrf_, n_yrf_) = (100, 50). **Panel C.** (n_xrf_, n_yrf_) = (100, 100). **Panel D.** (n_xrf_, n_yrf_) = (500, 500).

The simulation results for the estimated coverage probabilities are shown in **Figure 2**, **Figure 3**, and **Figure 4** for the exact, delta, and bootstrap methods, respectively. The maximum proportion of simulations with undefined estimates is shown in Appendix S4 in the **Online Supplementary Document**.

When the sample size is small, and when the true proportions *P_xrf_* and/or *P_yrf_* are close to 0 or 1, the confidence intervals constructed using the exact method result in severe overcoverage for the most extreme values of *P_xrf_* and *P_yrf_*, which then becomes undercoverage as *P_xrf_* and *P_yrf_* increase. Of the iterations that do not result in degenerate confidence intervals, the nominally 95% confidence interval captures the truth 100% of the time in the extreme case of *P_xrf_*  =  0.02 and *P_yrf_*  = 0.02 with (*n_xrf_*, *n_yrf_*) = (50, 50), as shown in **Figure 2**, Panel A. Moreover, as shown in Appendix S4 of the **Online Supplementary Document**, all of the non-degenerate confidence intervals constructed in this setting are invalid, with the lower bound falling below 0. For small sample sizes and values of *P_xrf_* and *P_yrf_* between 0.06 and 0.25, the exact method yielded confidence intervals that contained the truth closer to 90 than 95% of the time. This undercoverage may be due to the use of a normal approximation, which may not be valid with small sample sizes. A similar pattern persists at sample sizes of 100 (**Figure 2**, Panels B and C). When both *n_xrf_* and *n_yrf_* are 500, the coverage is generally at or close to the nominal level (0.95), except for very extreme values of *P_xrf_* or *P_yrf_* (**Figure 2**, Panel D).

The confidence intervals constructed with the delta method yield estimated coverage probabilities that are generally close to the nominal level of 95%, although for small sample sizes and extreme values of *P_xrf_* or *P_yrf_*, this method results in overcoverage as high as 98% (**Figure 3**, Panel A). Coverage is uniformly close to the nominal 95% level once sample sizes reach 400 to 500.

The results for the bootstrap method mirror those described for the exact method, with slightly better performance compared to the exact method. In particular, we see overcoverage (99-100%) for small sample sizes and very extreme values of *P_xrf_* or *P_yrf_*, which then becomes undercoverage for values of *P_xrf_* and *P_yrf_* up to approximately 0.25 (**Figure 4**, Panel A). With samples of at least 500, coverage is no lower than 92% (**Figure 4**, Panel D).

Appendix S4 in the **Online Supplementary Document** includes full results with the number of invalid confidence interval bounds for the exact method, which occurred in as high as 100% of the iterations yielding non-degnerate confidence intervals for effective coverage estimates near the boundary, when *n_xrf_* = *n_yrf_* = 50, and occurred as much as 26% of the time when *n_xrf_* = *n_yrf_* = 500.

When using a continuous score as a readiness estimate instead of a binary indicator, the relative performance of the confidence interval construction methods was comparable, with the delta method demonstrating 95% coverage plus or minus only a few percent in nearly all settings (Appendix S5 in the **Online Supplementary Document****,** **Figure 3**). The results for the exact and bootstrap method, were similar to the results in the setting with a binary readiness estimate, except that the over- and undercoverage arises in settings with small sample sizes and extreme values of *P_xrf_* (binary coverage) only. The exact method and to a lesser extent the bootstrap method resulted in coverage below the nominal 95% level in small samples when *P_xrf_* true coverage was less than 10% at any level of readiness or when true coverage was greater than 0.9 and readiness exceeded 0.5 (Appendix S5 in the **Online Supplementary Document**, **Figure 2**, Panel A, **Figure 4****,** Panel A). Coverage probabilities were all close to 95% once both sample sizes reached 500.

### Applied example

**Figure 5:**
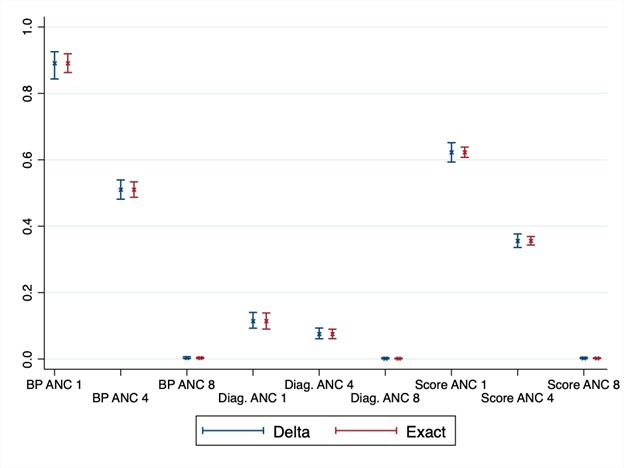
Adjusted coverage estimates of antenatal care in Senegal with 95% confidence intervals using the delta and exact methods. Coverage is defined as 1, 4, or 8 antenatal care visits (ANC 1, ANC 4, ANC 8 respectively). Readiness is defined based on functional blood pressure apparatus (BP), functional diagnostics (Diag.), or summary score (Score).

**Figure 5** shows the adjusted coverage estimates and 95% confidence intervals constructed with the exact and delta methods for the nine combinations of coverage and quality measures, as described in the Methods section. Adjusted coverage of ANC in Senegal differed as expected, based on the definitions of coverage and readiness, from a high of 89% using a single ANC visit and facility readiness, such as blood pressure apparatus, to a low of less than 1% for all estimates based on receiving eight or more ANC visits. Confidence intervals are wider with the delta method, although for most estimates, both methods provide largely overlapping intervals. At the national level, all intervals were within the bounds of 0 to 1, although for the region-specific estimates (Appendix S6 in the **Online Supplementary Document**) with smaller sample size, the exact method produced 33 (out of 126) invalid confidence intervals that crossed the 0, 1 bounds. These violations affected the estimates near the boundaries: ANC 1 and blood pressure apparatus on the high end, and all estimates based on ANC 8 and/or appropriate hemoglobin and urine protein diagnostic capacity on the low end. The delta method could not be applied in the 24 cases where the adjusted coverage estimate was 0 for a particular type of coverage and a given sub-national region. While the exact method confidence intervals are symmetrical, using the logit transformation in the delta method produces asymmetrical intervals to better reflect the bounds of a proportion. In the case of regions with small sample sizes and adjusted coverage estimates near 1, the lower bound could be quite large, for instance adjusted coverage of 1 ANC visit using blood pressure apparatus as the readiness measure is 97.8%, 95% CI = 15.3%-100%.

**Figure 6:**
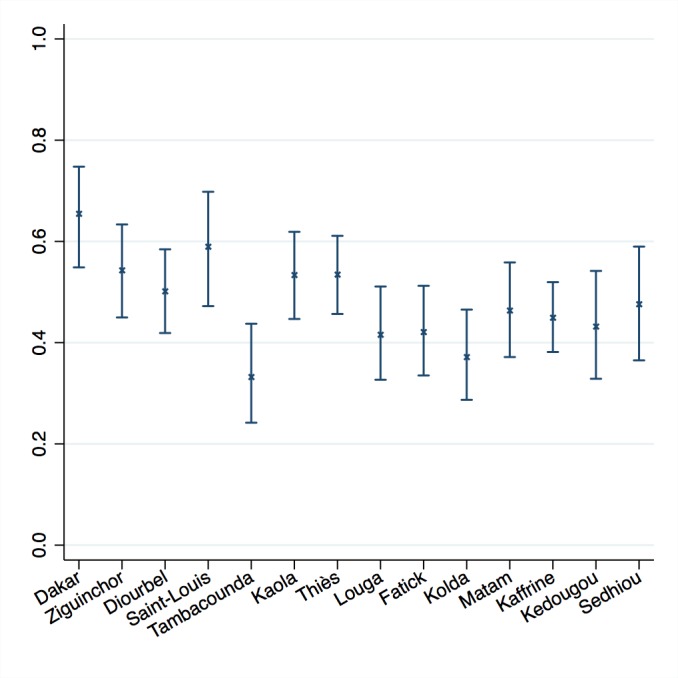
Adjusted coverage of antenatal care in 14 regions of Senegal with 95% confidence intervals. Results shown for coverage defined as at least 4 antenatal care visits, readiness defined as functional blood pressure apparatus.

Calculating confidence and variance in these adjusted coverage estimates enables inference to be drawn on the results. **Figure 6** shows the delta method results for service readiness based on blood pressure apparatus for four ANC visits. Depicting the variance around the point estimates allows us to identify significant differences between regions and to target the underperforming regions.

In this example, the assumption of independence between regions is likely met as the sampling strategy for both surveys is stratified by region. The assumption of independence between adjusted coverage for facility types within a region is likely not met, because although the SPA facility survey is stratified by facility type (and managing authority), the DHS household survey is not and could not be. However, the covariance between the facility-specific coverage estimates within a region is negative (see Appendix S7 in the **Online Supplementary Document**), which makes the covariance between the facility-specific *effective coverage* estimates within region negative as well. Ignoring this covariance should result in an overestimate of variance and a conservative confidence interval.

## DISCUSSION

Effective coverage is the product of two quantities with their own sampling error, and as such, is an atypical parameter for variance estimation. In this paper, we evaluated the performance of the exact, delta, and bootstrap methods for constructing confidence intervals around effective coverage estimates. The delta method approach performed better than the other methods: it yielded close to nominal coverage, ie, that 95% confidence intervals captured the truth in 95% of the simulations for a given setting, in nearly all cases. The exact and parametric bootstrap methods resulted in undercoverage of nominally 95% confidence intervals – leading to Type I errors in inference – in settings with small sample sizes or coverage and quality measures close to 0 or 1. The exact method also produced invalid confidence intervals in such settings. The non-parametric bootstrap demonstrated consistent undercoverage and was not analyzed in detail.

We applied the delta and exact methods to calculate adjusted coverage of antenatal care in Senegal, using a sample of women with recent live births from the DHS household survey and a sample of health facilities from the SPA facility audit. Both data sources provide estimates with their own sampling error. Using the delta and exact methods enabled us to incorporate both sources of variance. The results demonstrate that the delta and exact methods can be applied to obtain confidence intervals for complex survey data at sub-national and national levels to support inference around levels and trends in adjusted coverage measures. The delta method was more robust in providing valid results even for small sub-national regions; researchers should be aware that confidence intervals may be quite wide for adjusted coverage estimates near 1.

This work shows that it is feasible to characterize the uncertainty around an effective coverage estimate calculated from separate data sources. Variance estimation should become standard practice if effective coverage estimates are to become part of national and global health monitoring [2,5,33]. The methods outlined here may further be applicable to effective coverage estimation in educational settings, such as the human capacity index developed by the World Bank to capture years of quality-adjusted education as a composite measure of years of schooling and achievement test scores [34,35]. Accurate estimation of variability enables stronger inferences that can inform comparisons and targeted improvement actions. Of the methods considered here, we recommend using the delta method approach – because of its superior performance compared to the exact and bootstrap methods, and since the bootstrap approach outlined in this paper has not yet been adapted for effective coverage estimates based on complex survey designs. In order to facilitate use of these methods, we provide code that can be used by researchers for constructing confidence intervals in a variety of settings, including with complex survey data such as the DHS and SPA.

One limitation of this work is that the simulation studies considered the setting in which the quantities used for effective coverage estimation are computed using simple random samples from the data sources; we note that while we expect the relative performance of the methods to hold for quantities estimated using data from a complex sample, the precise coverage levels may differ to a small extent. A second limitation is that to simplify the variance expressions for the exact and delta methods, any covariance between the facility-level or regional-level effective coverage estimates is ignored. In other words, the facility/region-specific effective coverage estimates are taken to be independent in the derivation of the variance formulas. The assumption is not required for cases with a single geographic area and single facility type (or homogeneous quality measures across observed facility types). In the applied example here, where this covariance is nonzero due to dependence of adjusted coverage estimates across different facility types within each region, it is negative and would reduce the variance if accounted for. Investigating the magnitude of this covariance and modifying the methods to take it into account for increased precision are areas for future research.

Researchers should assess the assumptions of independence between region-specific estimates or facility-specific estimates within a region based on the sampling design of the data sources used and note this limitation as relevant. For example, for estimates based on SPA and DHS data, the assumptions for the current analysis would be the same if we further sub-divided facility types by managing authority [30]. However, the assumptions would not hold if adjusted coverage estimates were computed at a geographic level below that used in the sampling schemes for SPA and DHS.

Defining and quantifying variance for effective coverage estimates underscores the atypical elements of this parameter as a product of two proportions or bounded scales. We identified several areas for further investigation on technical elements of the variance. These include modest overcoverage of delta method confidence intervals in the simulation studies with extreme values of coverage and readiness; the assumption that the sampling distribution of F (formula 14, the logit of effective coverage) is approximately normal may not be met in such extreme cases. Further, the poor performance of the non-parametric bootstrap and the lack of parametric bootstrap estimator available for a product of estimates from complex survey data are gaps that warrant development or refinement of bootstrap methods for this setting.

The methods defined here presume that quantities such as facility readiness are measured without sampling error. Further work is required to adapt the confidence interval construction methods to incorporate error in the estimates of coverage or quality. For instance, estimates of quality for adjusted coverage may be based on a sample of health workers [18], observed patient visits [17], or clinical records [36,37]. Researchers must determine if these within-facility measures provide consistent estimates of readiness at the facility level and, if so, how to capture the within-facility sampling error in composite effective coverage.

While incorporating variance in the sampled estimates of coverage and health system quality is important in accurately reporting effective coverage, the methods described here do not incorporate sources of variability that are not captured in the source data. For example, applications of effective coverage estimation frequently combine population data covering a number of years (ANC for live births in the past 2 years would cover visits for pregnancies beginning up to 33 months before the survey) with health system estimates from a single point in time. A particular strength of the data from Senegal used here is that these surveys were conducted simultaneously, but this is more the exception than the rule in existing population and health system surveys. Confidence interval calculation will not address variance introduced from changes in health system measures over time. These sources of uncertainty should be stated in reporting effective coverage results or incorporated via quantitative bias analysis.

Because measures of crude coverage may obscure the deficits of the health system, assessing effective or adjusted coverage is important in order to recognize both the extent of contact with services, as well as the quality of the services received. Uncertainty estimates that account for the variability in the coverage and quality estimates provide more accurate information for understanding the potential range of quality-adjusted coverage and enabling comparison of these estimates over time and between regions, or countries. Yet, until now, estimates of variance have been largely absent from analysis of effective coverage that combined multiple data sources. This study fills this gap by exploring and comparing three methods for calculating variance. We recommend the delta method as the primary approach for variance calculation, provide Stata code for confidence interval calculation, and outline the assumptions researchers should assess in its application to benefit future research.

## Additional material

Online Supplementary Document
