# Erratum: Variance estimation for effective coverage measures: A simulation study

**DOI:** 10.7189/jogh.11.01009

**Published:** 2021-11-15

**Authors:** Hannah Leslie

**Affiliations:** Division of Prevention Science, Department of Medicine, University of California San Francisco, San Francisco, California, USA

I write with a correction to the article, “Variance estimation for effective coverage measures: A simulation study,” published in the June 2020 issue of the *Journal of Global Health* [1].

In the provided code to estimate effective coverage calculations, the sub-routine “extract scalars” (lines 373-36) did not correctly handle the option for continuous quality scores, resulting in continuous scores being treated as binary for one part of the calculation. This error affected the levels of adjusted coverage estimated using a continuous quality score in our applied example: the national adjusted coverage of ANC using a summary score of readiness should be 64.1% (ANC 1), 36.7% (ANC 4) and 0.25% (ANC 8) instead of 62.3%, 35.6%, and 0.24% as we reported. Sub-national estimates provided in the supplemental information differed by 1.5 percentage points on average. The attached **Online Supplementary Document** includes the corrected Figure 5: Adjusted coverage estimates of antenatal care in Senegal with 95% confidence intervals using the delta and exact methods, Appendix S6: Sub-national results, and the program for effective coverage calculation. The code is also available at https://osf.io/9nsaf/.

The corrections to the level of adjusted coverage do not affect the findings of the article or implications regarding appropriate calculation of confidence intervals for effective coverage calculations. I regret not identifying this error before publication and appreciate *Journal*’s efforts to provide the corrected materials to other researchers.

## Additional material


Online Supplementary Document
