# Supplementary material for: Variance estimation for effective coverage measures: A simulation study
Source: J Glob Health. 2020 Mar 14;10(1):010506. doi: 10.7189/jogh-10-010506 (PMC7101480; doi:10.7189/jogh-10-010506)
Supplement: Online Supplementary Document [file jogh-10-010506-s001.zip › jogh-10-010506-s001/Appendix S7.pdf]

Appendix S7: *Non-independence of the p_{rf} estimates within a region r*

For simplicity, consider a random sample of n_{xr} women within region *r*, and assume that there are K facility types in region *r*. In this sample, we observe n_{xr1} women who used facility type 1, n_{xr2} women who used facility type 2, …., n_{xrK} women who used facility type K. Note that the numbers $n_{xr1}, n_{xr2},\ldots,n_{xrK}$ sum up to n_{xr}, and are random numbers (since these numbers change based on the random sample of women in the region) that follow a Multinomial distribution with parameters $n = n_{xr}$, and $P = (P_{xr1}, \ldots P_{xrK})$. The probabilities $P_{xr1}, \ldots P_{xrK}$ add up to 1.

We can estimate P_{yrk}, k = 1,...,K with $p_{yrk} = n_{xrk}/n_{xr}$. Cov(p_{xrk}, $p_{xrk'}$) for k ≠ k' is therefore equal to $Cov(n_{xrk}/n_{xr}, n_{xrk'}/n_{xr}) = 1/(n_{xr})^2 Cov(n_{xrk}, n_{xrk'}) = -P_{xrk}P_{xrk'}/n_{xr}$. Hence, the covariance between the effective coverage estimates for two facility types in the same region *r* is negative.
