# Supplementary material for: Variance estimation for effective coverage measures: A simulation study
Source: J Glob Health. 2020 Mar 14;10(1):010506. doi: 10.7189/jogh-10-010506 (PMC7101480; doi:10.7189/jogh-10-010506)

Appendix S5: Full simulation results with continuous readiness score on (0,1)

In this section, we present simulation results for the continuous readiness/binary coverage setting described in the paper. The simulation scenarios considered are given in Table 1 below:

Appendix S5 Table 1: Simulation Settings

Term	Definition	Simulation settings
P_{xrf}	Proportion of individuals in need accessing service (crude coverage)	(0.02, 0.04, 0.06, 0.08, 0.1, 0.15, 0.2, 0.25, 0.30, 0.35, 0.40, 0.45, 0.50, 0.55, 0.6, 0.65, 0.70, 0.75, 0.80, 0.85, 0.90, 0.92, 0.94, 0.96, 0.98)
P_{yrf}	Proportion of health facilities rated as 'high-quality'	(0.02, 0.04, 0.06, 0.08, 0.1, 0.15, 0.2, 0.25, 0.30, 0.35, 0.40, 0.45, 0.50, 0.55, 0.6, 0.65, 0.70, 0.75, 0.80, 0.85, 0.90, 0.92, 0.94, 0.96, 0.98)
n_{xrf}	Number of individuals sampled	(n_{xrf}, n_{yrf}) = (50, 50), (100, 50), (100, 100), (500, 500)
n_{yrf}	Number of facilities sampled	

Figure 1 shows the estimated coverage probabilities for the exact method, and Figure 2 shows the estimated coverage probabilities for the delta method. Figure 3 shows the proportion of invalid confidence intervals obtained under the exact method, and Figure 4 shows the proportion of iterations in which the delta method yielded undefined confidence limits. Figure 5 shows the proportion of undefined confidence intervals constructed using the delta method. Figure 6 shows the estimated coverage probabilities for the exact method that are calculated by including the iterations that yield degenerate confidence intervals. In all figures, the x-axis corresponds to coverage and the y-axis corresponds to readiness.

Appendix S5 Figure 1: Estimated coverage probability for confidence intervals constructed under the exact method

(a) n_{xrf} = 50, n_{yrf} = 50

	0.02	0.04	0.06	0.08	0.1	0.15	0.2	0.25	0.30	0.35	0.40	0.45	0.50	0.55	0.6	0.65	0.70	0.75	0.80	0.85	0.90	0.92	0.94	0.96	0.98
0.02	1.00	1.00	0.92	0.92	0.91	0.92	0.93	0.93	0.93	0.94	0.94	0.94	0.94	0.94	0.94	0.94	0.94	0.94	0.94	0.94	0.94	0.94	0.94	0.94	0.94
0.04	1.00	1.00	0.91	0.92	0.91	0.92	0.93	0.93	0.94	0.94	0.94	0.94	0.94	0.94	0.94	0.94	0.94	0.94	0.94	0.94	0.94	0.94	0.94	0.94	0.94
0.06	1.00	1.00	0.91	0.93	0.91	0.92	0.93	0.93	0.94	0.94	0.94	0.94	0.94	0.94	0.94	0.94	0.94	0.94	0.94	0.94	0.94	0.94	0.94	0.94	0.94
0.08	1.00	1.00	0.90	0.93	0.90	0.92	0.93	0.93	0.94	0.94	0.94	0.94	0.94	0.94	0.94	0.95	0.95	0.95	0.94	0.95	0.94	0.94	0.94	0.94	0.94
0.1	1.00	1.00	0.90	0.93	0.90	0.92	0.93	0.93	0.94	0.94	0.94	0.94	0.94	0.94	0.94	0.94	0.94	0.94	0.94	0.94	0.94	0.94	0.94	0.94	0.94
0.15	1.00	1.00	0.90	0.93	0.89	0.93	0.93	0.94	0.94	0.94	0.94	0.94	0.94	0.94	0.94	0.94	0.94	0.94	0.94	0.94	0.94	0.94	0.95	0.94	0.95
0.2	1.00	1.00	0.89	0.93	0.89	0.93	0.93	0.93	0.94	0.94	0.94	0.94	0.94	0.94	0.94	0.94	0.94	0.94	0.94	0.94	0.94	0.94	0.94	0.94	0.95
0.25	1.00	1.00	0.89	0.93	0.89	0.93	0.93	0.93	0.94	0.94	0.94	0.94	0.94	0.94	0.94	0.94	0.94	0.94	0.94	0.94	0.94	0.94	0.94	0.94	0.95
0.30	1.00	1.00	0.89	0.93	0.89	0.93	0.93	0.93	0.94	0.94	0.94	0.94	0.94	0.94	0.94	0.94	0.94	0.94	0.94	0.94	0.94	0.94	0.94	0.94	0.94
0.35	1.00	1.00	0.88	0.93	0.89	0.93	0.93	0.94	0.94	0.94	0.94	0.94	0.94	0.94	0.94	0.94	0.94	0.94	0.94	0.94	0.93	0.93	0.93	0.93	0.94
0.40	1.00	1.00	0.88	0.93	0.89	0.93	0.93	0.94	0.94	0.94	0.94	0.94	0.94	0.94	0.94	0.94	0.94	0.94	0.94	0.93	0.93	0.92	0.92	0.93	0.93
0.45	1.00	1.00	0.87	0.93	0.89	0.94	0.93	0.94	0.94	0.94	0.94	0.94	0.94	0.94	0.94	0.94	0.94	0.94	0.93	0.93	0.93	0.92	0.92	0.92	0.93
0.50	1.00	1.00	0.87	0.93	0.89	0.94	0.94	0.94	0.94	0.94	0.94	0.94	0.94	0.94	0.94	0.94	0.94	0.94	0.93	0.93	0.93	0.92	0.92	0.92	0.93
0.55	1.00	1.00	0.87	0.93	0.89	0.94	0.94	0.94	0.94	0.94	0.94	0.94	0.94	0.94	0.94	0.94	0.94	0.94	0.93	0.93	0.93	0.92	0.91	0.91	0.92
0.6	1.00	1.00	0.86	0.93	0.89	0.94	0.94	0.94	0.94	0.94	0.94	0.94	0.94	0.94	0.94	0.94	0.94	0.94	0.93	0.93	0.92	0.92	0.91	0.90	0.91
0.65	1.00	1.00	0.86	0.93	0.89	0.94	0.94	0.94	0.94	0.94	0.94	0.94	0.94	0.94	0.94	0.94	0.94	0.93	0.93	0.93	0.92	0.92	0.91	0.89	0.90
0.70	1.00	1.00	0.86	0.93	0.89	0.94	0.94	0.94	0.94	0.94	0.94	0.94	0.94	0.94	0.94	0.94	0.94	0.94	0.93	0.93	0.92	0.91	0.90	0.88	0.88
0.75	1.00	1.00	0.85	0.93	0.89	0.94	0.94	0.94	0.94	0.94	0.94	0.94	0.94	0.94	0.94	0.94	0.94	0.94	0.93	0.93	0.91	0.91	0.90	0.87	0.85
0.80	1.00	1.00	0.85	0.93	0.89	0.94	0.94	0.94	0.94	0.94	0.94	0.94	0.94	0.94	0.94	0.94	0.94	0.94	0.93	0.93	0.91	0.91	0.90	0.87	0.81
0.85	1.00	1.00	0.85	0.93	0.89	0.94	0.94	0.94	0.94	0.94	0.94	0.94	0.94	0.94	0.94	0.94	0.94	0.94	0.93	0.93	0.91	0.91	0.89	0.87	0.76
0.90	1.00	1.00	0.85	0.93	0.89	0.94	0.94	0.94	0.94	0.94	0.94	0.94	0.94	0.94	0.94	0.94	0.94	0.94	0.93	0.93	0.90	0.91	0.88	0.87	0.69
0.92	1.00	1.00	0.85	0.93	0.89	0.94	0.94	0.94	0.94	0.94	0.94	0.94	0.94	0.94	0.94	0.94	0.94	0.94	0.93	0.93	0.90	0.91	0.88	0.87	0.67
0.94	1.00	1.00	0.85	0.93	0.89	0.94	0.94	0.94	0.94	0.94	0.94	0.94	0.93	0.94	0.94	0.94	0.94	0.94	0.94	0.93	0.89	0.91	0.88	0.87	0.65
0.96	1.00	1.00	0.85	0.93	0.89	0.94	0.94	0.94	0.94	0.94	0.94	0.94	0.93	0.94	0.94	0.94	0.94	0.94	0.94	0.94	0.89	0.91	0.87	0.87	0.64
0.98	1.00	1.00	0.85	0.93	0.89	0.94	0.94	0.94	0.94	0.94	0.94	0.94	0.93	0.94	0.94	0.94	0.94	0.94	0.94	0.94	0.88	0.91	0.86	0.87	0.64

(b) n_{xrf} = 100, n_{yrf} = 50

	0.02	0.04	0.06	0.08	0.1	0.15	0.2	0.25	0.30	0.35	0.40	0.45	0.50	0.55	0.6	0.65	0.70	0.75	0.80	0.85	0.90	0.92	0.94	0.96	0.98
0.02	1.00	0.91	0.91	0.92	0.92	0.93	0.93	0.94	0.94	0.94	0.94	0.94	0.94	0.94	0.94	0.94	0.95	0.94	0.94	0.94	0.94	0.94	0.94	0.94	0.94
0.04	1.00	0.92	0.92	0.92	0.93	0.94	0.94	0.94	0.94	0.94	0.94	0.94	0.94	0.94	0.94	0.94	0.95	0.94	0.94	0.94	0.94	0.94	0.94	0.94	0.94
0.06	1.00	0.93	0.92	0.92	0.93	0.93	0.94	0.94	0.94	0.94	0.94	0.95	0.95	0.94	0.95	0.94	0.95	0.94	0.94	0.94	0.94	0.94	0.94	0.94	0.94
0.08	1.00	0.93	0.92	0.92	0.93	0.94	0.94	0.94	0.94	0.94	0.94	0.95	0.95	0.95	0.95	0.95	0.95	0.94	0.94	0.94	0.94	0.94	0.94	0.94	0.94
0.1	1.00	0.93	0.92	0.92	0.93	0.94	0.94	0.94	0.94	0.95	0.95	0.95	0.95	0.95	0.95	0.95	0.95	0.95	0.94	0.94	0.94	0.94	0.94	0.94	0.94
0.15	1.00	0.93	0.93	0.92	0.93	0.94	0.94	0.94	0.94	0.95	0.95	0.95	0.95	0.94	0.95	0.95	0.95	0.95	0.95	0.95	0.95	0.95	0.95	0.95	0.95
0.2	1.00	0.93	0.93	0.92	0.93	0.94	0.94	0.94	0.94	0.95	0.95	0.95	0.95	0.95	0.94	0.94	0.95	0.95	0.95	0.95	0.95	0.95	0.95	0.95	0.95
0.25	1.00	0.93	0.93	0.92	0.93	0.94	0.94	0.94	0.94	0.95	0.95	0.95	0.95	0.95	0.95	0.95	0.95	0.95	0.95	0.95	0.95	0.95	0.95	0.95	0.95
0.30	1.00	0.92	0.94	0.91	0.93	0.94	0.94	0.94	0.94	0.95	0.95	0.95	0.95	0.95	0.95	0.95	0.95	0.94	0.94	0.94	0.95	0.94	0.95	0.95	0.95
0.35	1.00	0.92	0.94	0.91	0.93	0.93	0.94	0.94	0.94	0.95	0.95	0.95	0.95	0.95	0.95	0.95	0.95	0.94	0.94	0.94	0.94	0.94	0.94	0.94	0.94
0.40	1.00	0.92	0.94	0.91	0.93	0.93	0.94	0.94	0.94	0.95	0.95	0.95	0.95	0.95	0.95	0.95	0.95	0.94	0.94	0.94	0.94	0.94	0.94	0.94	0.94
0.45	1.00	0.92	0.94	0.91	0.93	0.93	0.94	0.94	0.94	0.94	0.95	0.95	0.95	0.95	0.95	0.95	0.95	0.94	0.94	0.94	0.94	0.94	0.94	0.94	0.94
0.50	1.00	0.92	0.94	0.91	0.93	0.93	0.94	0.94	0.94	0.95	0.95	0.95	0.94	0.95	0.95	0.94	0.95	0.94	0.95	0.94	0.94	0.94	0.94	0.94	0.94
0.55	1.00	0.92	0.94	0.91	0.93	0.93	0.94	0.94	0.95	0.95	0.95	0.95	0.95	0.95	0.95	0.95	0.94	0.95	0.94	0.94	0.94	0.94	0.94	0.94	0.95
0.6	1.00	0.92	0.94	0.91	0.93	0.93	0.94	0.94	0.95	0.95	0.95	0.95	0.95	0.95	0.95	0.95	0.94	0.94	0.94	0.94	0.94	0.94	0.94	0.94	0.94
0.65	1.00	0.92	0.94	0.91	0.93	0.93	0.94	0.94	0.94	0.95	0.95	0.95	0.95	0.95	0.95	0.95	0.94	0.94	0.94	0.94	0.94	0.94	0.94	0.94	0.94
0.70	1.00	0.92	0.94	0.90	0.93	0.93	0.94	0.94	0.95	0.95	0.95	0.95	0.95	0.95	0.95	0.95	0.94	0.94	0.94	0.94	0.94	0.94	0.93	0.93	0.94
0.75	1.00	0.92	0.94	0.90	0.93	0.93	0.94	0.94	0.95	0.95	0.95	0.95	0.95	0.95	0.95	0.95	0.94	0.94	0.94	0.94	0.94	0.93	0.93	0.93	0.93
0.80	1.00	0.92	0.94	0.90	0.93	0.93	0.94	0.94	0.94	0.95	0.95	0.95	0.95	0.95	0.95	0.95	0.94	0.94	0.94	0.94	0.93	0.93	0.93	0.92	0.91
0.85	1.00	0.92	0.94	0.90	0.93	0.93	0.93	0.94	0.95	0.95	0.95	0.95	0.95	0.95	0.94	0.95	0.94	0.94	0.94	0.94	0.93	0.93	0.92	0.92	0.90
0.90	1.00	0.92	0.94	0.90	0.93	0.93	0.93	0.94	0.95	0.94	0.95	0.95	0.95	0.95	0.95	0.95	0.94	0.94	0.94	0.94	0.93	0.93	0.92	0.91	0.88
0.92	1.00	0.92	0.94	0.90	0.93	0.93	0.93	0.94	0.95	0.94	0.95	0.95	0.94	0.95	0.95	0.95	0.94	0.94	0.94	0.94	0.93	0.93	0.92	0.91	0.87
0.94	1.00	0.92	0.94	0.90	0.93	0.93	0.93	0.94	0.95	0.94	0.94	0.95	0.94	0.95	0.95	0.95	0.94	0.94	0.94	0.94	0.93	0.92	0.92	0.91	0.87
0.96	1.00	0.92	0.94	0.90	0.93	0.93	0.93	0.94	0.95	0.94	0.95	0.95	0.94	0.95	0.95	0.95	0.95	0.94	0.94	0.94	0.93	0.92	0.92	0.91	0.87
0.98	1.00	0.92	0.94	0.90	0.93	0.93	0.93	0.94	0.95	0.94	0.95	0.94	0.94	0.95	0.94	0.94	0.94	0.94	0.94	0.93	0.93	0.91	0.93	0.91	0.87

(c) $n_{xrf} = 100, n_{yrf} = 100$

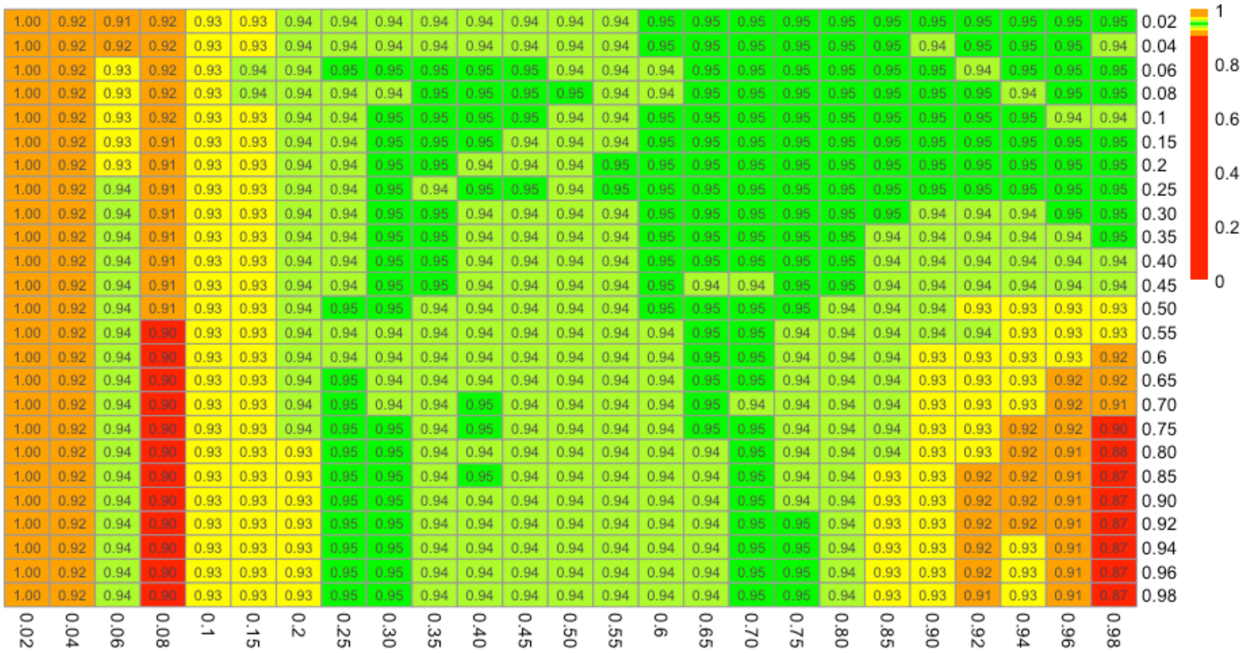

(d) $n_{xrf} = 500, n_{yrf} = 500$

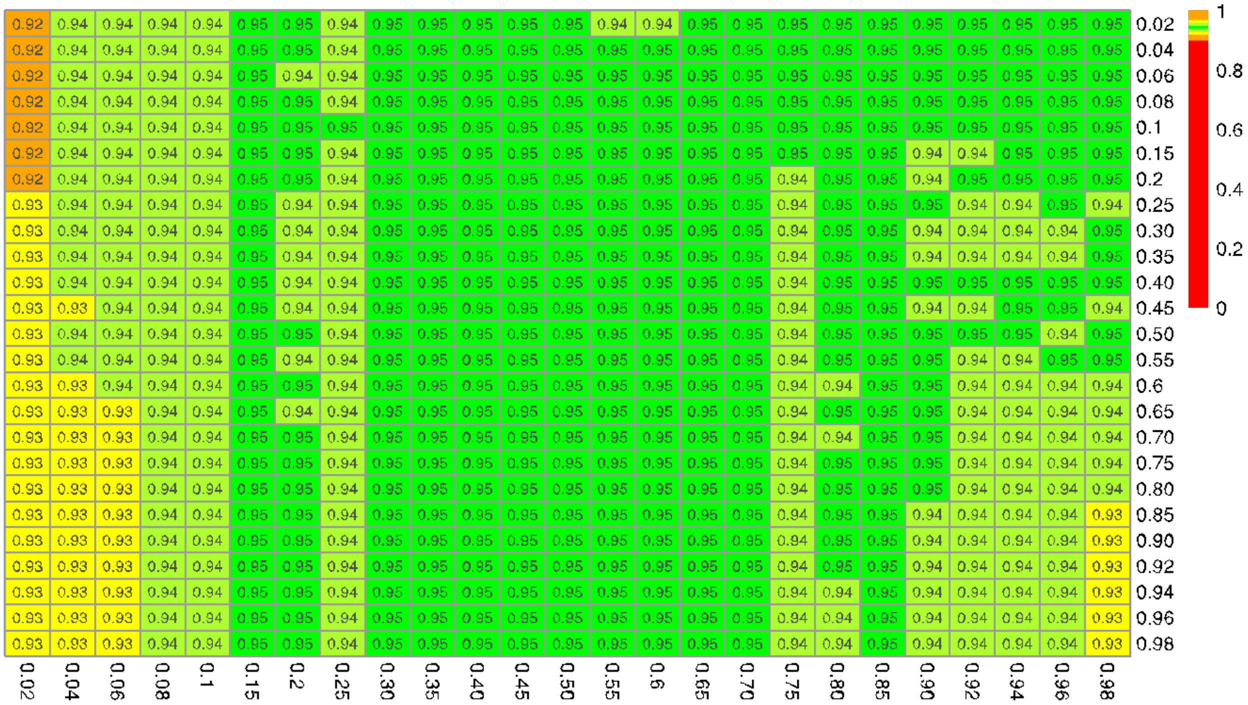

Appendix S5 Figure 2: Estimated coverage probability for confidence intervals constructed under the delta method

(a) n_{xrf} = 50, n_{yrf} = 50

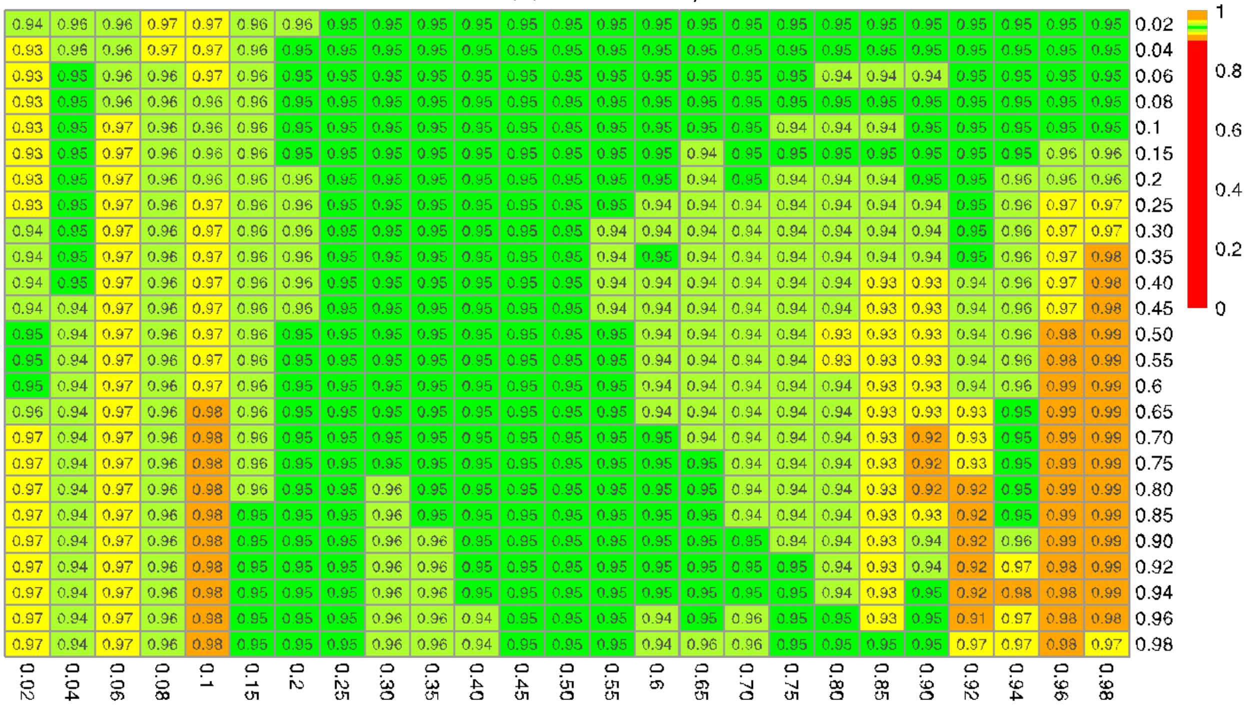

(b) n_{xrf} = 100, n_{yrf} = 50

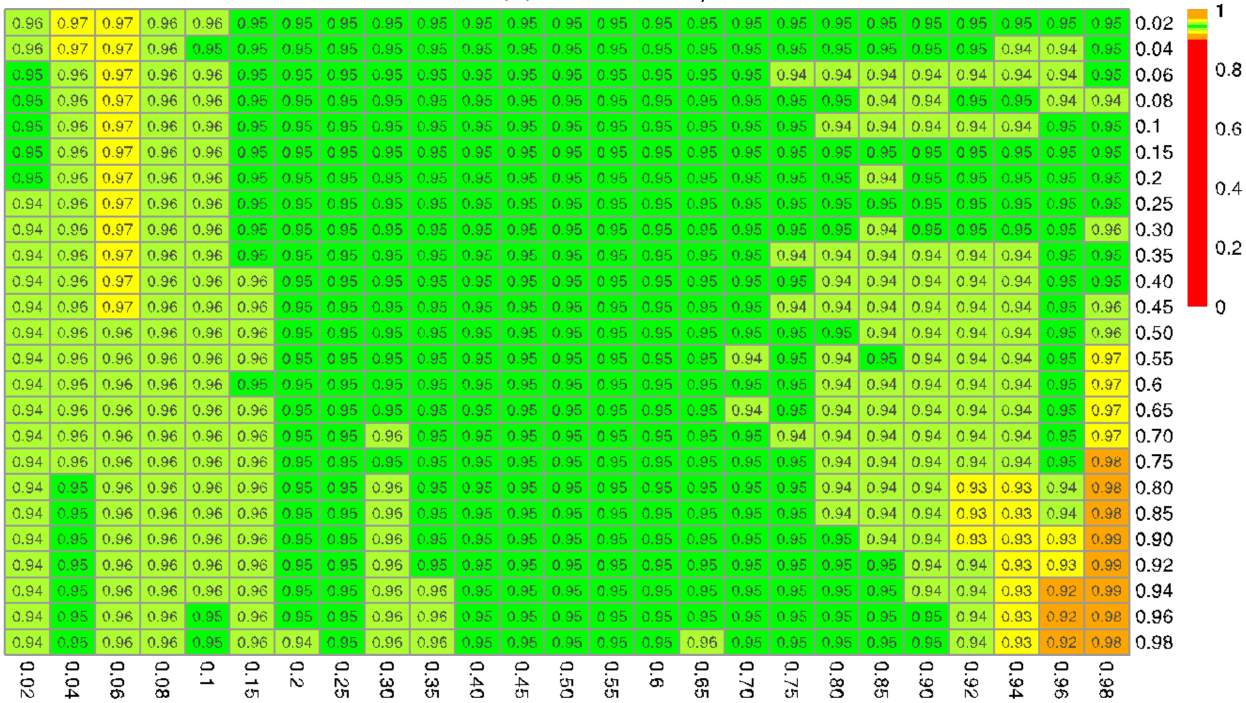

(c) n_{xrf} = 100, n_{yrf} = 100

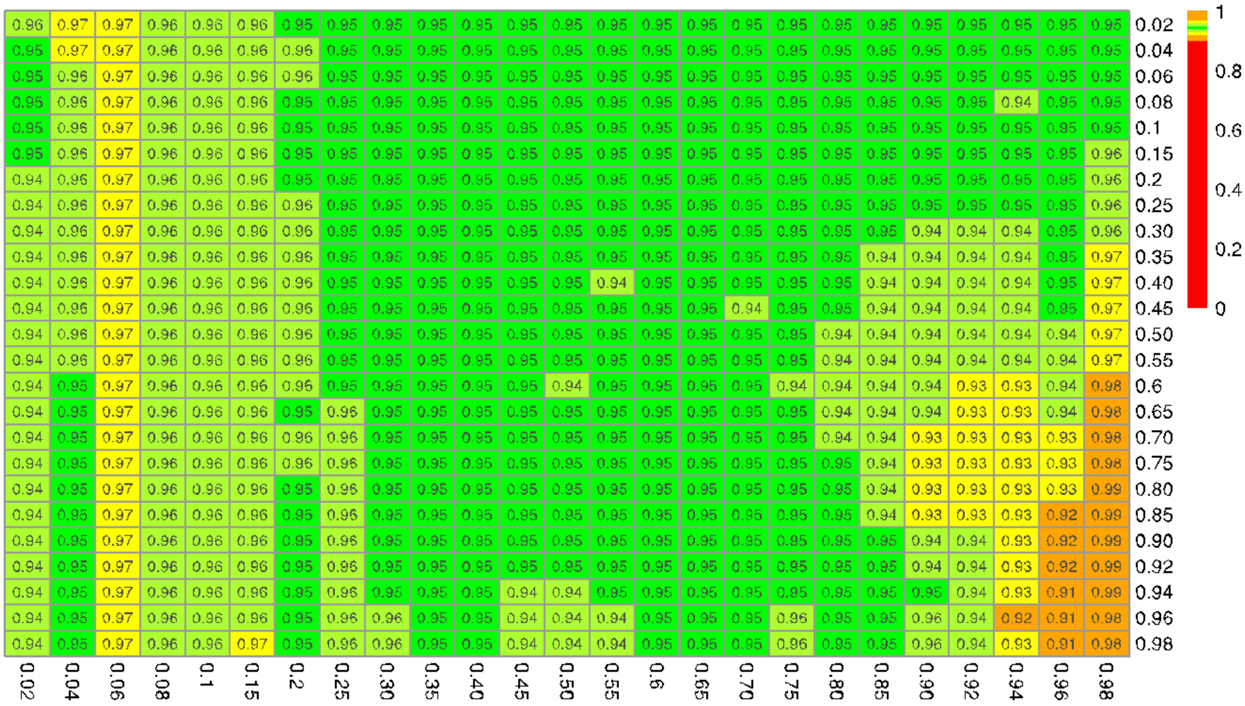

(d) n_{xrf} = 500, n_{yrf} = 500

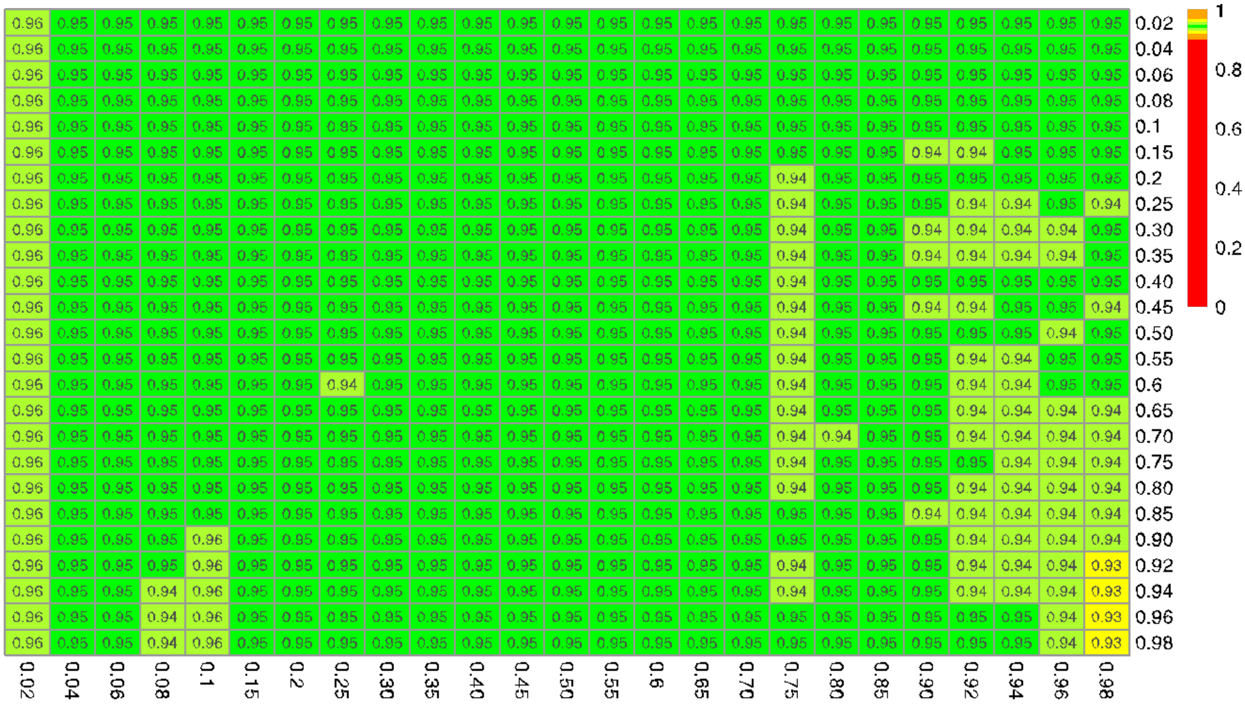

Appendix S5 Figure 3: Estimated coverage probability for confidence intervals constructed under the parametric bootstrap method

(a) n_{xrf} = 50, n_{yrf} = 50

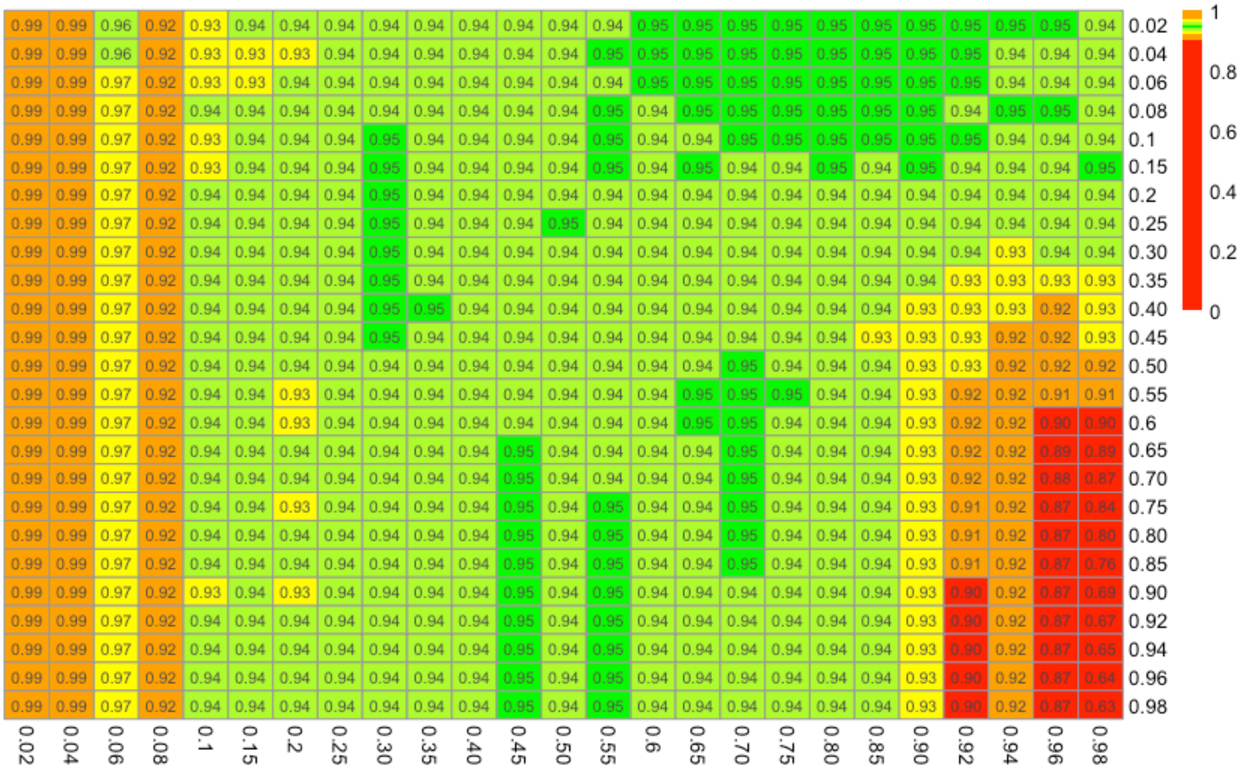

(b) n_{xrf} = 100, n_{yrf} = 50

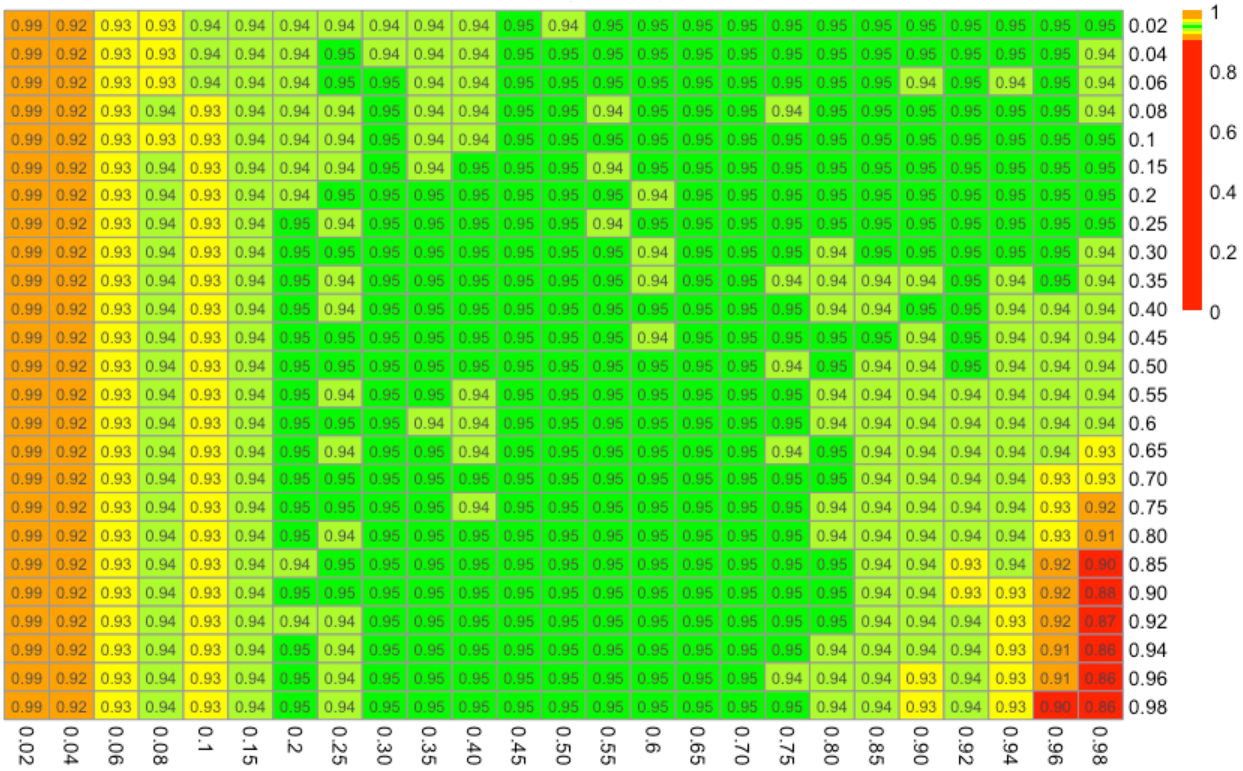

(c) n_{xrf} = 100, n_{yrf} = 100

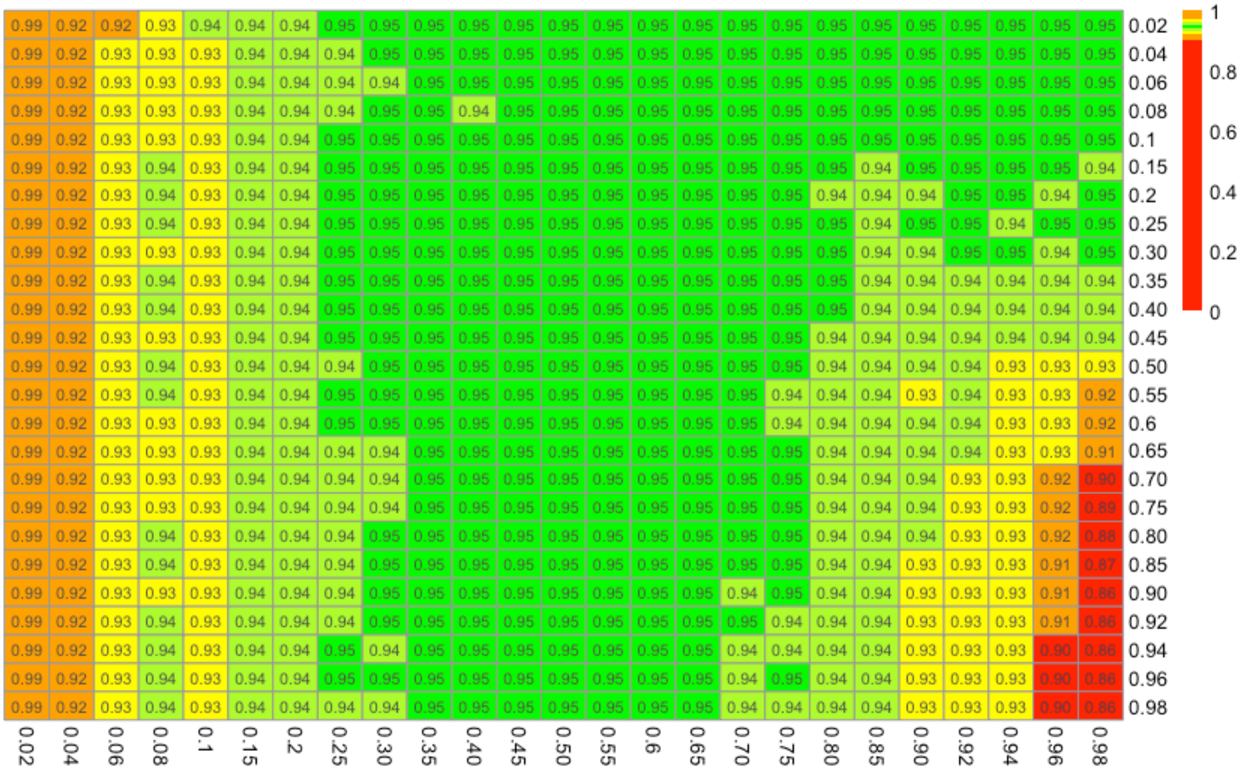

(e) n_{xrf} = 500, n_{yrf} = 500

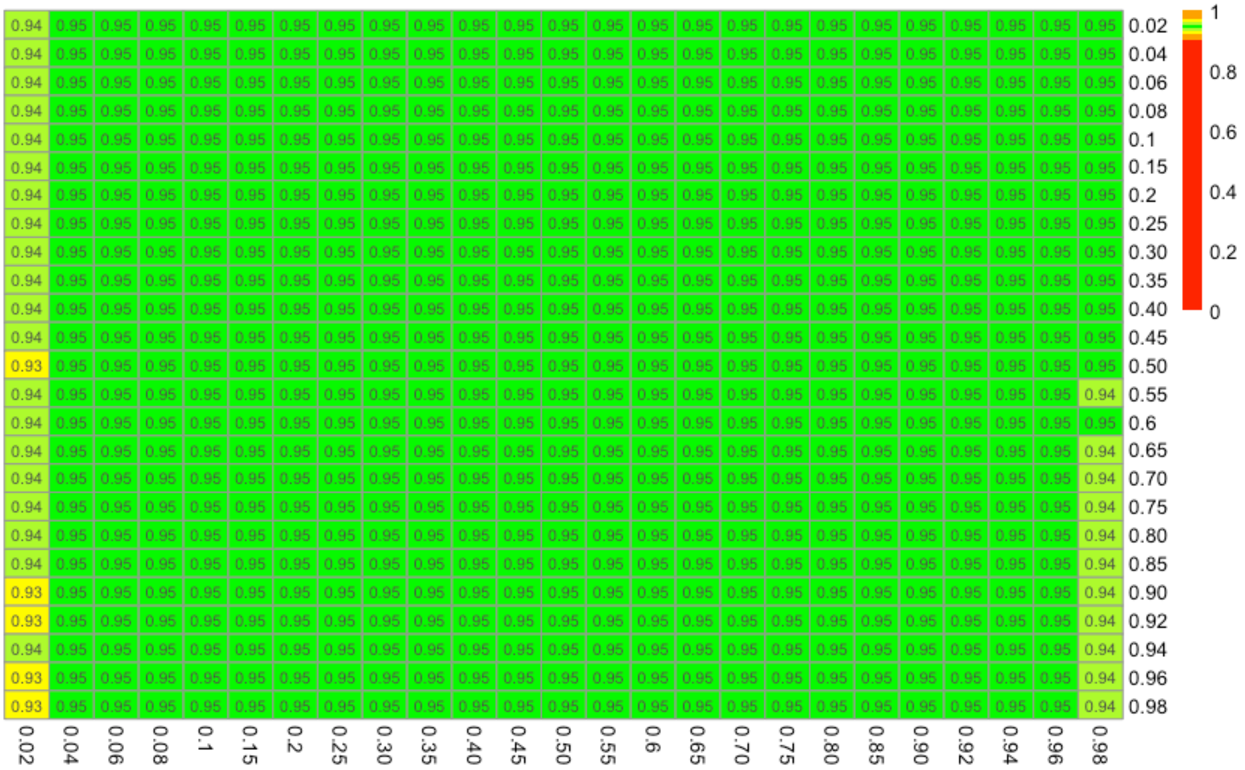

Appendix S5 Figure 4: Proportion of invalid confidence interval constructed using the exact method

(a) n_{xrf} = 50, n_{yrf} = 50

	0.02	0.04	0.06	0.08	0.1	0.15	0.2	0.25	0.30	0.35	0.40	0.45	0.50	0.55	0.6	0.65	0.70	0.75	0.80	0.85	0.90	0.92	0.94	0.96	0.98
0.02	0.98	0.86	0.66	0.45	0.28	0.06	0.01	0.00	0.00	0.00	0.00	0.00	0.00	0.00	0.00	0.00	0.00	0.00	0.00	0.00	0.00	0.00	0.00	0.00	0.00
0.04	0.97	0.84	0.63	0.42	0.24	0.05	0.01	0.00	0.00	0.00	0.00	0.00	0.00	0.00	0.00	0.00	0.00	0.00	0.00	0.00	0.00	0.00	0.00	0.00	0.00
0.06	0.97	0.84	0.63	0.42	0.24	0.05	0.01	0.00	0.00	0.00	0.00	0.00	0.00	0.00	0.00	0.00	0.00	0.00	0.00	0.00	0.00	0.00	0.00	0.00	0.00
0.08	0.97	0.84	0.63	0.42	0.24	0.05	0.01	0.00	0.00	0.00	0.00	0.00	0.00	0.00	0.00	0.00	0.00	0.00	0.00	0.00	0.00	0.00	0.00	0.00	0.00
0.1	0.97	0.84	0.63	0.42	0.24	0.05	0.01	0.00	0.00	0.00	0.00	0.00	0.00	0.00	0.00	0.00	0.00	0.00	0.00	0.00	0.00	0.00	0.00	0.00	0.00
0.15	0.97	0.84	0.63	0.42	0.24	0.05	0.01	0.00	0.00	0.00	0.00	0.00	0.00	0.00	0.00	0.00	0.00	0.00	0.00	0.00	0.00	0.00	0.00	0.00	0.00
0.2	0.97	0.84	0.63	0.42	0.24	0.05	0.01	0.00	0.00	0.00	0.00	0.00	0.00	0.00	0.00	0.00	0.00	0.00	0.00	0.00	0.00	0.00	0.00	0.00	0.00
0.25	0.97	0.84	0.63	0.42	0.24	0.05	0.01	0.00	0.00	0.00	0.00	0.00	0.00	0.00	0.00	0.00	0.00	0.00	0.00	0.00	0.00	0.00	0.00	0.00	0.00
0.30	0.97	0.84	0.63	0.42	0.24	0.05	0.01	0.00	0.00	0.00	0.00	0.00	0.00	0.00	0.00	0.00	0.00	0.00	0.00	0.00	0.00	0.00	0.00	0.00	0.00
0.35	0.97	0.84	0.63	0.42	0.24	0.05	0.01	0.00	0.00	0.00	0.00	0.00	0.00	0.00	0.00	0.00	0.00	0.00	0.00	0.00	0.00	0.00	0.00	0.00	0.00
0.40	0.97	0.84	0.63	0.42	0.24	0.05	0.01	0.00	0.00	0.00	0.00	0.00	0.00	0.00	0.00	0.00	0.00	0.00	0.00	0.00	0.00	0.00	0.00	0.00	0.00
0.45	0.97	0.84	0.63	0.42	0.24	0.05	0.01	0.00	0.00	0.00	0.00	0.00	0.00	0.00	0.00	0.00	0.00	0.00	0.00	0.00	0.00	0.00	0.00	0.00	0.00
0.50	0.97	0.84	0.63	0.42	0.24	0.05	0.01	0.00	0.00	0.00	0.00	0.00	0.00	0.00	0.00	0.00	0.00	0.00	0.00	0.00	0.00	0.00	0.00	0.00	0.00
0.55	0.97	0.84	0.63	0.42	0.24	0.05	0.01	0.00	0.00	0.00	0.00	0.00	0.00	0.00	0.00	0.00	0.00	0.00	0.00	0.00	0.00	0.00	0.00	0.00	0.00
0.6	0.97	0.84	0.63	0.42	0.24	0.05	0.01	0.00	0.00	0.00	0.00	0.00	0.00	0.00	0.00	0.00	0.00	0.00	0.00	0.00	0.00	0.00	0.00	0.00	0.00
0.65	0.97	0.84	0.63	0.42	0.24	0.05	0.01	0.00	0.00	0.00	0.00	0.00	0.00	0.00	0.00	0.00	0.00	0.00	0.00	0.00	0.00	0.00	0.00	0.00	0.00
0.70	0.97	0.84	0.63	0.42	0.24	0.05	0.01	0.00	0.00	0.00	0.00	0.00	0.00	0.00	0.00	0.00	0.00	0.00	0.00	0.00	0.00	0.00	0.00	0.00	0.00
0.75	0.97	0.84	0.63	0.42	0.24	0.05	0.01	0.00	0.00	0.00	0.00	0.00	0.00	0.00	0.00	0.00	0.00	0.00	0.00	0.00	0.00	0.00	0.00	0.00	0.00
0.80	0.97	0.84	0.63	0.42	0.24	0.05	0.01	0.00	0.00	0.00	0.00	0.00	0.00	0.00	0.00	0.00	0.00	0.00	0.00	0.00	0.00	0.00	0.00	0.00	0.00
0.85	0.97	0.84	0.63	0.42	0.24	0.05	0.01	0.00	0.00	0.00	0.00	0.00	0.00	0.00	0.00	0.00	0.00	0.00	0.00	0.00	0.00	0.00	0.00	0.00	0.00
0.90	0.97	0.84	0.63	0.42	0.24	0.05	0.01	0.00	0.00	0.00	0.00	0.00	0.00	0.00	0.00	0.00	0.00	0.00	0.00	0.00	0.00	0.00	0.00	0.00	0.00
0.92	0.97	0.84	0.63	0.42	0.24	0.05	0.01	0.00	0.00	0.00	0.00	0.00	0.00	0.00	0.00	0.00	0.00	0.00	0.00	0.00	0.00	0.00	0.00	0.00	0.00
0.94	0.97	0.84	0.63	0.42	0.24	0.05	0.01	0.00	0.00	0.00	0.00	0.00	0.00	0.00	0.00	0.00	0.00	0.00	0.00	0.00	0.00	0.00	0.00	0.00	0.00
0.96	0.97	0.84	0.63	0.42	0.24	0.05	0.01	0.00	0.00	0.00	0.00	0.00	0.00	0.00	0.00	0.00	0.00	0.00	0.00	0.00	0.00	0.00	0.00	0.00	0.00
0.98	0.97	0.84	0.63	0.42	0.24	0.05	0.01	0.00	0.00	0.00	0.00	0.00	0.00	0.00	0.00	0.00	0.00	0.00	0.00	0.00	0.01	0.02	0.05	0.10	0.13

(b) n_{xrf} = 100, n_{yrf} = 50

	0.02	0.04	0.06	0.08	0.1	0.15	0.2	0.25	0.30	0.35	0.40	0.45	0.50	0.55	0.6	0.65	0.70	0.75	0.80	0.85	0.90	0.92	0.94	0.96	0.98
0.02	0.92	0.57	0.24	0.08	0.02	0.00	0.00	0.00	0.00	0.00	0.00	0.00	0.00	0.00	0.00	0.00	0.00	0.00	0.00	0.00	0.00	0.00	0.00	0.00	0.00
0.04	0.84	0.42	0.14	0.04	0.01	0.00	0.00	0.00	0.00	0.00	0.00	0.00	0.00	0.00	0.00	0.00	0.00	0.00	0.00	0.00	0.00	0.00	0.00	0.00	0.00
0.06	0.84	0.42	0.14	0.04	0.01	0.00	0.00	0.00	0.00	0.00	0.00	0.00	0.00	0.00	0.00	0.00	0.00	0.00	0.00	0.00	0.00	0.00	0.00	0.00	0.00
0.08	0.84	0.42	0.14	0.04	0.01	0.00	0.00	0.00	0.00	0.00	0.00	0.00	0.00	0.00	0.00	0.00	0.00	0.00	0.00	0.00	0.00	0.00	0.00	0.00	0.00
0.1	0.84	0.42	0.14	0.04	0.01	0.00	0.00	0.00	0.00	0.00	0.00	0.00	0.00	0.00	0.00	0.00	0.00	0.00	0.00	0.00	0.00	0.00	0.00	0.00	0.00
0.15	0.84	0.42	0.14	0.04	0.01	0.00	0.00	0.00	0.00	0.00	0.00	0.00	0.00	0.00	0.00	0.00	0.00	0.00	0.00	0.00	0.00	0.00	0.00	0.00	0.00
0.2	0.84	0.42	0.14	0.04	0.01	0.00	0.00	0.00	0.00	0.00	0.00	0.00	0.00	0.00	0.00	0.00	0.00	0.00	0.00	0.00	0.00	0.00	0.00	0.00	0.00
0.25	0.84	0.42	0.14	0.04	0.01	0.00	0.00	0.00	0.00	0.00	0.00	0.00	0.00	0.00	0.00	0.00	0.00	0.00	0.00	0.00	0.00	0.00	0.00	0.00	0.00
0.30	0.84	0.42	0.14	0.04	0.01	0.00	0.00	0.00	0.00	0.00	0.00	0.00	0.00	0.00	0.00	0.00	0.00	0.00	0.00	0.00	0.00	0.00	0.00	0.00	0.00
0.35	0.84	0.42	0.14	0.04	0.01	0.00	0.00	0.00	0.00	0.00	0.00	0.00	0.00	0.00	0.00	0.00	0.00	0.00	0.00	0.00	0.00	0.00	0.00	0.00	0.00
0.40	0.84	0.42	0.14	0.04	0.01	0.00	0.00	0.00	0.00	0.00	0.00	0.00	0.00	0.00	0.00	0.00	0.00	0.00	0.00	0.00	0.00	0.00	0.00	0.00	0.00
0.45	0.84	0.42	0.14	0.04	0.01	0.00	0.00	0.00	0.00	0.00	0.00	0.00	0.00	0.00	0.00	0.00	0.00	0.00	0.00	0.00	0.00	0.00	0.00	0.00	0.00
0.50	0.84	0.42	0.14	0.04	0.01	0.00	0.00	0.00	0.00	0.00	0.00	0.00	0.00	0.00	0.00	0.00	0.00	0.00	0.00	0.00	0.00	0.00	0.00	0.00	0.00
0.55	0.84	0.42	0.14	0.04	0.01	0.00	0.00	0.00	0.00	0.00	0.00	0.00	0.00	0.00	0.00	0.00	0.00	0.00	0.00	0.00	0.00	0.00	0.00	0.00	0.00
0.6	0.84	0.42	0.14	0.04	0.01	0.00	0.00	0.00	0.00	0.00	0.00	0.00	0.00	0.00	0.00	0.00	0.00	0.00	0.00	0.00	0.00	0.00	0.00	0.00	0.00
0.65	0.84	0.42	0.14	0.04	0.01	0.00	0.00	0.00	0.00	0.00	0.00	0.00	0.00	0.00	0.00	0.00	0.00	0.00	0.00	0.00	0.00	0.00	0.00	0.00	0.00
0.70	0.84	0.42	0.14	0.04	0.01	0.00	0.00	0.00	0.00	0.00	0.00	0.00	0.00	0.00	0.00	0.00	0.00	0.00	0.00	0.00	0.00	0.00	0.00	0.00	0.00
0.75	0.84	0.42	0.14	0.04	0.01	0.00	0.00	0.00	0.00	0.00	0.00	0.00	0.00	0.00	0.00	0.00	0.00	0.00	0.00	0.00	0.00	0.00	0.00	0.00	0.00
0.80	0.84	0.42	0.14	0.04	0.01	0.00	0.00	0.00	0.00	0.00	0.00	0.00	0.00	0.00	0.00	0.00	0.00	0.00	0.00	0.00	0.00	0.00	0.00	0.00	0.00
0.85	0.84	0.42	0.14	0.04	0.01	0.00	0.00	0.00	0.00	0.00	0.00	0.00	0.00	0.00	0.00	0.00	0.00	0.00	0.00	0.00	0.00	0.00	0.00	0.00	0.00
0.90	0.84	0.42	0.14	0.04	0.01	0.00	0.00	0.00	0.00	0.00	0.00	0.00	0.00	0.00	0.00	0.00	0.00	0.00	0.00	0.00	0.00	0.00	0.00	0.00	0.00
0.92	0.84	0.42	0.14	0.04	0.01	0.00	0.00	0.00	0.00	0.00	0.00	0.00	0.00	0.00	0.00	0.00	0.00	0.00	0.00	0.00	0.00	0.00	0.00	0.00	0.00
0.94	0.84	0.42	0.14	0.04	0.01	0.00	0.00	0.00	0.00	0.00	0.00	0.00	0.00	0.00	0.00	0.00	0.00	0.00	0.00	0.00	0.00	0.00	0.00	0.00	0.00
0.96	0.84	0.42	0.14	0.04	0.01	0.00	0.00	0.00	0.00	0.00	0.00	0.00	0.00	0.00	0.00	0.00	0.00	0.00	0.00	0.00	0.00	0.00	0.00	0.00	0.00
0.98	0.84	0.42	0.14	0.04	0.01	0.00	0.00	0.00	0.00	0.00	0.00	0.00	0.00	0.00	0.00	0.00	0.00	0.00	0.00	0.00	0.00	0.00	0.00	0.00	0.00

(c) $n_{xrf} = 100$, $n_{yrf} = 100$

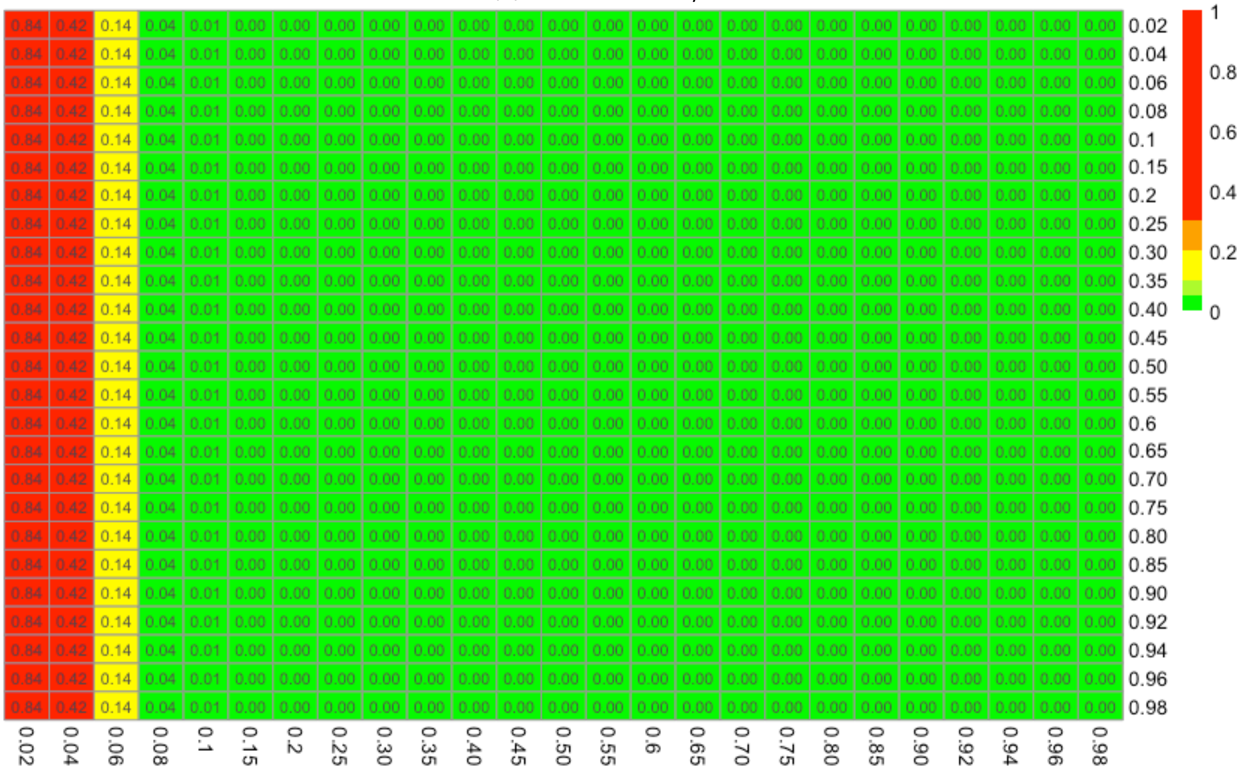

(d) $n_{xrf} = 500$, $n_{yrf} = 500$

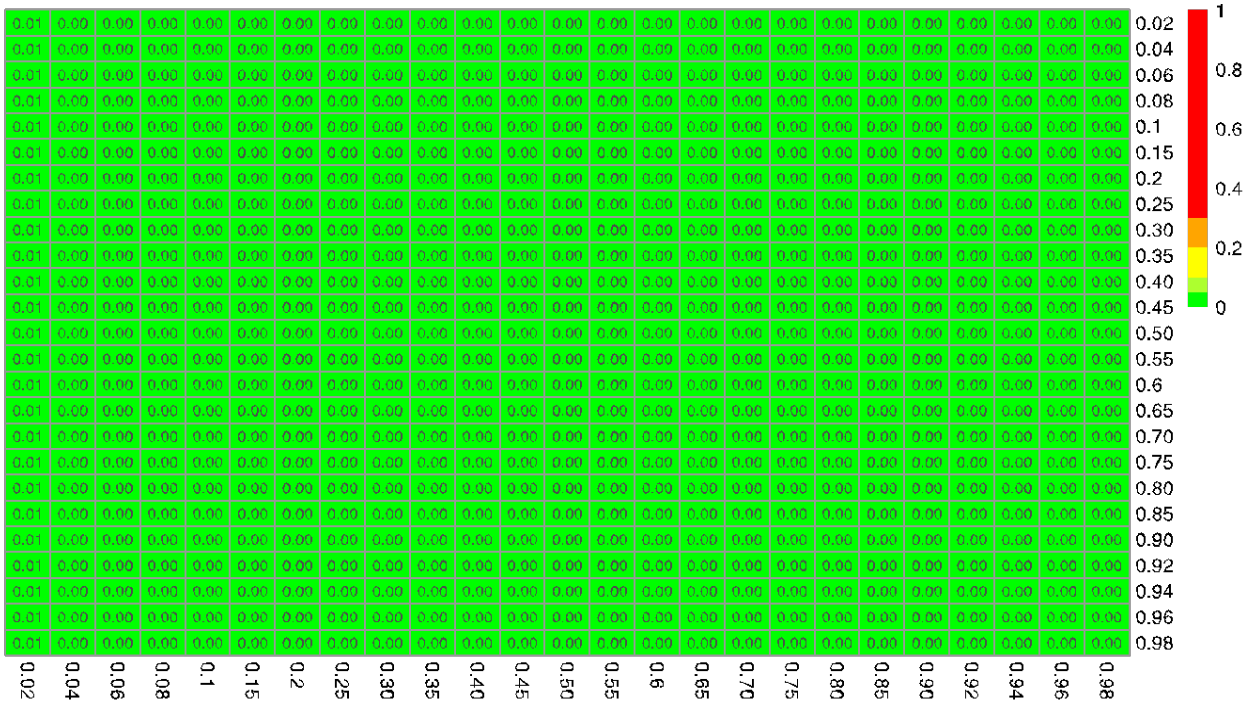

Appendix S5 Figure 5: Proportion of undefined confidence intervals constructed using the delta method

(a) n_{xrf} = 50, n_{yrf} = 50

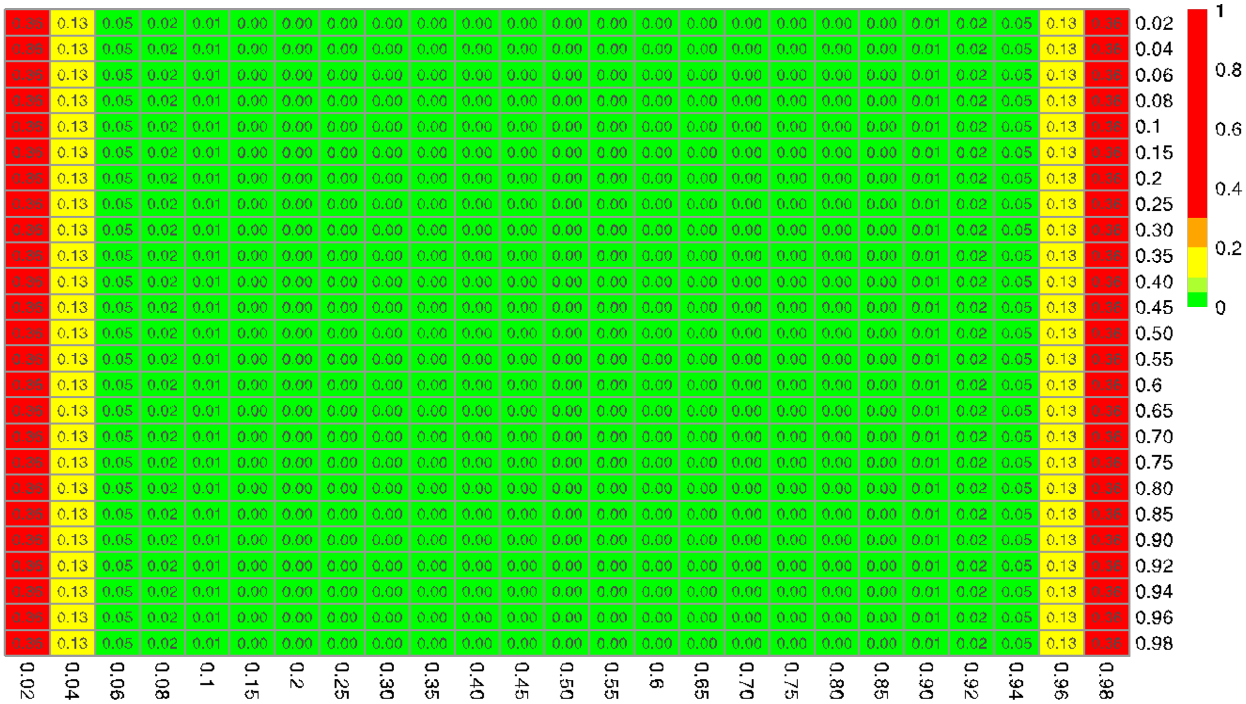

(b) n_{xrf} = 100, n_{yrf} = 50

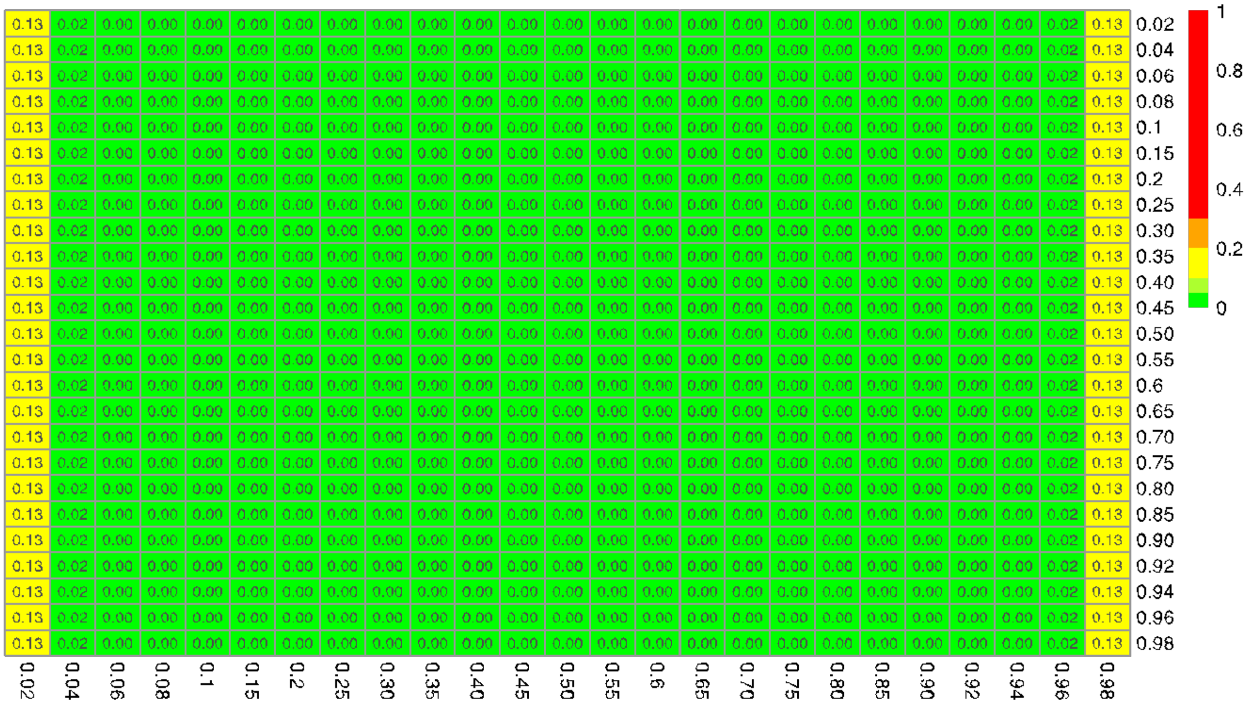

(c) n_{xrf} = 100, n_{yrf} = 100

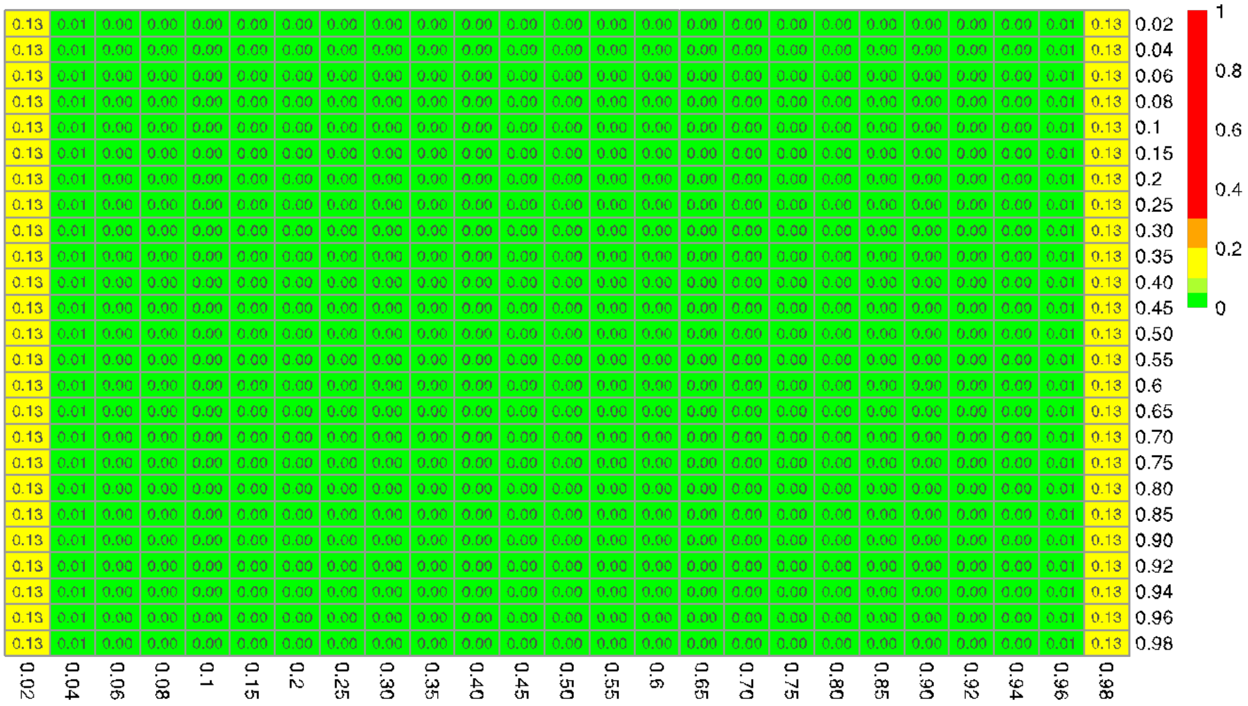

(d) n_{xrf} = 500, n_{yrf} = 500

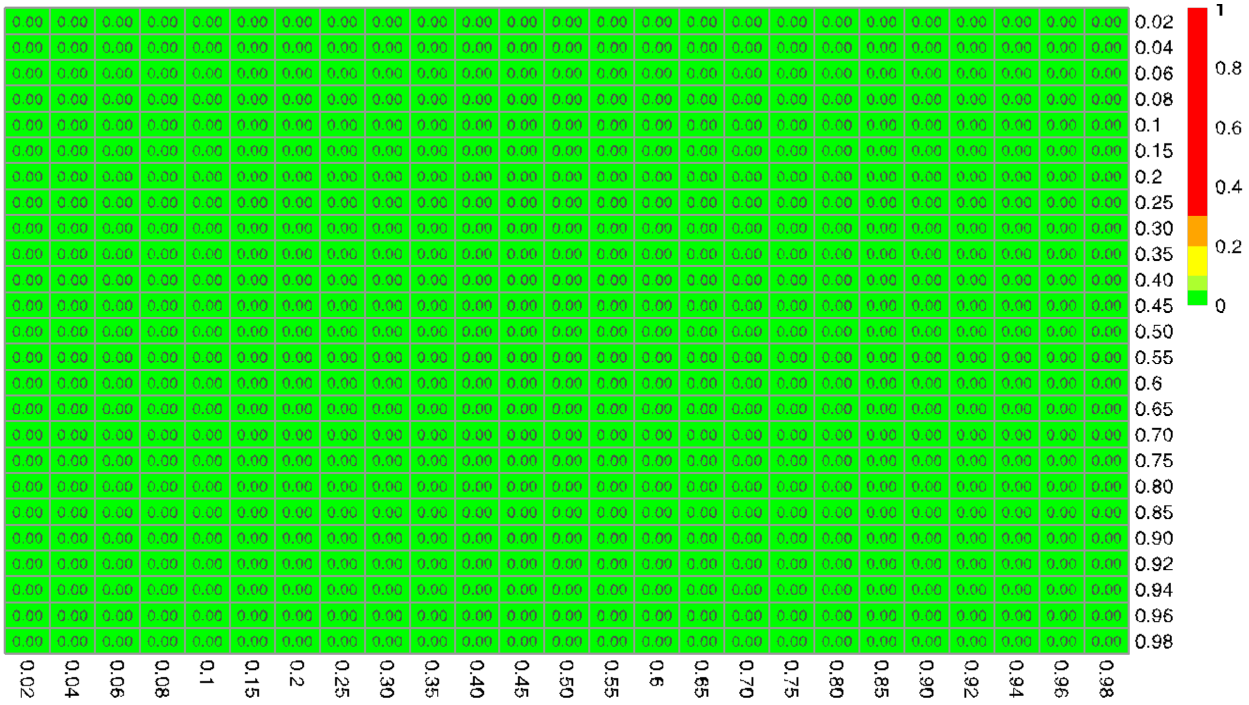

Appendix S5 Figure 6: Estimated coverage probability for confidence intervals constructed under the exact method when including the degenerate intervals

(a) n_{xrf} = 50, n_{yrf} = 50

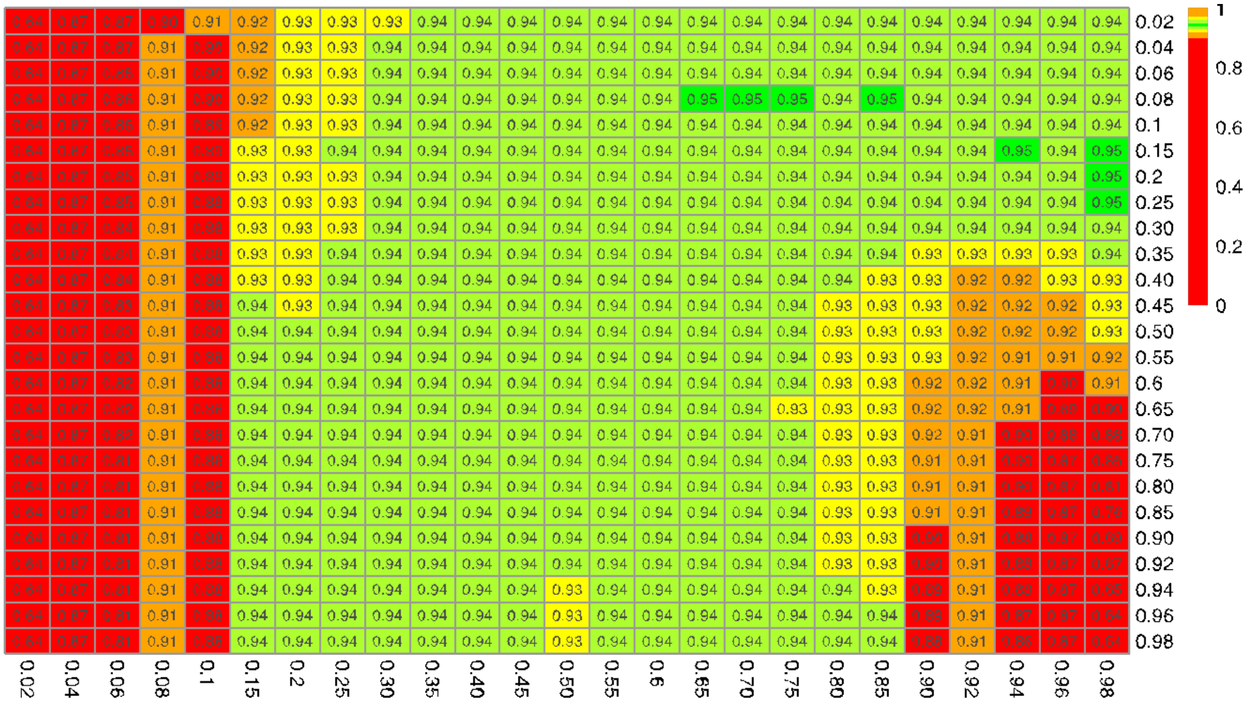

Supplement: Online Supplementary Document [file jogh-10-010506-s001.zip › jogh-10-010506-s001/Appendix S5.pdf]
