# Supplementary material for: Variance estimation for effective coverage measures: A simulation study
Source: J Glob Health. 2020 Mar 14;10(1):010506. doi: 10.7189/jogh-10-010506 (PMC7101480; doi:10.7189/jogh-10-010506)
Supplement: Online Supplementary Document [file jogh-10-010506-s001.zip › jogh-10-010506-s001/Appendix S4.pdf]

Appendix S4. Full simulation results, binary readiness and binary coverage indicators

In this section, we present the full simulation results for the binary readiness/binary coverage setting described in the paper. The simulation scenarios considered are given in Table 1 below:

Table 1: Simulation Settings

Term	Definition	Simulation settings
P_{xrf}	Proportion of individuals in need accessing service (crude coverage)	(0.02, 0.04, 0.06, 0.08, 0.1, 0.15, 0.2, 0.25, 0.30, 0.35, 0.40, 0.45, 0.50, 0.55, 0.6, 0.65, 0.70, 0.75, 0.80, 0.85, 0.90, 0.92, 0.94, 0.96, 0.98)
P_{yrf}	Proportion of health facilities rated as 'high-quality'	(0.02, 0.04, 0.06, 0.08, 0.1, 0.15, 0.2, 0.25, 0.30, 0.35, 0.40, 0.45, 0.50, 0.55, 0.6, 0.65, 0.70, 0.75, 0.80, 0.85, 0.90, 0.92, 0.94, 0.96, 0.98)
n_{xrf}	Number of individuals sampled	(n_{xrf}, n_{yrf}) = (50, 50), (100, 100), (200, 200), (300, 300), (400, 400), (500, 500), (100, 50), (200, 50), (300, 50), (400, 50), (500, 50)
n_{yrf}	Number of facilities sampled	

Figure 1 shows the estimated coverage probabilities for the exact method, *Figure 2* shows the estimated coverage probabilities for the delta method, and *Figure 3* shows the estimated coverage probabilities for the parametric bootstrap method. *Figure 4* shows the proportion of invalid confidence intervals obtained under the exact method, and *Figure 5* shows the proportion of iterations in which the delta method yielded undefined confidence limits. *Figure 6* shows the estimated coverage probabilities for the exact method that are calculated when including the iterations that yield degenerate confidence intervals for one simulation setting. In all figures, the x-axis corresponds to coverage and the y-axis corresponds to readiness.

Appendix S4 Figure 1: Estimated 95% coverage probabilities for the confidence intervals constructed using the exact method.

(a) $n_{xrf} = 50$, $n_{yrf} = 50$

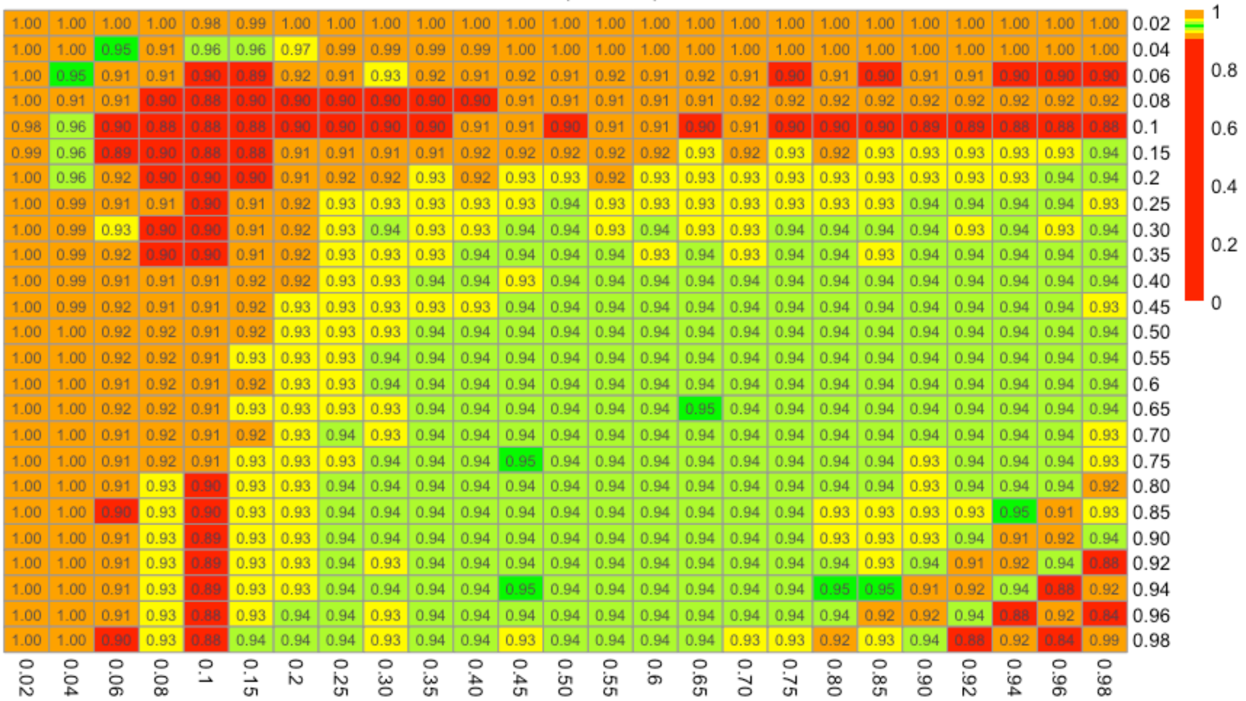

(b) $n_{xrf} = 100$, $n_{yrf} = 50$

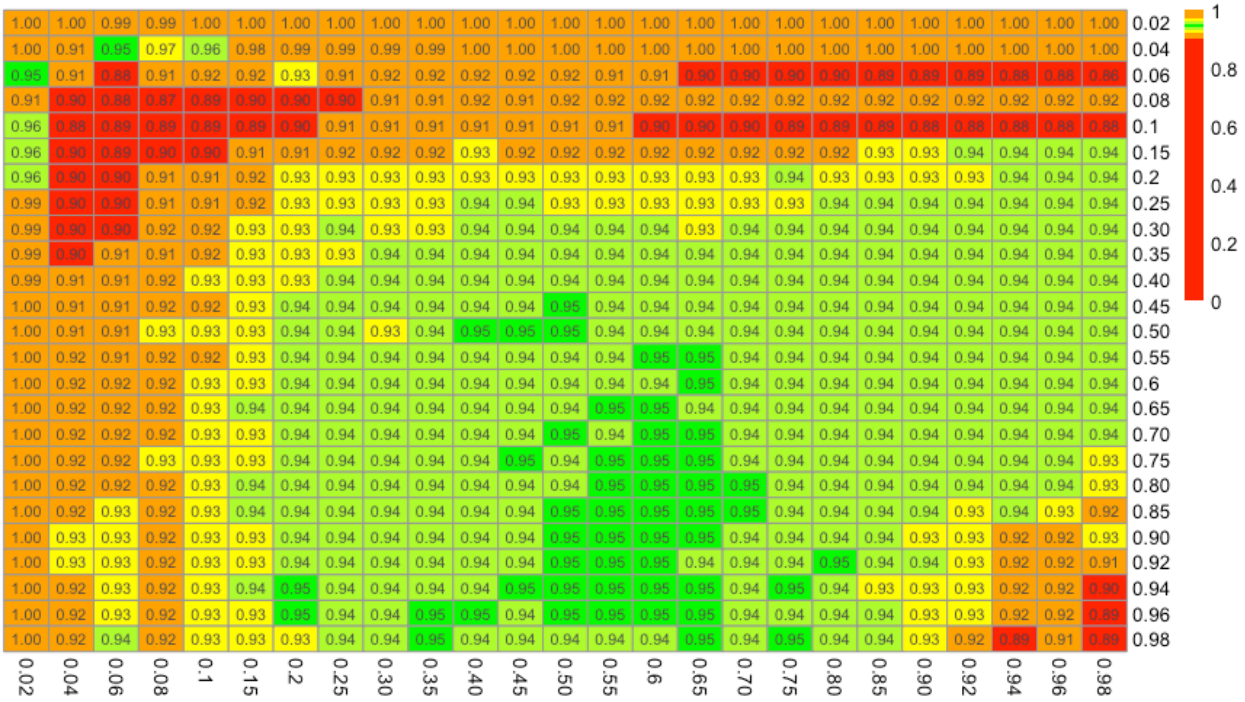

(c) n_{xrf} = 100, n_{yrf} = 100

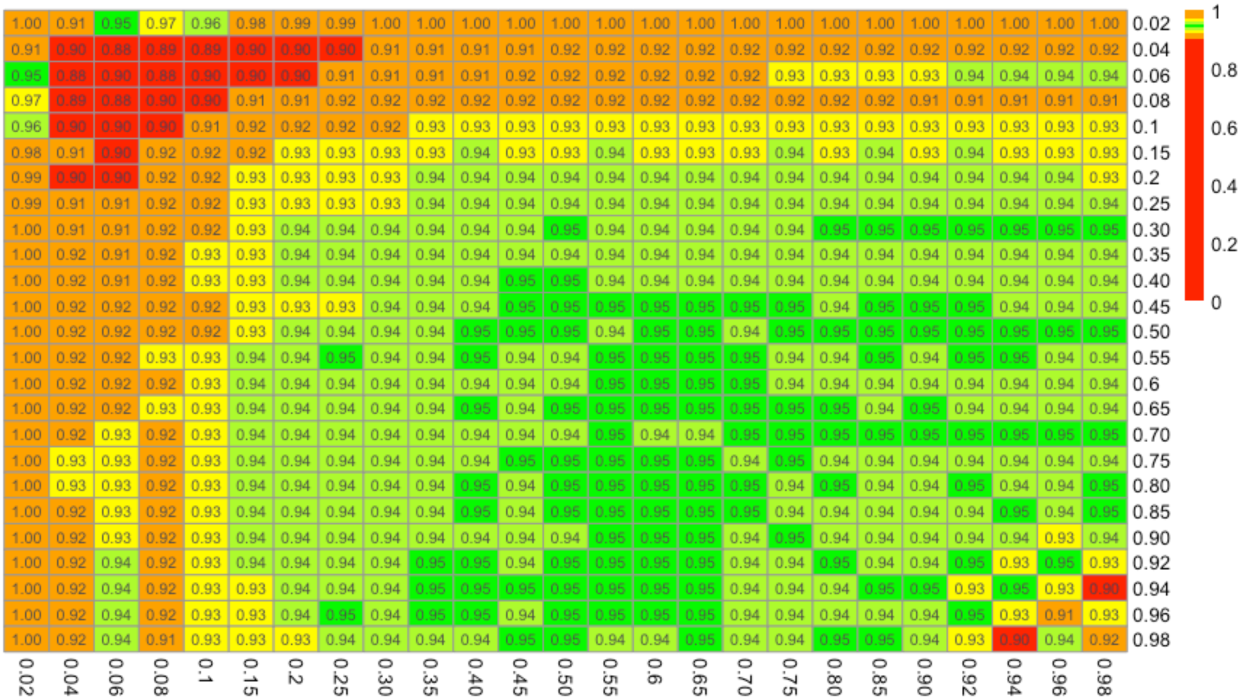

(d) n_{xrf} = 200, n_{yrf} = 50

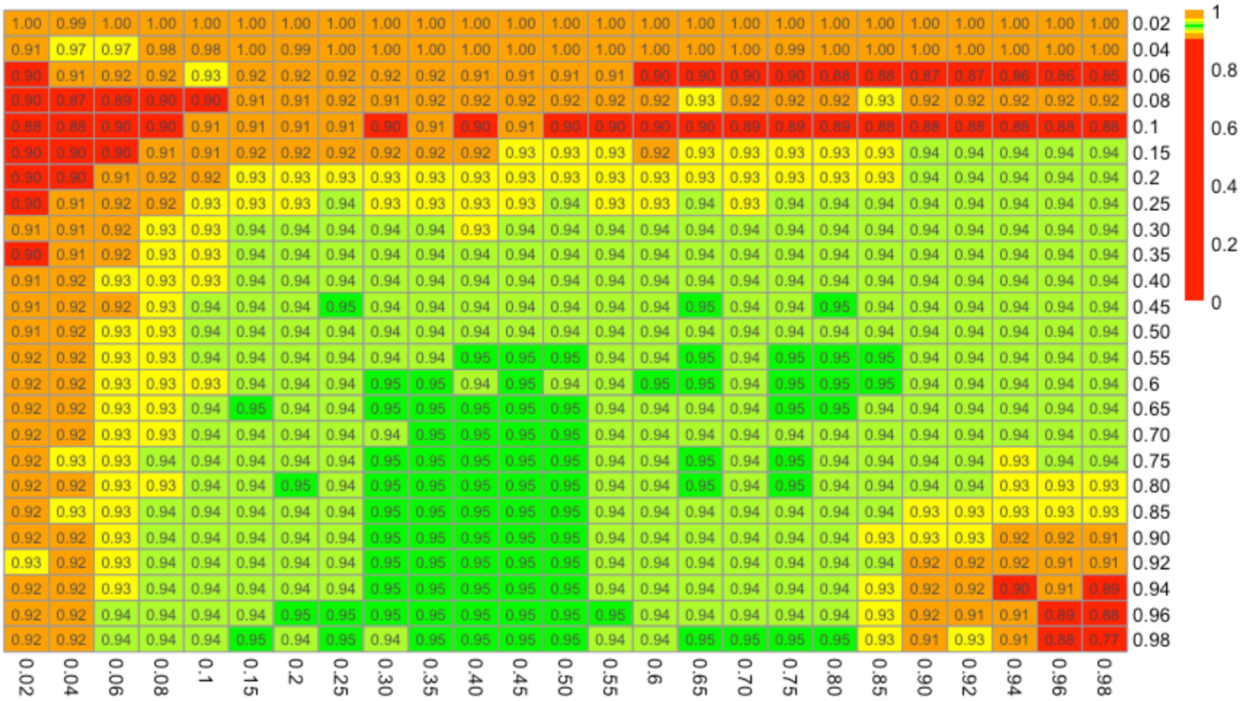

(e) $n_{xrf} = 200$, $n_{yrf} = 200$

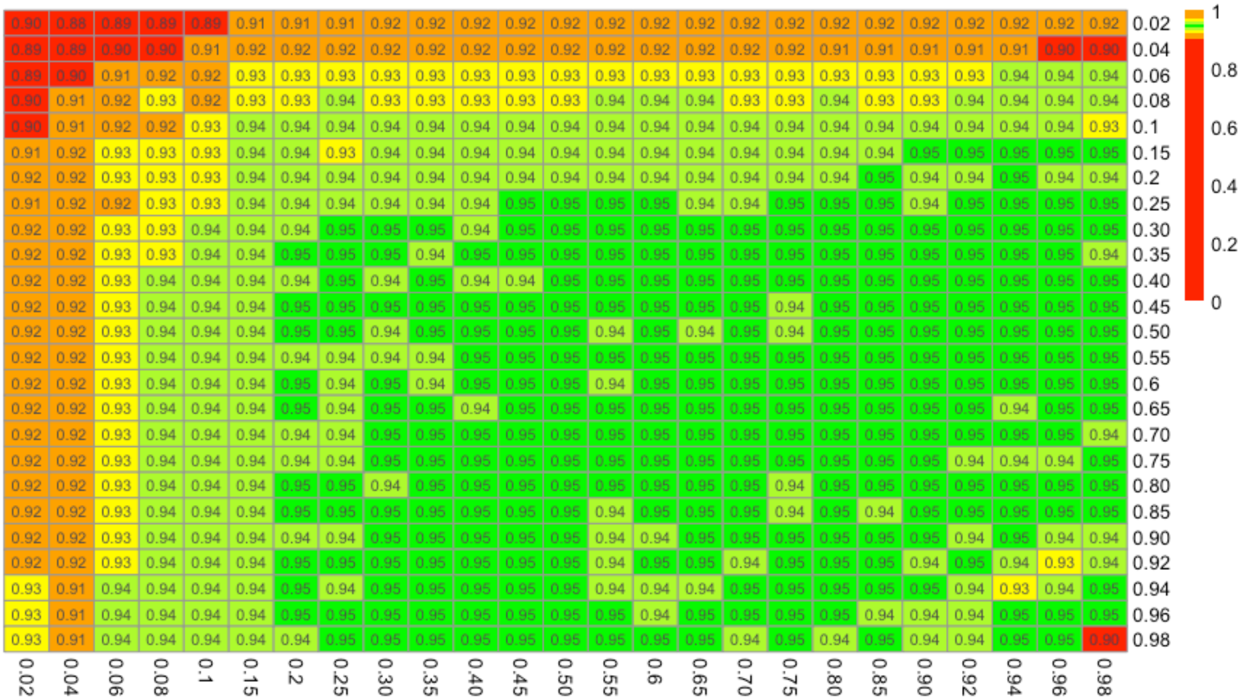

(f) $n_{xrf}=300$, $n_{yrf}=50$

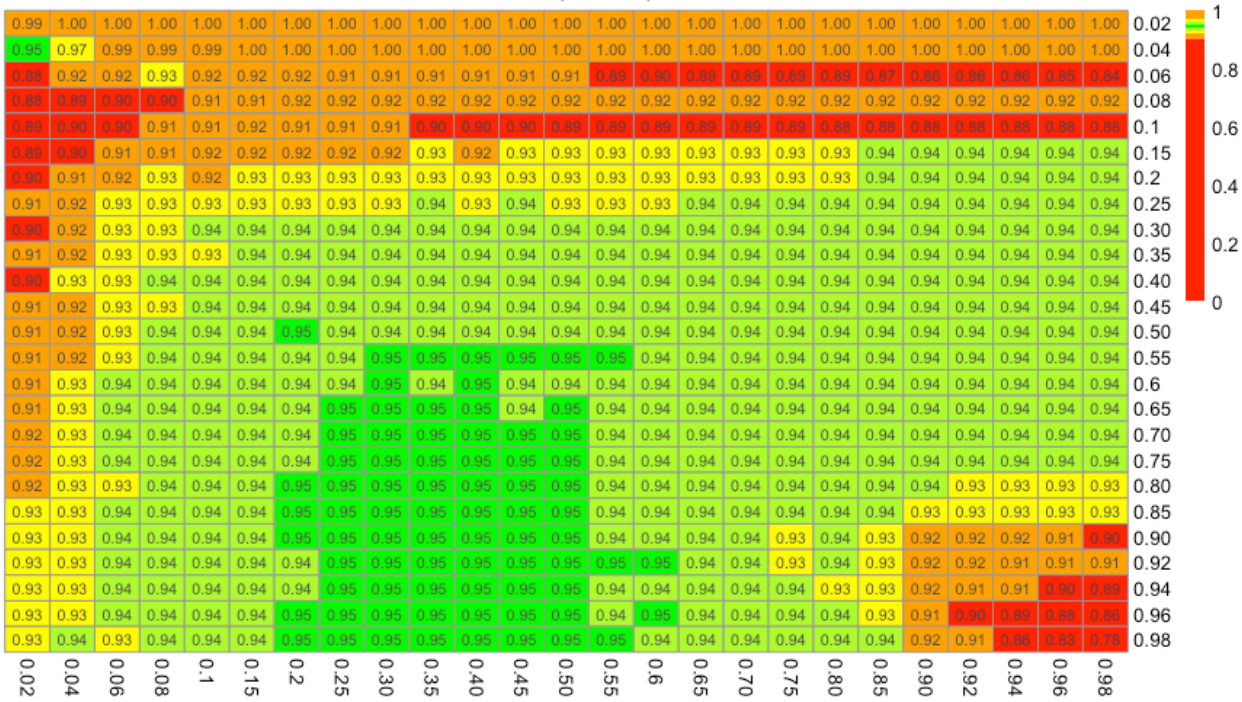

(g) n_{xrf} = 300, n_{yrf} = 300

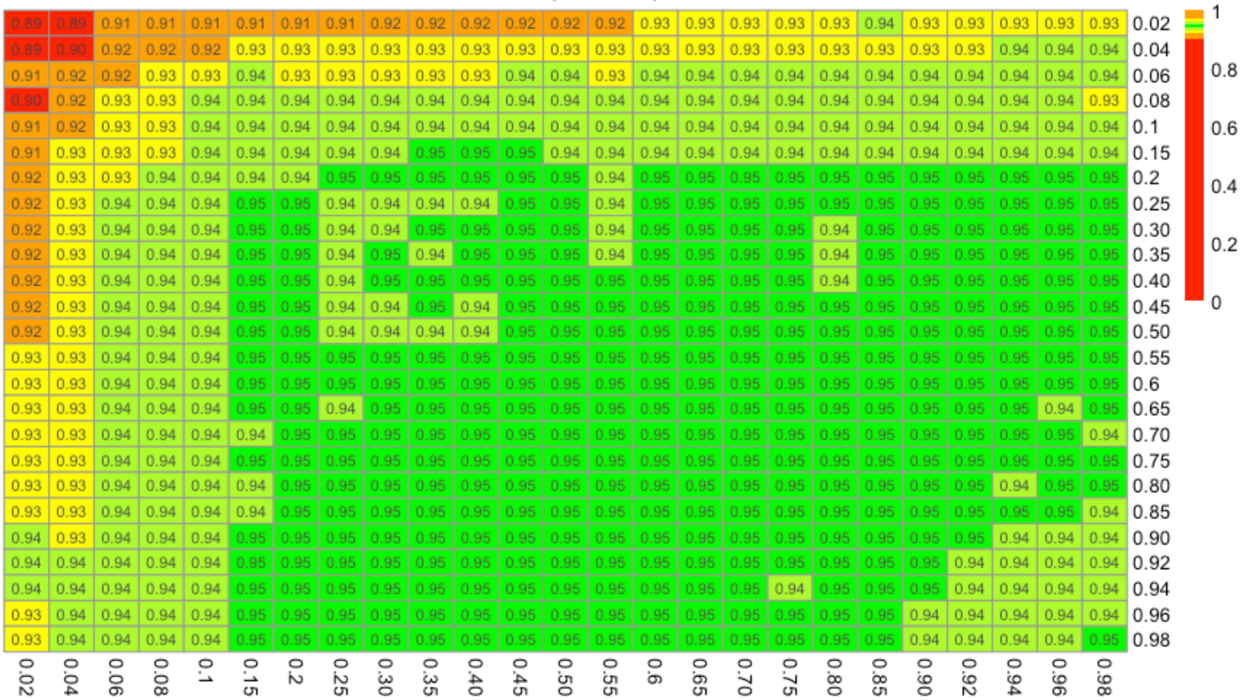

(h) n_{xrf} = 400, n_{yrf} = 50

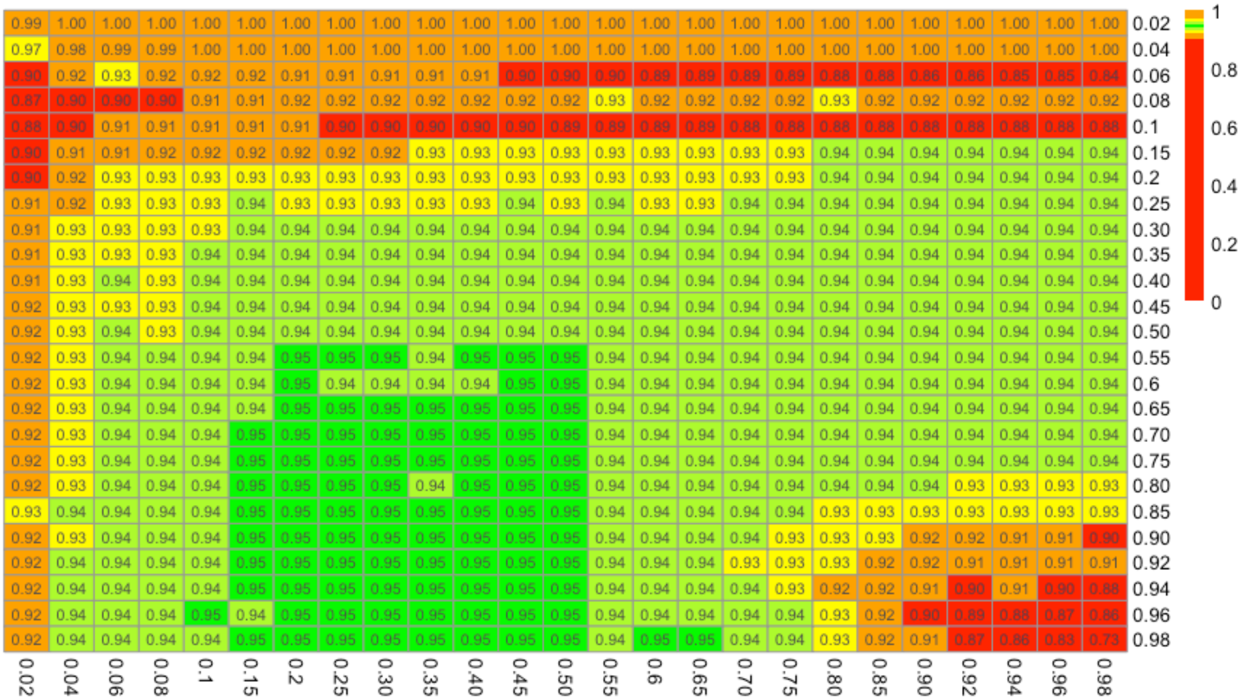

(i) $n_{xrf} = 400$, $n_{yrf} = 400$

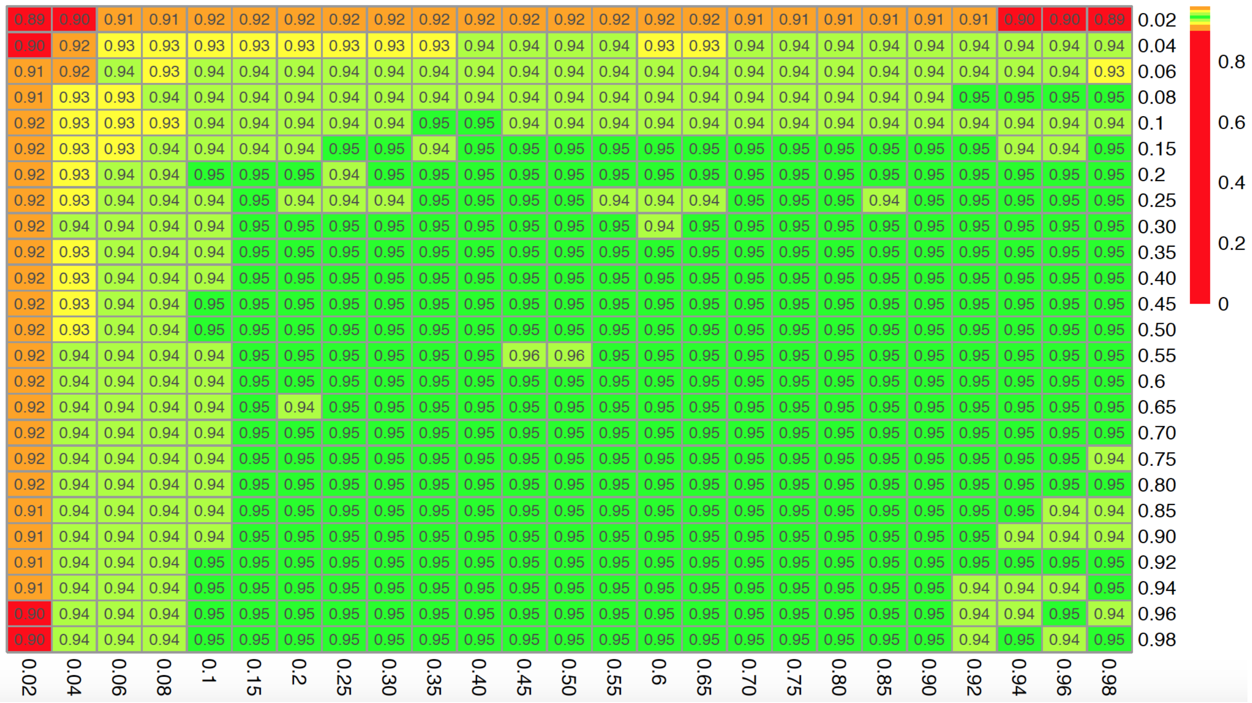

(j) $n_{xrf} = 500$, $n_{yrf} = 50$

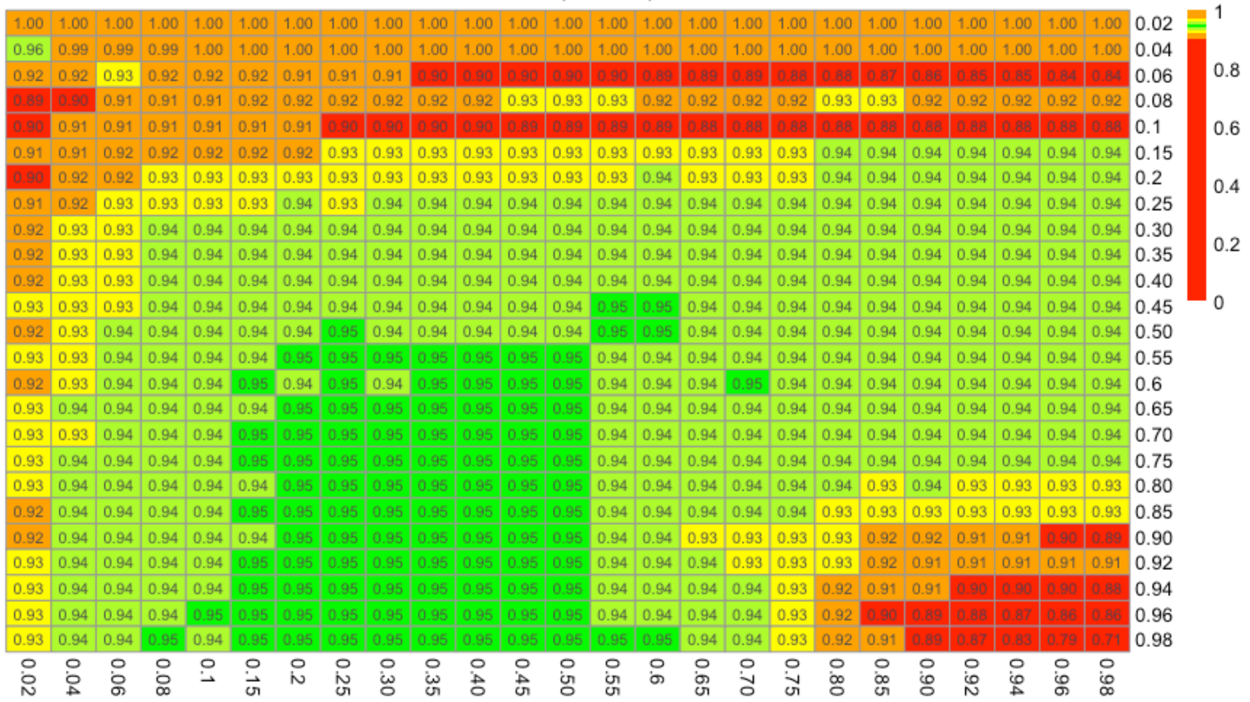

(k) $n_{xrf} = 500$, $n_{yrf} = 500$

	0.02	0.04	0.06	0.08	0.1	0.15	0.2	0.25	0.30	0.35	0.40	0.45	0.50	0.55	0.6	0.65	0.70	0.75	0.80	0.85	0.90	0.92	0.94	0.96	0.98
0.02	0.91	0.92	0.92	0.92	0.92	0.92	0.92	0.93	0.93	0.92	0.93	0.93	0.93	0.93	0.93	0.92	0.92	0.93	0.93	0.93	0.93	0.93	0.93	0.93	0.93
0.04	0.91	0.93	0.93	0.93	0.94	0.93	0.93	0.94	0.94	0.94	0.94	0.94	0.94	0.94	0.94	0.94	0.94	0.94	0.94	0.94	0.94	0.94	0.93	0.94	0.94
0.06	0.92	0.93	0.93	0.94	0.94	0.94	0.94	0.94	0.94	0.94	0.94	0.94	0.94	0.94	0.94	0.94	0.94	0.94	0.94	0.94	0.94	0.94	0.94	0.94	0.94
0.08	0.92	0.93	0.93	0.93	0.94	0.94	0.94	0.94	0.94	0.94	0.94	0.94	0.94	0.94	0.94	0.94	0.94	0.94	0.94	0.94	0.94	0.94	0.94	0.94	0.94
0.1	0.92	0.93	0.94	0.94	0.94	0.94	0.95	0.95	0.95	0.94	0.94	0.94	0.94	0.94	0.94	0.94	0.94	0.94	0.95	0.94	0.94	0.94	0.95	0.94	0.94
0.15	0.92	0.93	0.94	0.94	0.95	0.95	0.94	0.95	0.95	0.95	0.95	0.95	0.95	0.95	0.95	0.95	0.95	0.95	0.95	0.95	0.95	0.95	0.95	0.95	0.95
0.2	0.93	0.94	0.94	0.95	0.95	0.94	0.94	0.95	0.95	0.95	0.95	0.95	0.95	0.94	0.94	0.94	0.94	0.95	0.95	0.95	0.95	0.95	0.95	0.95	0.95
0.25	0.93	0.94	0.94	0.94	0.95	0.95	0.94	0.95	0.95	0.95	0.95	0.95	0.95	0.95	0.95	0.95	0.95	0.95	0.95	0.95	0.95	0.95	0.95	0.95	0.95
0.30	0.93	0.94	0.94	0.94	0.95	0.95	0.94	0.95	0.95	0.95	0.95	0.95	0.95	0.95	0.95	0.95	0.95	0.95	0.95	0.95	0.95	0.95	0.95	0.95	0.95
0.35	0.93	0.94	0.94	0.95	0.95	0.94	0.94	0.95	0.95	0.95	0.95	0.95	0.94	0.95	0.95	0.95	0.95	0.95	0.95	0.95	0.95	0.95	0.95	0.95	0.95
0.40	0.93	0.94	0.94	0.95	0.95	0.95	0.94	0.95	0.95	0.95	0.94	0.95	0.95	0.95	0.95	0.95	0.95	0.95	0.95	0.95	0.95	0.95	0.95	0.95	0.95
0.45	0.93	0.94	0.94	0.95	0.95	0.95	0.94	0.95	0.95	0.95	0.95	0.95	0.95	0.95	0.96	0.95	0.95	0.95	0.95	0.95	0.95	0.95	0.95	0.95	0.95
0.50	0.93	0.94	0.94	0.95	0.95	0.95	0.95	0.95	0.95	0.95	0.95	0.95	0.95	0.96	0.96	0.95	0.95	0.95	0.95	0.95	0.95	0.95	0.95	0.95	0.95
0.55	0.93	0.94	0.94	0.94	0.94	0.95	0.95	0.95	0.95	0.95	0.96	0.96	0.96	0.95	0.95	0.95	0.95	0.95	0.95	0.95	0.95	0.95	0.95	0.95	0.95
0.6	0.93	0.94	0.94	0.94	0.94	0.95	0.95	0.95	0.95	0.95	0.96	0.95	0.96	0.95	0.94	0.95	0.95	0.95	0.95	0.95	0.95	0.95	0.95	0.95	0.95
0.65	0.93	0.94	0.94	0.94	0.94	0.95	0.95	0.95	0.95	0.95	0.95	0.95	0.96	0.94	0.95	0.95	0.95	0.95	0.95	0.95	0.95	0.95	0.95	0.95	0.95
0.70	0.93	0.94	0.94	0.94	0.94	0.95	0.95	0.95	0.95	0.95	0.95	0.95	0.95	0.95	0.95	0.95	0.95	0.95	0.95	0.95	0.95	0.95	0.95	0.95	0.95
0.75	0.93	0.94	0.94	0.94	0.94	0.95	0.95	0.95	0.95	0.95	0.95	0.95	0.95	0.95	0.95	0.95	0.95	0.95	0.95	0.95	0.95	0.95	0.95	0.95	0.95
0.80	0.93	0.94	0.94	0.94	0.94	0.95	0.95	0.95	0.95	0.95	0.95	0.95	0.95	0.95	0.95	0.95	0.95	0.95	0.95	0.95	0.95	0.95	0.95	0.95	0.95
0.85	0.93	0.94	0.94	0.94	0.94	0.95	0.95	0.95	0.95	0.95	0.95	0.95	0.95	0.95	0.95	0.95	0.95	0.95	0.95	0.95	0.95	0.95	0.95	0.95	0.95
0.90	0.93	0.94	0.94	0.94	0.95	0.95	0.95	0.95	0.95	0.95	0.95	0.95	0.95	0.95	0.95	0.95	0.95	0.95	0.95	0.95	0.95	0.95	0.95	0.95	0.95
0.92	0.93	0.94	0.94	0.95	0.95	0.95	0.95	0.95	0.95	0.95	0.95	0.95	0.95	0.95	0.95	0.95	0.95	0.95	0.95	0.95	0.95	0.95	0.95	0.95	0.94
0.94	0.93	0.94	0.94	0.95	0.95	0.95	0.95	0.95	0.95	0.95	0.95	0.95	0.95	0.95	0.95	0.95	0.95	0.95	0.95	0.95	0.95	0.95	0.95	0.95	0.95
0.96	0.93	0.94	0.94	0.95	0.95	0.95	0.95	0.95	0.95	0.95	0.95	0.95	0.95	0.95	0.95	0.95	0.95	0.95	0.95	0.94	0.95	0.95	0.94	0.94	0.94
0.98	0.93	0.94	0.94	0.95	0.95	0.95	0.95	0.95	0.95	0.95	0.95	0.95	0.95	0.95	0.95	0.95	0.95	0.95	0.95	0.94	0.95	0.95	0.94	0.94	0.95

0.8 0.6 0.4 0.2 0

Appendix S4 Figure 2: Estimated 95% coverage probabilities for the confidence intervals constructed using the delta method.

(a) n_{xrf} = 50, n_{yrf} = 50

0.02	0.04	0.06	0.08	0.1	0.15	0.2	0.25	0.30	0.35	0.40	0.45	0.50	0.55	0.6	0.65	0.70	0.75	0.80	0.85	0.90	0.92	0.94	0.96	0.98	
0.91	0.95	0.96	0.96	0.96	0.95	0.95	0.94	0.94	0.94	0.94	0.94	0.94	0.94	0.94	0.94	0.94	0.94	0.94	0.94	0.95	0.95	0.96	0.97	0.97	0.02
0.96	0.97	0.98	0.97	0.97	0.97	0.97	0.97	0.96	0.96	0.96	0.96	0.96	0.96	0.96	0.96	0.96	0.95	0.95	0.95	0.95	0.95	0.95	0.95	0.95	0.04
0.96	0.97	0.97	0.97	0.97	0.97	0.97	0.97	0.97	0.97	0.96	0.97	0.97	0.97	0.97	0.96	0.96	0.97	0.96	0.97	0.97	0.97	0.97	0.97	0.97	0.06
0.96	0.97	0.98	0.97	0.97	0.97	0.97	0.97	0.97	0.97	0.97	0.97	0.97	0.97	0.97	0.97	0.97	0.97	0.97	0.97	0.97	0.96	0.96	0.96	0.96	0.08
0.96	0.97	0.97	0.97	0.96	0.96	0.96	0.96	0.97	0.97	0.97	0.97	0.97	0.97	0.97	0.97	0.97	0.97	0.97	0.97	0.97	0.97	0.97	0.98	0.98	0.1
0.95	0.97	0.97	0.97	0.96	0.95	0.96	0.95	0.96	0.96	0.96	0.96	0.96	0.96	0.96	0.96	0.96	0.96	0.96	0.96	0.96	0.96	0.96	0.96	0.96	0.15
0.95	0.96	0.97	0.97	0.96	0.96	0.95	0.95	0.95	0.95	0.95	0.95	0.95	0.96	0.96	0.96	0.96	0.96	0.96	0.96	0.96	0.95	0.96	0.95	0.96	0.2
0.94	0.97	0.97	0.97	0.96	0.95	0.95	0.95	0.95	0.95	0.95	0.95	0.95	0.96	0.96	0.96	0.96	0.96	0.96	0.95	0.96	0.96	0.96	0.95	0.95	0.25
0.94	0.96	0.97	0.97	0.97	0.96	0.95	0.95	0.95	0.95	0.95	0.95	0.95	0.95	0.96	0.95	0.96	0.96	0.95	0.95	0.96	0.96	0.96	0.96	0.96	0.30
0.94	0.96	0.97	0.97	0.97	0.96	0.95	0.95	0.95	0.95	0.95	0.95	0.95	0.95	0.95	0.95	0.96	0.95	0.95	0.95	0.95	0.95	0.95	0.96	0.96	0.35
0.94	0.96	0.96	0.97	0.97	0.96	0.95	0.95	0.95	0.95	0.95	0.95	0.95	0.95	0.95	0.95	0.95	0.95	0.96	0.95	0.96	0.96	0.96	0.95	0.95	0.40
0.94	0.96	0.97	0.97	0.97	0.96	0.95	0.95	0.95	0.96	0.95	0.95	0.95	0.95	0.95	0.95	0.95	0.95	0.95	0.95	0.95	0.95	0.96	0.96	0.96	0.45
0.94	0.96	0.97	0.97	0.97	0.96	0.95	0.95	0.95	0.95	0.95	0.95	0.95	0.95	0.95	0.95	0.95	0.95	0.95	0.95	0.95	0.95	0.96	0.96	0.96	0.50
0.94	0.96	0.97	0.97	0.97	0.96	0.96	0.96	0.95	0.95	0.95	0.95	0.95	0.95	0.95	0.95	0.95	0.95	0.95	0.95	0.95	0.95	0.96	0.96	0.96	0.55
0.94	0.96	0.97	0.97	0.97	0.96	0.96	0.95	0.96	0.96	0.95	0.95	0.95	0.95	0.95	0.95	0.95	0.95	0.95	0.95	0.95	0.95	0.95	0.96	0.96	0.6
0.94	0.96	0.96	0.97	0.97	0.96	0.96	0.95	0.95	0.95	0.95	0.95	0.95	0.95	0.95	0.95	0.95	0.95	0.95	0.95	0.95	0.95	0.95	0.96	0.96	0.65
0.94	0.96	0.97	0.97	0.97	0.96	0.95	0.95	0.96	0.96	0.95	0.95	0.95	0.95	0.95	0.95	0.95	0.95	0.95	0.95	0.95	0.96	0.96	0.95	0.95	0.70
0.94	0.95	0.97	0.97	0.97	0.96	0.96	0.95	0.95	0.95	0.95	0.95	0.95	0.95	0.95	0.95	0.96	0.95	0.95	0.95	0.95	0.95	0.96	0.96	0.95	0.75
0.94	0.96	0.97	0.97	0.97	0.96	0.96	0.95	0.95	0.95	0.95	0.95	0.95	0.95	0.95	0.95	0.95	0.95	0.95	0.95	0.95	0.95	0.96	0.96	0.95	0.80
0.94	0.95	0.97	0.97	0.97	0.96	0.96	0.95	0.95	0.95	0.95	0.95	0.95	0.95	0.95	0.95	0.95	0.95	0.95	0.96	0.96	0.95	0.96	0.96	0.95	0.85
0.95	0.95	0.97	0.97	0.97	0.96	0.96	0.96	0.96	0.95	0.96	0.95	0.95	0.95	0.95	0.95	0.96	0.95	0.96	0.96	0.96	0.95	0.97	0.97	0.95	0.90
0.95	0.95	0.97	0.96	0.97	0.96	0.96	0.96	0.95	0.95	0.96	0.95	0.95	0.95	0.95	0.96	0.96	0.95	0.95	0.95	0.95	0.96	0.98	0.95	0.96	0.92
0.96	0.95	0.97	0.96	0.97	0.96	0.96	0.96	0.96	0.95	0.96	0.95	0.96	0.96	0.95	0.95	0.96	0.96	0.96	0.96	0.97	0.97	0.96	0.97	0.93	0.94
0.97	0.94	0.97	0.96	0.97	0.96	0.95	0.95	0.96	0.96	0.95	0.96	0.96	0.96	0.96	0.96	0.95	0.96	0.96	0.97	0.97	0.96	0.97	0.94	0.95	0.96
0.97	0.94	0.97	0.96	0.97	0.95	0.95	0.95	0.96	0.96	0.95	0.95	0.96	0.95	0.95	0.96	0.95	0.95	0.95	0.95	0.95	0.96	0.93	0.94	0.88	0.98

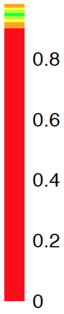

(b) n_{xrf} = 100, n_{yrf} = 50

0.02	0.04	0.06	0.08	0.1	0.15	0.2	0.25	0.30	0.35	0.40	0.45	0.50	0.55	0.6	0.65	0.70	0.75	0.80	0.85	0.90	0.92	0.94	0.96	0.98	
0.95	0.96	0.96	0.95	0.95	0.94	0.94	0.94	0.94	0.94	0.94	0.94	0.94	0.94	0.94	0.94	0.94	0.94	0.95	0.95	0.95	0.95	0.96	0.96	0.97	0.02
0.97	0.97	0.97	0.97	0.96	0.96	0.97	0.96	0.96	0.96	0.96	0.96	0.96	0.96	0.96	0.95	0.96	0.95	0.95	0.95	0.95	0.95	0.95	0.94	0.95	0.04
0.97	0.97	0.97	0.97	0.97	0.97	0.97	0.97	0.97	0.97	0.97	0.97	0.96	0.97	0.97	0.97	0.97	0.97	0.97	0.97	0.97	0.97	0.97	0.97	0.97	0.06
0.97	0.97	0.96	0.97	0.97	0.97	0.97	0.97	0.97	0.97	0.97	0.97	0.97	0.97	0.97	0.97	0.97	0.97	0.97	0.96	0.96	0.96	0.96	0.96	0.96	0.08
0.97	0.97	0.97	0.97	0.96	0.97	0.97	0.97	0.97	0.97	0.97	0.97	0.97	0.97	0.97	0.97	0.97	0.97	0.97	0.97	0.97	0.97	0.97	0.97	0.98	0.1
0.97	0.97	0.96	0.95	0.96	0.96	0.96	0.96	0.96	0.96	0.96	0.96	0.96	0.96	0.96	0.96	0.96	0.96	0.96	0.96	0.96	0.96	0.96	0.96	0.96	0.15
0.96	0.97	0.96	0.96	0.95	0.95	0.95	0.95	0.96	0.96	0.96	0.96	0.96	0.95	0.96	0.96	0.96	0.96	0.96	0.96	0.96	0.96	0.96	0.95	0.95	0.2
0.96	0.97	0.96	0.95	0.95	0.95	0.95	0.95	0.95	0.96	0.96	0.96	0.96	0.95	0.95	0.96	0.96	0.96	0.96	0.95	0.95	0.95	0.95	0.95	0.95	0.25
0.96	0.97	0.96	0.96	0.95	0.95	0.95	0.95	0.95	0.95	0.96	0.96	0.96	0.95	0.95	0.95	0.96	0.95	0.95	0.95	0.95	0.95	0.96	0.96	0.96	0.30
0.96	0.97	0.96	0.95	0.95	0.95	0.95	0.95	0.95	0.95	0.96	0.95	0.96	0.95	0.95	0.95	0.95	0.95	0.95	0.95	0.95	0.95	0.95	0.95	0.96	0.35
0.96	0.97	0.97	0.96	0.95	0.95	0.95	0.95	0.95	0.95	0.96	0.96	0.96	0.95	0.95	0.95	0.95	0.95	0.95	0.95	0.95	0.95	0.96	0.95	0.95	0.40
0.96	0.97	0.96	0.96	0.96	0.95	0.95	0.95	0.95	0.95	0.96	0.95	0.96	0.95	0.95	0.95	0.95	0.95	0.95	0.95	0.95	0.95	0.95	0.95	0.95	0.45
0.96	0.97	0.97	0.96	0.95	0.95	0.96	0.95	0.95	0.96	0.96	0.95	0.96	0.95	0.95	0.95	0.95	0.95	0.95	0.95	0.95	0.95	0.95	0.95	0.96	0.50
0.96	0.97	0.97	0.96	0.96	0.95	0.95	0.95	0.95	0.95	0.95	0.95	0.95	0.95	0.95	0.95	0.95	0.95	0.95	0.95	0.95	0.95	0.95	0.95	0.96	0.55
0.96	0.97	0.97	0.96	0.96	0.96	0.95	0.95	0.95	0.95	0.95	0.95	0.95	0.95	0.96	0.95	0.95	0.95	0.95	0.95	0.95	0.95	0.95	0.95	0.95	0.6
0.96	0.97	0.97	0.96	0.96	0.95	0.95	0.95	0.95	0.95	0.95	0.95	0.95	0.95	0.95	0.95	0.95	0.95	0.95	0.95	0.95	0.95	0.95	0.95	0.95	0.65
0.96	0.97	0.97	0.96	0.96	0.95	0.95	0.95	0.95	0.95	0.95	0.95	0.95	0.95	0.96	0.95	0.95	0.95	0.95	0.95	0.95	0.95	0.95	0.95	0.96	0.70
0.96	0.97	0.97	0.96	0.96	0.95	0.95	0.95	0.95	0.95	0.95	0.95	0.95	0.95	0.95	0.95	0.95	0.95	0.95	0.95	0.95	0.95	0.95	0.95	0.95	0.75
0.95	0.96	0.97	0.96	0.96	0.96	0.95	0.95	0.95	0.95	0.95	0.95	0.95	0.95	0.95	0.95	0.95	0.95	0.95	0.95	0.95	0.95	0.96	0.96	0.95	0.80
0.95	0.97	0.97	0.96	0.95	0.96	0.95	0.95	0.95	0.95	0.95	0.95	0.95	0.95	0.95	0.95	0.95	0.95	0.95	0.95	0.95	0.95	0.95	0.95	0.96	0.85
0.95	0.96	0.97	0.96	0.96	0.96	0.96	0.95	0.95	0.95	0.95	0.95	0.95	0.95	0.95	0.96	0.95	0.95	0.95	0.95	0.95	0.95	0.95	0.96	0.97	0.90
0.95	0.96	0.97	0.96	0.96	0.95	0.96	0.95	0.95	0.95	0.95	0.95	0.95	0.96	0.95	0.96	0.95	0.95	0.95	0.96	0.95	0.96	0.96	0.96	0.97	0.92
0.94	0.96	0.97	0.96	0.96	0.95	0.96	0.95	0.95	0.95	0.96	0.95	0.96	0.95	0.96	0.96	0.95	0.96	0.96	0.96	0.96	0.96	0.97	0.97	0.96	0.94
0.94	0.96	0.96	0.96	0.96	0.95	0.96	0.96	0.95	0.95	0.95	0.96	0.96	0.96	0.96	0.95	0.96	0.96	0.96	0.96	0.96	0.97	0.97	0.96	0.96	0.96
0.94	0.96	0.96	0.96	0.95	0.96	0.95	0.95	0.96	0.95	0.95	0.96	0.96	0.96	0.95	0.95	0.96	0.95	0.95	0.96	0.96	0.96	0.95	0.95	0.94	0.98

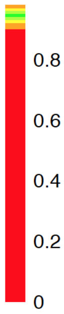

(c) $n_{xrf} = 100$, $n_{yrf} = 100$

0.02	0.04	0.06	0.08	0.1	0.15	0.2	0.25	0.30	0.35	0.40	0.45	0.50	0.55	0.6	0.65	0.70	0.75	0.80	0.85	0.90	0.92	0.94	0.96	0.98	
0.96	0.97	0.97	0.97	0.97	0.96	0.96	0.96	0.96	0.96	0.96	0.96	0.96	0.96	0.95	0.96	0.96	0.95	0.95	0.95	0.94	0.94	0.94	0.94	0.94	0.02
0.97	0.97	0.96	0.97	0.97	0.97	0.97	0.97	0.97	0.97	0.97	0.97	0.97	0.97	0.97	0.97	0.97	0.96	0.96	0.96	0.96	0.96	0.96	0.96	0.96	0.04
0.97	0.96	0.96	0.96	0.96	0.96	0.96	0.97	0.97	0.97	0.97	0.97	0.97	0.97	0.97	0.97	0.97	0.97	0.97	0.97	0.97	0.97	0.97	0.96	0.96	0.06
0.97	0.97	0.96	0.95	0.96	0.96	0.96	0.96	0.96	0.96	0.96	0.96	0.96	0.96	0.96	0.96	0.96	0.96	0.96	0.96	0.96	0.96	0.96	0.96	0.96	0.08
0.97	0.97	0.96	0.96	0.96	0.95	0.95	0.95	0.95	0.96	0.96	0.96	0.96	0.96	0.96	0.96	0.96	0.96	0.96	0.96	0.96	0.96	0.95	0.96	0.96	0.1
0.96	0.97	0.96	0.95	0.95	0.95	0.95	0.95	0.95	0.95	0.96	0.96	0.96	0.95	0.95	0.95	0.96	0.96	0.95	0.95	0.95	0.95	0.95	0.96	0.96	0.15
0.96	0.97	0.96	0.96	0.95	0.95	0.95	0.95	0.95	0.95	0.96	0.96	0.96	0.95	0.95	0.95	0.96	0.95	0.95	0.95	0.95	0.95	0.95	0.95	0.95	0.2
0.96	0.97	0.97	0.96	0.96	0.95	0.95	0.95	0.95	0.95	0.96	0.95	0.96	0.95	0.95	0.95	0.95	0.95	0.95	0.95	0.95	0.95	0.95	0.95	0.95	0.25
0.96	0.97	0.96	0.96	0.96	0.95	0.95	0.95	0.95	0.95	0.95	0.95	0.95	0.95	0.95	0.95	0.95	0.95	0.95	0.95	0.95	0.95	0.95	0.95	0.96	0.30
0.96	0.97	0.96	0.96	0.95	0.95	0.95	0.95	0.95	0.95	0.95	0.95	0.95	0.95	0.95	0.95	0.95	0.95	0.95	0.95	0.95	0.95	0.95	0.95	0.95	0.35
0.96	0.97	0.97	0.96	0.95	0.95	0.95	0.95	0.95	0.95	0.95	0.95	0.95	0.95	0.95	0.95	0.95	0.95	0.95	0.95	0.95	0.95	0.95	0.95	0.95	0.40
0.96	0.97	0.96	0.96	0.96	0.95	0.95	0.95	0.95	0.95	0.95	0.95	0.96	0.95	0.96	0.95	0.95	0.95	0.96	0.95	0.95	0.95	0.95	0.95	0.95	0.45
0.96	0.97	0.97	0.96	0.96	0.95	0.95	0.95	0.95	0.95	0.96	0.96	0.96	0.95	0.95	0.95	0.95	0.95	0.96	0.96	0.95	0.95	0.95	0.95	0.95	0.50
0.96	0.97	0.97	0.96	0.96	0.96	0.95	0.95	0.95	0.95	0.95	0.95	0.95	0.96	0.95	0.95	0.96	0.95	0.95	0.95	0.95	0.95	0.95	0.95	0.95	0.55
0.95	0.97	0.97	0.96	0.96	0.96	0.95	0.95	0.95	0.95	0.95	0.95	0.95	0.95	0.95	0.95	0.95	0.95	0.95	0.95	0.95	0.95	0.95	0.95	0.95	0.6
0.95	0.97	0.97	0.96	0.96	0.95	0.95	0.95	0.95	0.95	0.95	0.95	0.95	0.95	0.95	0.96	0.95	0.95	0.95	0.95	0.95	0.95	0.95	0.95	0.95	0.65
0.95	0.97	0.97	0.96	0.96	0.95	0.95	0.95	0.95	0.95	0.95	0.95	0.95	0.95	0.95	0.95	0.95	0.95	0.95	0.95	0.95	0.95	0.95	0.95	0.96	0.70
0.95	0.96	0.97	0.96	0.96	0.95	0.95	0.95	0.95	0.95	0.95	0.95	0.95	0.95	0.95	0.96	0.95	0.95	0.95	0.95	0.95	0.95	0.95	0.95	0.96	0.75
0.95	0.96	0.97	0.96	0.96	0.95	0.95	0.95	0.95	0.95	0.95	0.95	0.95	0.95	0.95	0.95	0.95	0.95	0.96	0.95	0.95	0.95	0.95	0.95	0.95	0.80
0.95	0.96	0.97	0.96	0.96	0.95	0.95	0.95	0.95	0.95	0.95	0.95	0.95	0.95	0.95	0.95	0.95	0.95	0.95	0.95	0.96	0.95	0.95	0.95	0.95	0.85
0.94	0.96	0.97	0.96	0.96	0.95	0.95	0.95	0.95	0.95	0.95	0.95	0.95	0.95	0.95	0.95	0.95	0.95	0.95	0.96	0.96	0.95	0.95	0.96	0.95	0.90
0.94	0.96	0.97	0.96	0.96	0.95	0.95	0.95	0.95	0.95	0.95	0.95	0.95	0.95	0.95	0.95	0.95	0.95	0.95	0.95	0.95	0.95	0.96	0.95	0.96	0.92
0.94	0.96	0.97	0.96	0.96	0.95	0.95	0.95	0.95	0.95	0.95	0.95	0.95	0.95	0.96	0.95	0.95	0.95	0.95	0.95	0.95	0.96	0.95	0.97	0.96	0.94
0.94	0.96	0.96	0.96	0.96	0.95	0.95	0.95	0.95	0.95	0.95	0.95	0.96	0.95	0.95	0.95	0.95	0.95	0.95	0.95	0.96	0.95	0.97	0.96	0.95	0.96
0.94	0.96	0.96	0.96	0.96	0.96	0.95	0.95	0.95	0.95	0.95	0.95	0.95	0.95	0.96	0.95	0.95	0.96	0.96	0.95	0.96	0.96	0.96	0.95	0.93	0.98

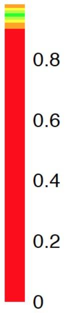

(d) $n_{xrf} = 200$, $n_{yrf} = 50$

0.02	0.04	0.06	0.08	0.1	0.15	0.2	0.25	0.30	0.35	0.40	0.45	0.50	0.55	0.6	0.65	0.70	0.75	0.80	0.85	0.90	0.92	0.94	0.96	0.98	
0.96	0.95	0.95	0.94	0.94	0.94	0.94	0.94	0.94	0.94	0.94	0.94	0.94	0.94	0.94	0.94	0.95	0.94	0.95	0.96	0.95	0.96	0.96	0.97	0.97	0.02
0.97	0.97	0.97	0.96	0.96	0.96	0.96	0.96	0.96	0.96	0.96	0.96	0.95	0.95	0.95	0.95	0.95	0.95	0.95	0.95	0.94	0.94	0.94	0.94	0.94	0.04
0.97	0.97	0.97	0.97	0.97	0.97	0.97	0.96	0.96	0.97	0.97	0.97	0.97	0.97	0.97	0.97	0.97	0.97	0.97	0.97	0.97	0.97	0.97	0.97	0.97	0.06
0.97	0.97	0.97	0.97	0.97	0.97	0.97	0.97	0.97	0.97	0.97	0.97	0.97	0.97	0.97	0.97	0.96	0.96	0.96	0.96	0.96	0.96	0.96	0.96	0.96	0.08
0.97	0.96	0.96	0.97	0.97	0.97	0.97	0.97	0.97	0.97	0.97	0.97	0.97	0.97	0.97	0.97	0.97	0.97	0.97	0.97	0.97	0.97	0.97	0.97	0.97	0.1
0.97	0.96	0.96	0.96	0.96	0.96	0.96	0.96	0.96	0.96	0.96	0.96	0.96	0.96	0.96	0.96	0.96	0.96	0.96	0.96	0.96	0.96	0.96	0.96	0.96	0.15
0.97	0.96	0.95	0.95	0.95	0.96	0.96	0.96	0.96	0.96	0.96	0.96	0.96	0.96	0.96	0.96	0.96	0.96	0.96	0.96	0.95	0.95	0.95	0.95	0.95	0.2
0.97	0.96	0.95	0.95	0.95	0.95	0.96	0.96	0.95	0.96	0.96	0.96	0.96	0.96	0.95	0.96	0.95	0.95	0.96	0.95	0.95	0.95	0.95	0.95	0.95	0.25
0.97	0.96	0.95	0.95	0.95	0.95	0.95	0.96	0.95	0.95	0.95	0.95	0.96	0.96	0.95	0.96	0.95	0.95	0.96	0.96	0.95	0.95	0.95	0.96	0.96	0.30
0.97	0.96	0.95	0.95	0.95	0.95	0.95	0.95	0.95	0.95	0.95	0.95	0.95	0.95	0.95	0.95	0.95	0.95	0.95	0.95	0.95	0.95	0.95	0.96	0.96	0.35
0.97	0.96	0.95	0.95	0.95	0.95	0.95	0.96	0.95	0.95	0.96	0.95	0.95	0.96	0.95	0.95	0.95	0.95	0.95	0.95	0.95	0.95	0.95	0.95	0.95	0.40
0.97	0.96	0.95	0.95	0.95	0.95	0.95	0.95	0.95	0.95	0.95	0.95	0.95	0.95	0.95	0.95	0.95	0.95	0.95	0.96	0.95	0.95	0.95	0.95	0.95	0.45
0.97	0.96	0.95	0.95	0.95	0.96	0.95	0.95	0.95	0.95	0.95	0.95	0.95	0.95	0.95	0.95	0.95	0.95	0.95	0.95	0.95	0.95	0.95	0.95	0.95	0.50
0.97	0.96	0.96	0.95	0.95	0.95	0.95	0.95	0.95	0.95	0.95	0.95	0.95	0.95	0.95	0.95	0.95	0.95	0.95	0.95	0.95	0.95	0.95	0.95	0.95	0.55
0.97	0.96	0.95	0.95	0.95	0.95	0.95	0.95	0.95	0.95	0.95	0.95	0.95	0.95	0.95	0.95	0.95	0.95	0.95	0.95	0.95	0.95	0.95	0.95	0.95	0.6
0.97	0.96	0.96	0.95	0.95	0.95	0.95	0.95	0.95	0.95	0.95	0.95	0.95	0.95	0.95	0.95	0.95	0.95	0.95	0.95	0.95	0.95	0.95	0.95	0.96	0.65
0.97	0.96	0.95	0.96	0.95	0.95	0.95	0.95	0.95	0.95	0.95	0.95	0.95	0.95	0.95	0.95	0.95	0.95	0.95	0.95	0.95	0.95	0.95	0.95	0.96	0.70
0.96	0.96	0.95	0.95	0.95	0.95	0.95	0.95	0.95	0.96	0.95	0.95	0.95	0.95	0.95	0.95	0.95	0.95	0.95	0.95	0.95	0.95	0.95	0.95	0.95	0.75
0.97	0.96	0.95	0.95	0.95	0.95	0.95	0.95	0.95	0.95	0.95	0.95	0.95	0.95	0.95	0.95	0.95	0.95	0.95	0.95	0.95	0.95	0.95	0.95	0.95	0.80
0.96	0.96	0.95	0.95	0.95	0.95	0.95	0.95	0.95	0.95	0.95	0.95	0.95	0.94	0.95	0.95	0.95	0.95	0.95	0.95	0.94	0.95	0.95	0.95	0.96	0.85
0.96	0.96	0.96	0.95	0.95	0.95	0.95	0.95	0.95	0.96	0.95	0.96	0.95	0.95	0.95	0.95	0.95	0.95	0.95	0.95	0.95	0.95	0.95	0.95	0.94	0.90
0.96	0.96	0.96	0.95	0.95	0.95	0.95	0.95	0.95	0.95	0.95	0.96	0.95	0.95	0.95	0.95	0.95	0.96	0.96	0.96	0.95	0.95	0.95	0.96	0.97	0.92
0.96	0.96	0.96	0.95	0.95	0.96	0.95	0.95	0.96	0.95	0.96	0.96	0.96	0.95	0.95	0.96	0.95	0.96	0.96	0.97	0.96	0.96	0.97	0.97	0.97	0.94
0.96	0.96	0.96	0.96	0.95	0.96	0.95	0.95	0.95	0.95	0.96	0.96	0.96	0.95	0.96	0.95	0.96	0.96	0.96	0.97	0.97	0.98	0.97	0.97	0.96	0.96
0.96	0.95	0.95	0.95	0.95	0.96	0.96	0.95	0.95	0.95	0.96	0.96	0.96	0.95	0.96	0.96	0.96	0.96	0.96	0.97	0.96	0.96	0.96	0.96	0.95	0.98

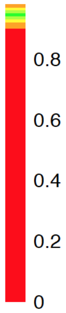

(e) n_{xrf}=200, n_{yrf} = 200

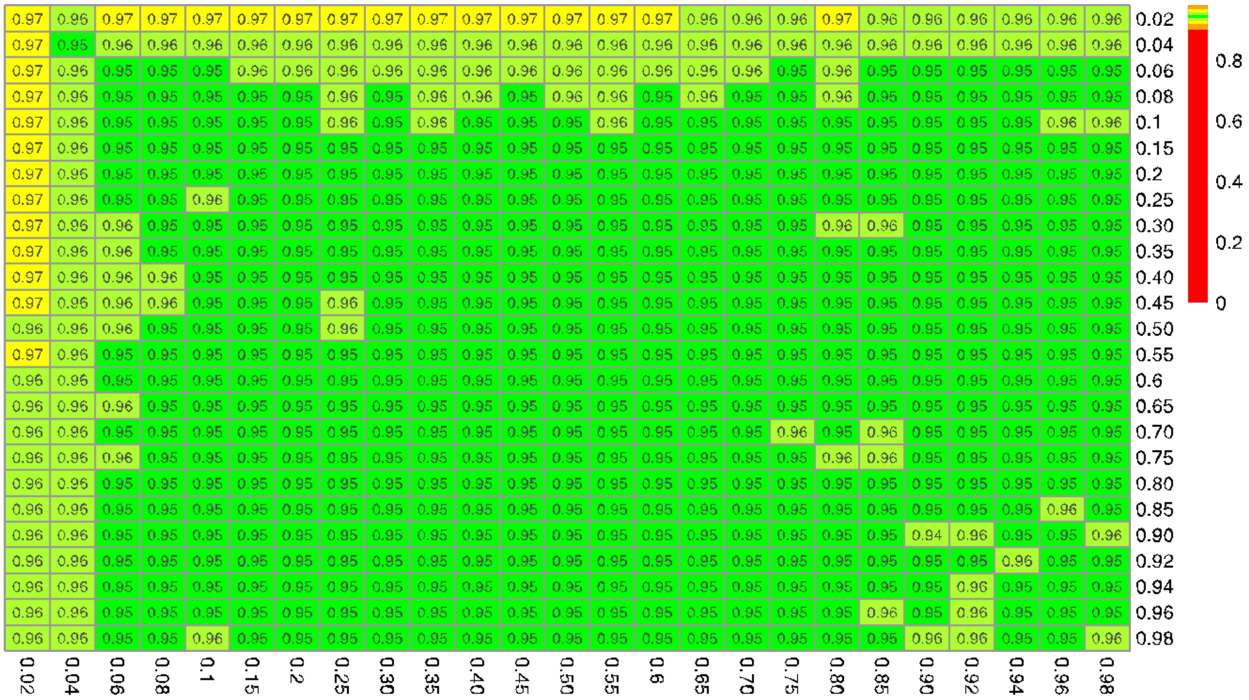

(f) n_{xrf}=300, n_{yrf} = 50

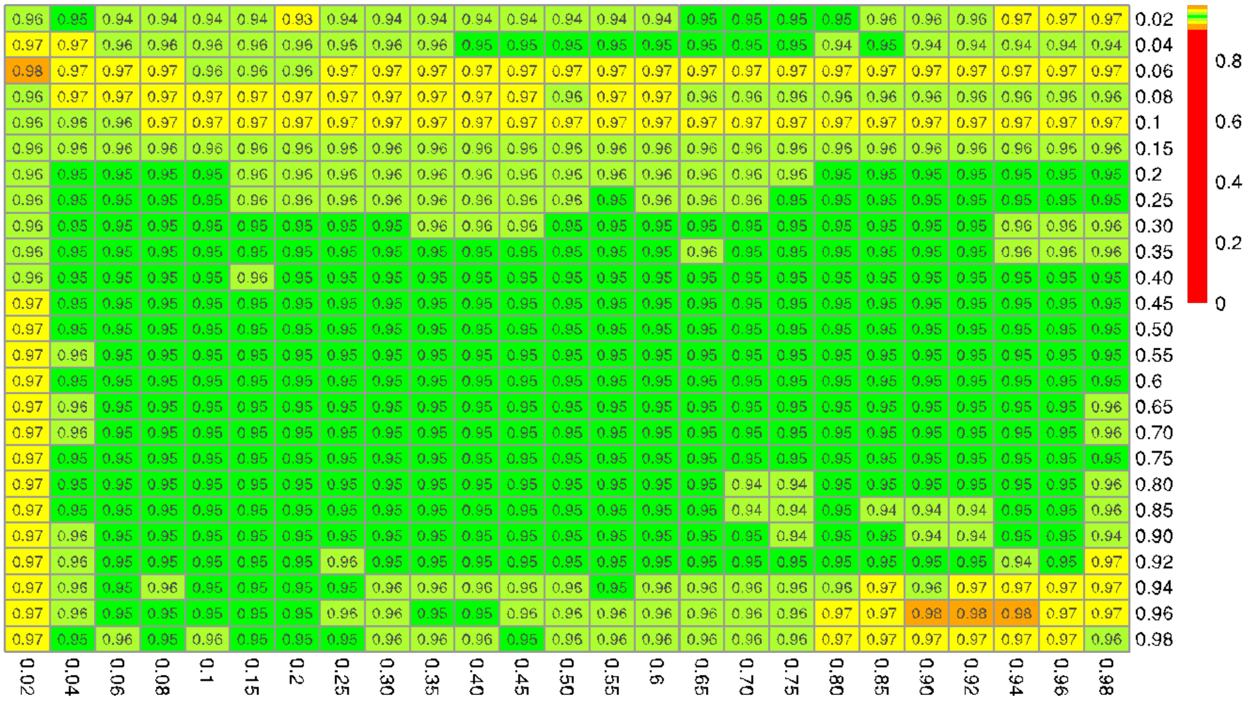

(g) n_{xrf}=300, n_{yrf} = 300

	0.02	0.04	0.06	0.08	0.1	0.15	0.2	0.25	0.30	0.35	0.40	0.45	0.50	0.55	0.6	0.65	0.70	0.75	0.80	0.85	0.90	0.92	0.94	0.96	0.98
0.02	0.96	0.96	0.96	0.97	0.97	0.97	0.97	0.97	0.97	0.97	0.97	0.97	0.97	0.97	0.97	0.97	0.97	0.97	0.97	0.97	0.96	0.96	0.96	0.96	0.96
0.04	0.96	0.95	0.95	0.95	0.95	0.96	0.96	0.96	0.96	0.96	0.96	0.96	0.96	0.96	0.96	0.96	0.96	0.96	0.95	0.95	0.95	0.95	0.95	0.95	0.95
0.06	0.96	0.95	0.95	0.95	0.95	0.96	0.96	0.96	0.95	0.96	0.96	0.96	0.96	0.95	0.96	0.96	0.96	0.96	0.95	0.95	0.95	0.95	0.95	0.96	0.96
0.08	0.97	0.95	0.95	0.95	0.95	0.95	0.95	0.95	0.95	0.95	0.95	0.95	0.95	0.95	0.95	0.95	0.95	0.95	0.95	0.95	0.95	0.95	0.95	0.95	0.96
0.1	0.97	0.95	0.95	0.95	0.95	0.95	0.95	0.95	0.95	0.95	0.95	0.95	0.95	0.95	0.95	0.95	0.95	0.95	0.95	0.95	0.95	0.95	0.95	0.95	0.96
0.15	0.97	0.96	0.95	0.95	0.95	0.95	0.95	0.95	0.95	0.95	0.95	0.95	0.95	0.95	0.95	0.95	0.95	0.95	0.95	0.95	0.95	0.95	0.95	0.95	0.95
0.2	0.97	0.96	0.95	0.95	0.95	0.96	0.95	0.95	0.95	0.95	0.95	0.95	0.95	0.95	0.95	0.95	0.95	0.95	0.95	0.95	0.95	0.95	0.95	0.95	0.95
0.25	0.97	0.96	0.95	0.96	0.95	0.95	0.95	0.95	0.95	0.95	0.95	0.95	0.95	0.95	0.95	0.95	0.95	0.95	0.95	0.95	0.95	0.95	0.95	0.95	0.95
0.30	0.97	0.96	0.95	0.96	0.95	0.95	0.95	0.95	0.95	0.95	0.95	0.95	0.95	0.95	0.95	0.95	0.95	0.95	0.95	0.95	0.95	0.95	0.95	0.95	0.95
0.35	0.97	0.96	0.95	0.95	0.95	0.96	0.96	0.95	0.95	0.95	0.95	0.95	0.95	0.95	0.95	0.95	0.95	0.95	0.95	0.95	0.95	0.95	0.95	0.95	0.95
0.40	0.97	0.96	0.95	0.96	0.95	0.96	0.95	0.95	0.95	0.95	0.95	0.95	0.95	0.95	0.95	0.95	0.95	0.95	0.95	0.95	0.95	0.95	0.95	0.95	0.95
0.45	0.97	0.96	0.95	0.96	0.95	0.96	0.96	0.95	0.95	0.95	0.95	0.95	0.95	0.95	0.95	0.95	0.95	0.95	0.95	0.95	0.95	0.95	0.95	0.95	0.95
0.50	0.97	0.96	0.95	0.95	0.95	0.95	0.96	0.95	0.95	0.95	0.95	0.95	0.95	0.95	0.95	0.95	0.95	0.95	0.95	0.95	0.95	0.95	0.95	0.95	0.95
0.55	0.97	0.96	0.95	0.95	0.95	0.95	0.95	0.95	0.95	0.95	0.95	0.95	0.95	0.95	0.95	0.95	0.95	0.95	0.95	0.95	0.95	0.95	0.95	0.95	0.95
0.6	0.97	0.96	0.95	0.95	0.95	0.95	0.95	0.95	0.95	0.95	0.95	0.95	0.95	0.95	0.95	0.95	0.95	0.95	0.95	0.95	0.95	0.95	0.95	0.95	0.95
0.65	0.97	0.96	0.95	0.95	0.95	0.95	0.95	0.95	0.95	0.95	0.95	0.95	0.95	0.95	0.95	0.95	0.95	0.95	0.95	0.95	0.95	0.95	0.95	0.95	0.95
0.70	0.97	0.96	0.95	0.95	0.95	0.95	0.95	0.95	0.95	0.95	0.95	0.95	0.95	0.95	0.95	0.95	0.95	0.95	0.95	0.95	0.95	0.95	0.95	0.95	0.95
0.75	0.96	0.96	0.95	0.95	0.95	0.95	0.95	0.95	0.95	0.95	0.95	0.95	0.95	0.95	0.95	0.95	0.95	0.95	0.95	0.95	0.95	0.95	0.95	0.95	0.95
0.80	0.96	0.96	0.95	0.95	0.95	0.95	0.95	0.95	0.95	0.95	0.95	0.95	0.95	0.95	0.95	0.95	0.95	0.95	0.96	0.95	0.95	0.95	0.95	0.95	0.95
0.85	0.96	0.95	0.95	0.95	0.95	0.95	0.95	0.95	0.95	0.95	0.95	0.95	0.95	0.95	0.95	0.95	0.95	0.95	0.95	0.95	0.95	0.95	0.95	0.95	0.95
0.90	0.96	0.95	0.95	0.95	0.95	0.95	0.95	0.95	0.95	0.95	0.95	0.95	0.95	0.95	0.95	0.95	0.95	0.95	0.95	0.95	0.95	0.95	0.95	0.95	0.95
0.92	0.96	0.95	0.95	0.95	0.95	0.95	0.95	0.95	0.95	0.95	0.95	0.95	0.95	0.95	0.95	0.95	0.95	0.95	0.95	0.95	0.95	0.96	0.95	0.95	0.95
0.94	0.96	0.95	0.95	0.95	0.96	0.95	0.95	0.95	0.95	0.95	0.95	0.95	0.95	0.95	0.95	0.95	0.95	0.95	0.95	0.95	0.95	0.95	0.95	0.95	0.95
0.96	0.96	0.95	0.95	0.95	0.96	0.95	0.95	0.95	0.95	0.95	0.95	0.95	0.95	0.95	0.95	0.95	0.95	0.95	0.95	0.95	0.96	0.95	0.95	0.95	0.96
0.98	0.96	0.95	0.96	0.95	0.96	0.95	0.95	0.95	0.95	0.95	0.95	0.95	0.95	0.95	0.95	0.95	0.95	0.95	0.95	0.95	0.95	0.95	0.95	0.96	0.95

(h) n_{xrf}=400, n_{yrf} = 50

	0.02	0.04	0.06	0.08	0.1	0.15	0.2	0.25	0.30	0.35	0.40	0.45	0.50	0.55	0.6	0.65	0.70	0.75	0.80	0.85	0.90	0.92	0.94	0.96	0.98
0.02	0.95	0.94	0.94	0.94	0.94	0.94	0.94	0.93	0.94	0.94	0.94	0.94	0.94	0.94	0.95	0.95	0.95	0.95	0.96	0.96	0.97	0.97	0.97	0.97	0.97
0.04	0.97	0.97	0.96	0.96	0.96	0.96	0.96	0.96	0.95	0.95	0.95	0.95	0.95	0.95	0.95	0.95	0.95	0.95	0.95	0.94	0.94	0.95	0.94	0.94	0.94
0.06	0.97	0.97	0.96	0.97	0.96	0.96	0.97	0.97	0.97	0.97	0.97	0.97	0.97	0.97	0.97	0.97	0.97	0.97	0.97	0.97	0.97	0.97	0.97	0.97	0.97
0.08	0.97	0.97	0.97	0.97	0.97	0.97	0.97	0.97	0.97	0.97	0.96	0.96	0.96	0.96	0.96	0.96	0.96	0.96	0.96	0.96	0.96	0.96	0.96	0.96	0.96
0.1	0.96	0.97	0.97	0.97	0.97	0.97	0.97	0.97	0.97	0.97	0.97	0.97	0.97	0.97	0.97	0.97	0.97	0.97	0.97	0.97	0.98	0.98	0.97	0.97	0.97
0.15	0.95	0.96	0.96	0.96	0.96	0.96	0.96	0.96	0.96	0.96	0.96	0.96	0.96	0.96	0.96	0.96	0.96	0.96	0.96	0.96	0.96	0.96	0.96	0.96	0.96
0.2	0.95	0.95	0.95	0.95	0.96	0.96	0.96	0.96	0.96	0.96	0.96	0.96	0.96	0.96	0.96	0.96	0.96	0.96	0.96	0.95	0.95	0.95	0.95	0.95	0.95
0.25	0.96	0.95	0.95	0.95	0.96	0.96	0.96	0.96	0.96	0.96	0.95	0.95	0.96	0.96	0.96	0.95	0.96	0.95	0.95	0.95	0.95	0.95	0.95	0.95	0.95
0.30	0.96	0.95	0.95	0.95	0.95	0.95	0.95	0.95	0.96	0.96	0.96	0.95	0.95	0.95	0.95	0.95	0.95	0.95	0.95	0.96	0.96	0.96	0.96	0.96	0.96
0.35	0.96	0.95	0.95	0.95	0.95	0.96	0.95	0.95	0.95	0.95	0.95	0.95	0.95	0.96	0.95	0.96	0.96	0.96	0.95	0.96	0.96	0.96	0.96	0.96	0.96
0.40	0.96	0.95	0.95	0.95	0.95	0.95	0.95	0.95	0.95	0.95	0.95	0.95	0.95	0.95	0.95	0.95	0.95	0.95	0.95	0.95	0.95	0.95	0.95	0.95	0.95
0.45	0.96	0.95	0.95	0.95	0.95	0.95	0.95	0.95	0.95	0.95	0.95	0.95	0.95	0.95	0.95	0.95	0.95	0.95	0.95	0.95	0.95	0.95	0.95	0.95	0.95
0.50	0.96	0.95	0.95	0.95	0.95	0.95	0.95	0.95	0.95	0.95	0.95	0.95	0.95	0.95	0.95	0.95	0.95	0.95	0.95	0.95	0.95	0.95	0.95	0.95	0.95
0.55	0.96	0.95	0.95	0.95	0.95	0.95	0.95	0.95	0.95	0.95	0.95	0.95	0.95	0.95	0.95	0.95	0.94	0.95	0.95	0.95	0.95	0.95	0.95	0.95	0.95
0.6	0.96	0.95	0.95	0.95	0.95	0.95	0.95	0.95	0.95	0.95	0.95	0.95	0.95	0.95	0.95	0.95	0.95	0.95	0.95	0.95	0.95	0.95	0.95	0.95	0.95
0.65	0.96	0.95	0.95	0.95	0.95	0.95	0.95	0.95	0.95	0.95	0.95	0.95	0.95	0.95	0.95	0.95	0.95	0.95	0.95	0.95	0.95	0.95	0.95	0.95	0.96
0.70	0.96	0.95	0.95	0.95	0.95	0.95	0.95	0.95	0.95	0.95	0.95	0.95	0.95	0.95	0.95	0.95	0.94	0.94	0.95	0.95	0.95	0.95	0.95	0.95	0.96
0.75	0.96	0.95	0.95	0.95	0.95	0.95	0.95	0.95	0.95	0.95	0.95	0.95	0.95	0.95	0.95	0.95	0.94	0.94	0.95	0.95	0.95	0.95	0.95	0.95	0.95
0.80	0.96	0.96	0.95	0.95	0.95	0.95	0.95	0.95	0.95	0.95	0.95	0.95	0.95	0.94	0.95	0.95	0.94	0.94	0.95	0.95	0.95	0.95	0.95	0.95	0.95
0.85	0.96	0.95	0.95	0.95	0.95	0.95	0.95	0.95	0.95	0.95	0.95	0.95	0.95	0.94	0.94	0.94	0.94	0.94	0.94	0.94	0.94	0.94	0.94	0.95	0.96
0.90	0.96	0.95	0.95	0.95	0.95	0.95	0.95	0.95	0.95	0.95	0.95	0.95	0.96	0.95	0.95	0.95	0.94	0.94	0.94	0.94	0.94	0.94	0.95	0.95	0.94
0.92	0.96	0.95	0.95	0.95	0.95	0.95	0.95	0.95	0.95	0.95	0.96	0.95	0.96	0.95	0.95	0.95	0.95	0.95	0.95	0.95	0.94	0.94	0.94	0.94	0.97
0.94	0.96	0.95	0.95	0.95	0.96	0.95	0.95	0.95	0.95	0.95	0.96	0.96	0.96	0.95	0.96	0.96	0.96	0.96	0.96	0.96	0.97	0.97	0.97	0.97	0.97
0.96	0.96	0.95	0.95	0.95	0.95	0.95	0.95	0.96	0.96	0.96	0.96	0.96	0.96	0.96	0.96	0.96	0.97	0.97	0.97	0.97	0.98	0.98	0.98	0.97	0.97
0.98	0.95	0.95	0.96	0.95	0.95	0.96	0.95	0.96	0.96	0.96	0.96	0.96	0.96	0.96	0.96	0.96	0.97	0.97	0.97	0.97	0.98	0.97	0.97	0.97	0.96

(i) n_{xrf}=400, n_{yrf} = 400

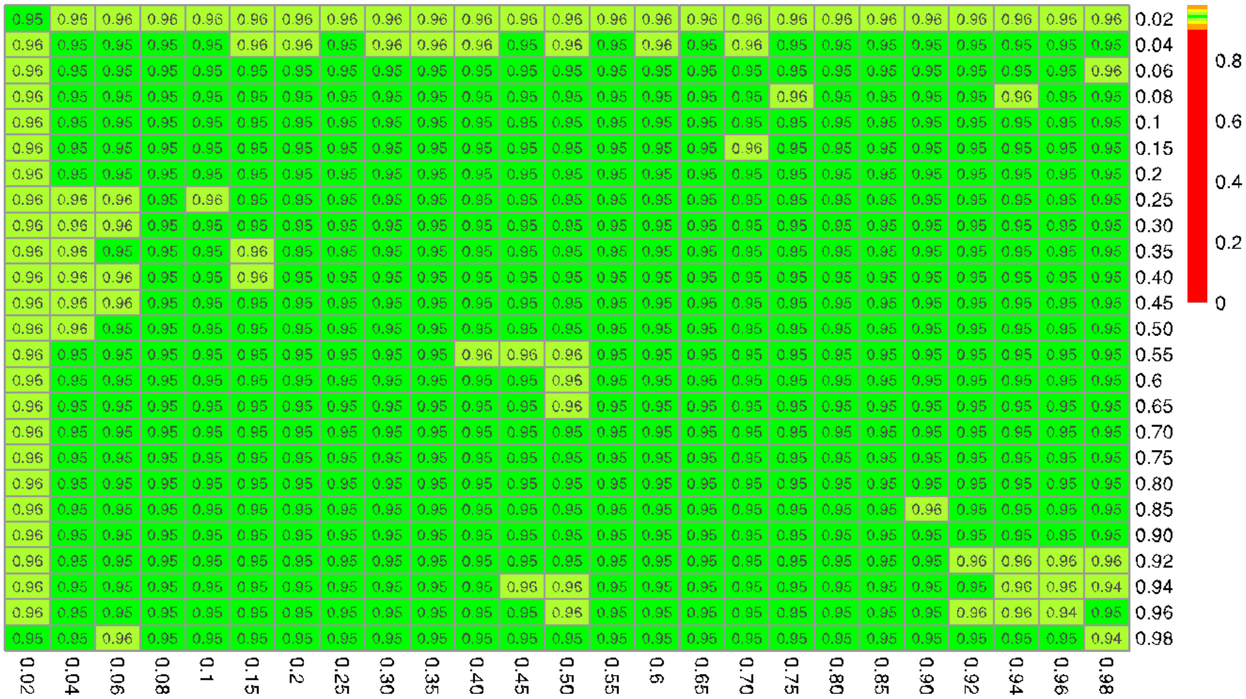

(j) n_{xrf}=500, n_{yrf} = 50

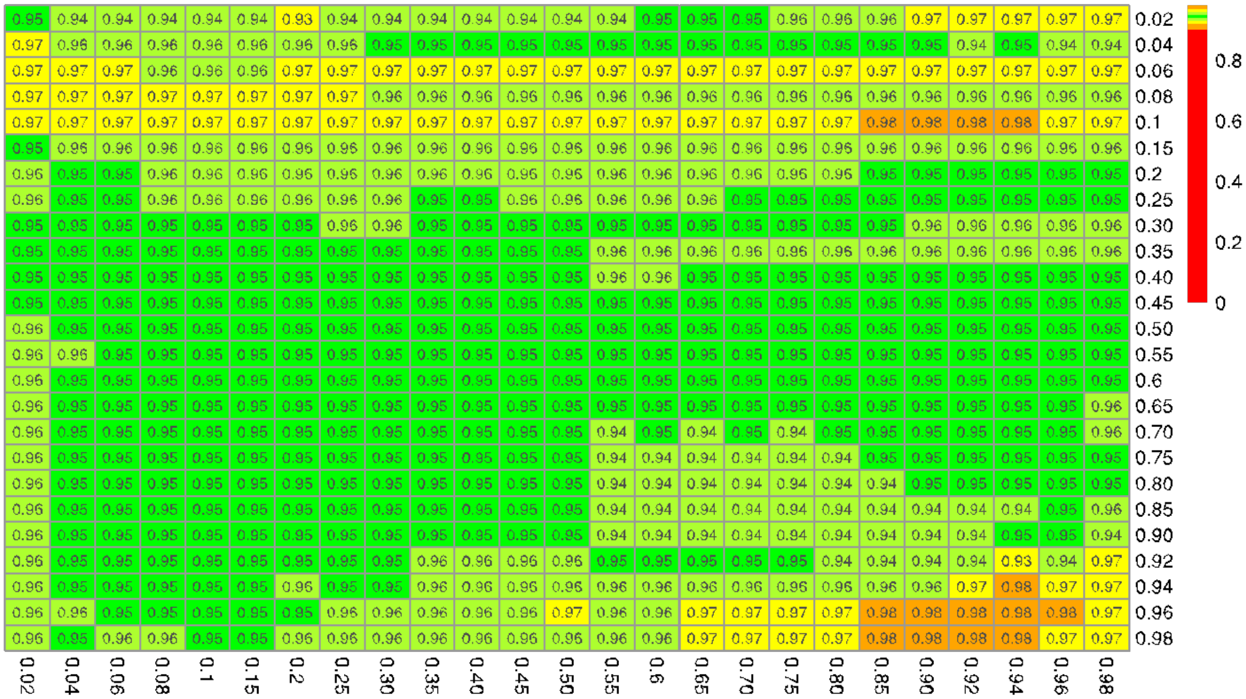

(k) n_{xrf}=500, n_{yrf} = 500

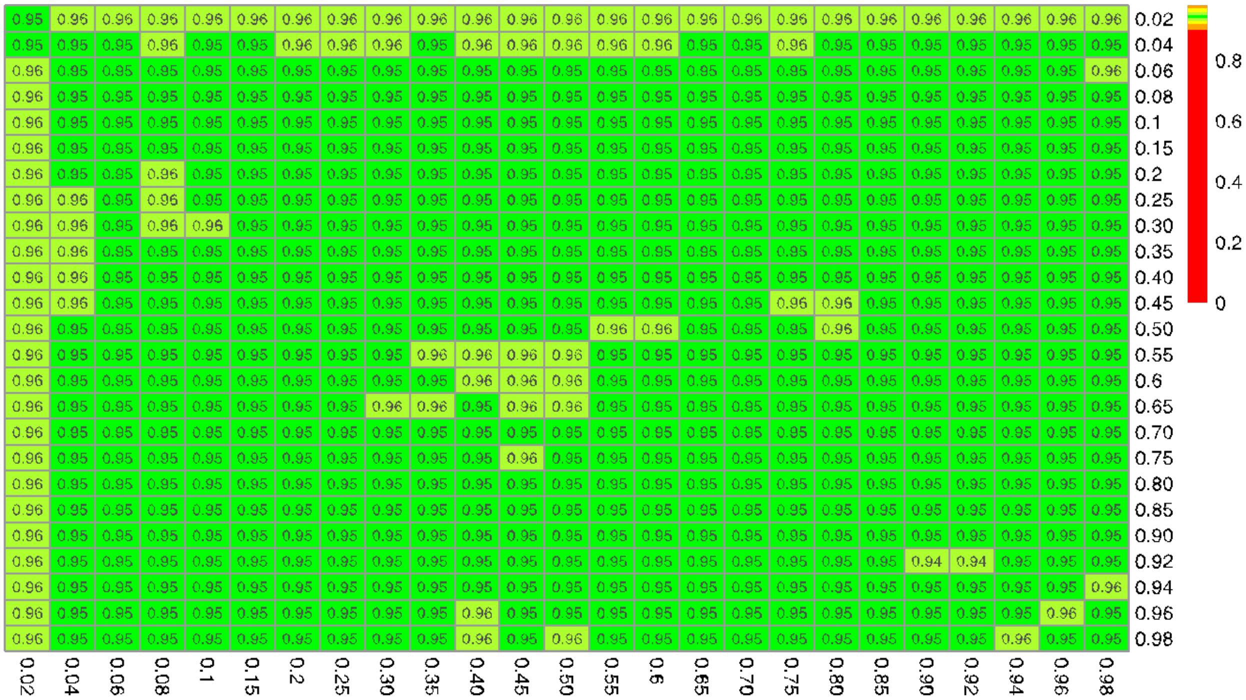

Appendix S4 Figure 3: Estimated 95% coverage probabilities for the confidence intervals constructed using the parametric bootstrap method.

(a) $n_{xrf} = 50$, $n_{yrf} = 50$

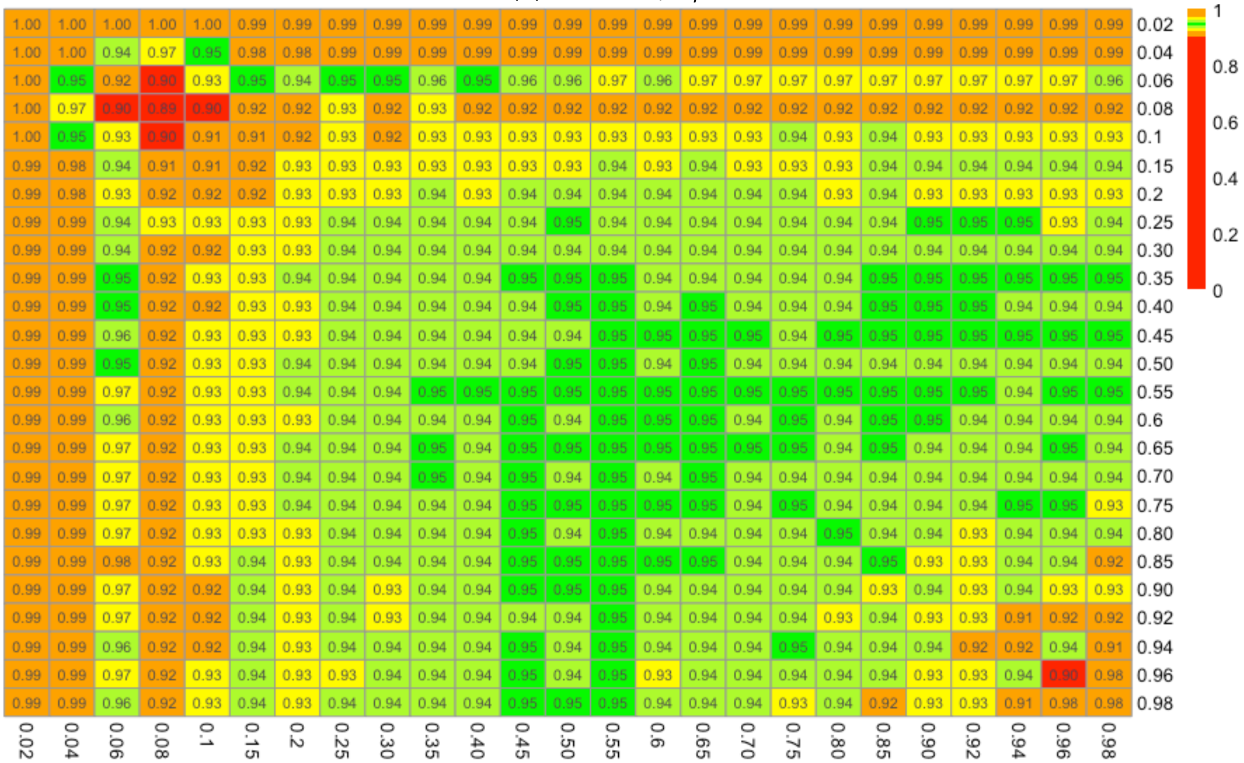

(b) $n_{xrf} = 100$, $n_{yrf} = 50$

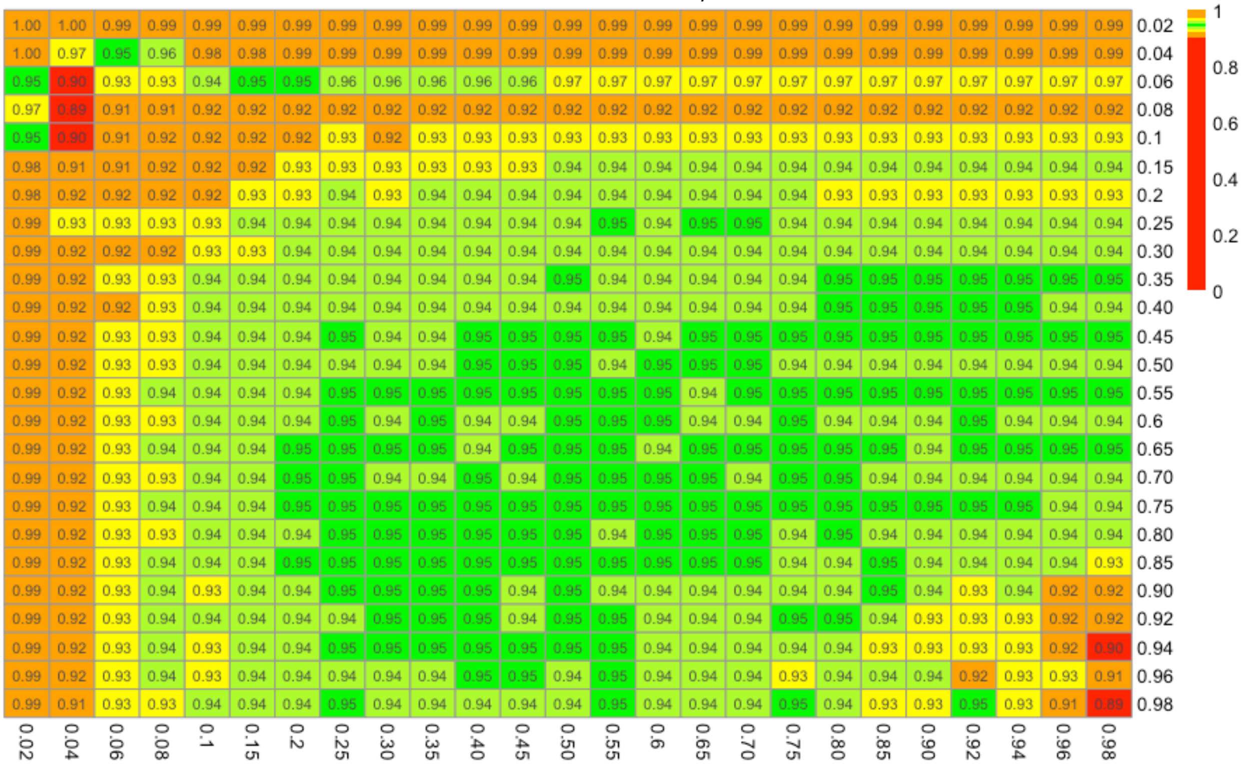

(c) $n_{xrf} = 100$, $n_{yrf} = 100$

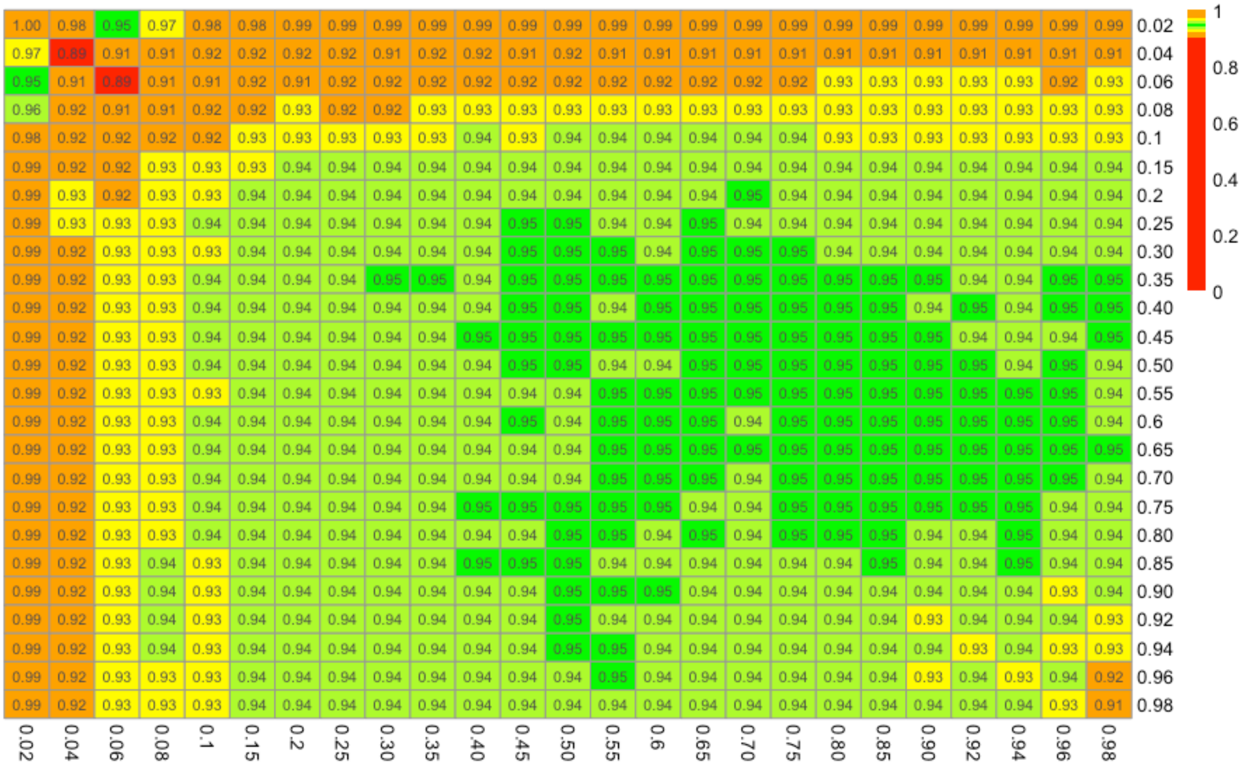

(d) $n_{xrf} = 200$, $n_{yrf} = 50$

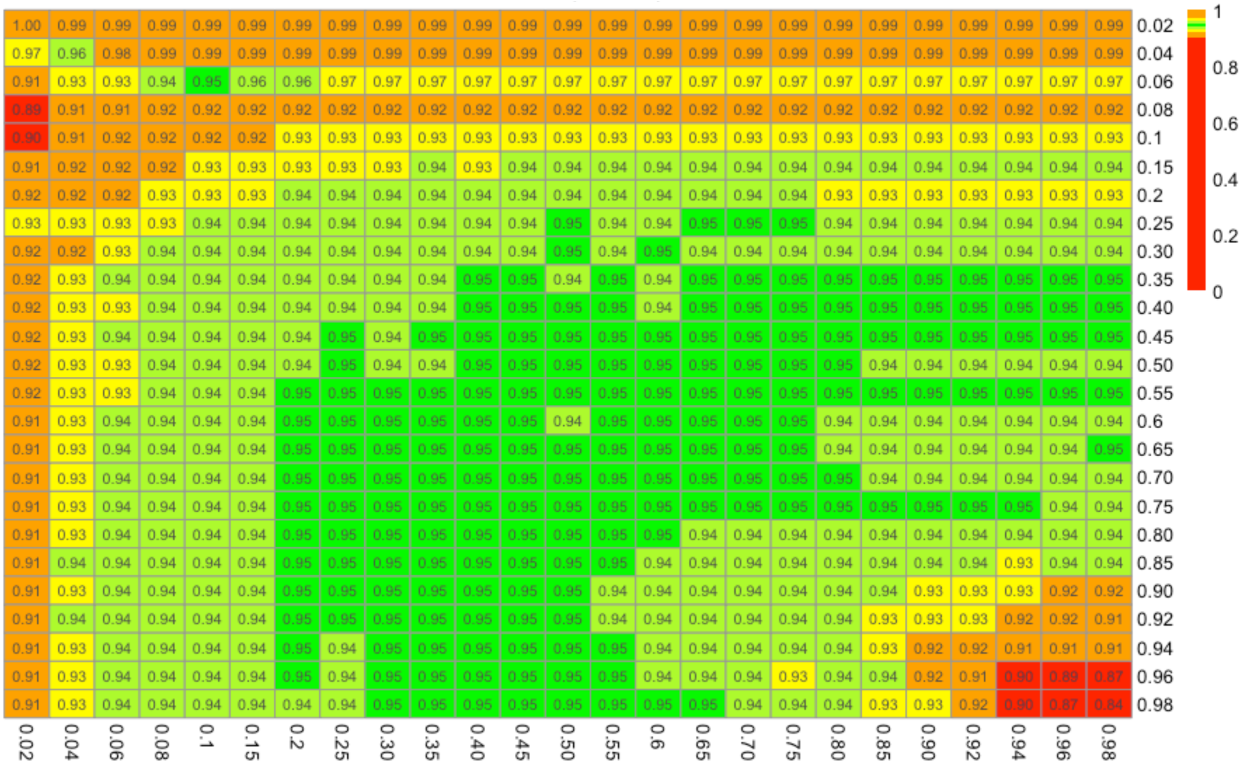

(e) $n_{xrf} = 200$, $n_{yrf} = 200$

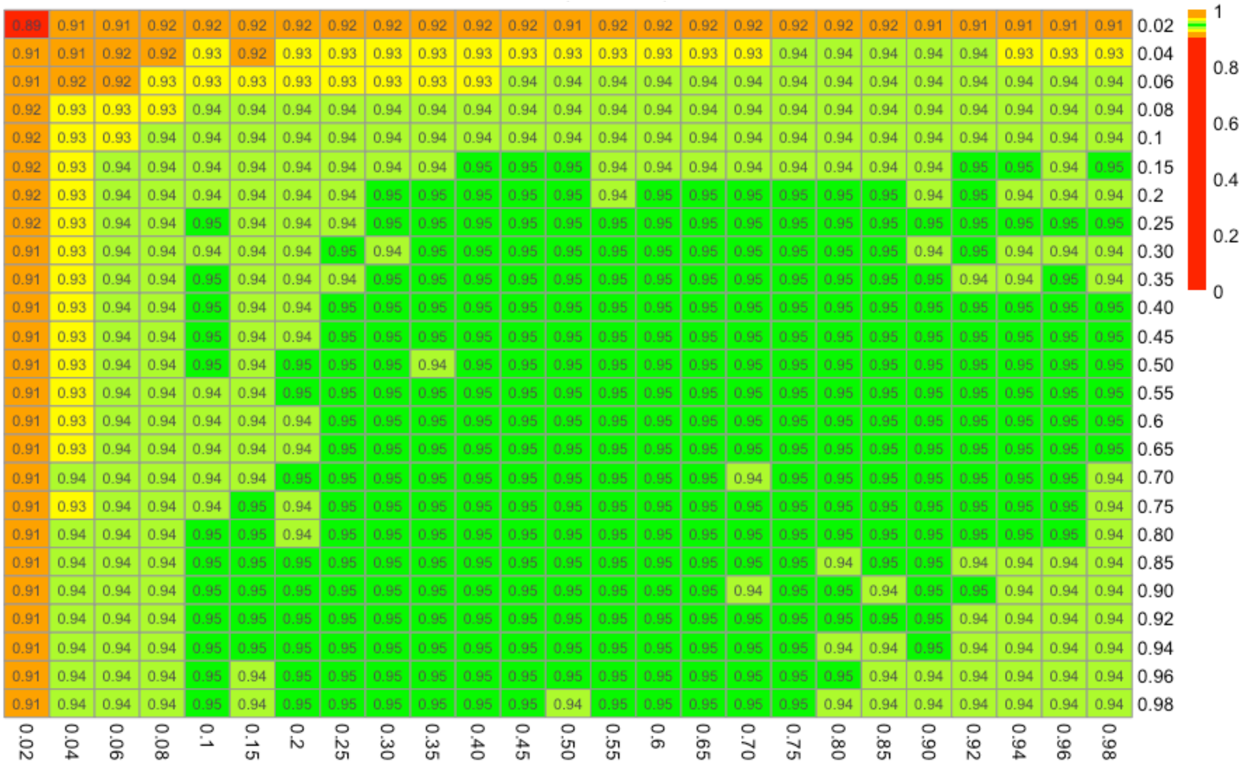

(f) $n_{xrf} = 300$, $n_{yrf} = 50$

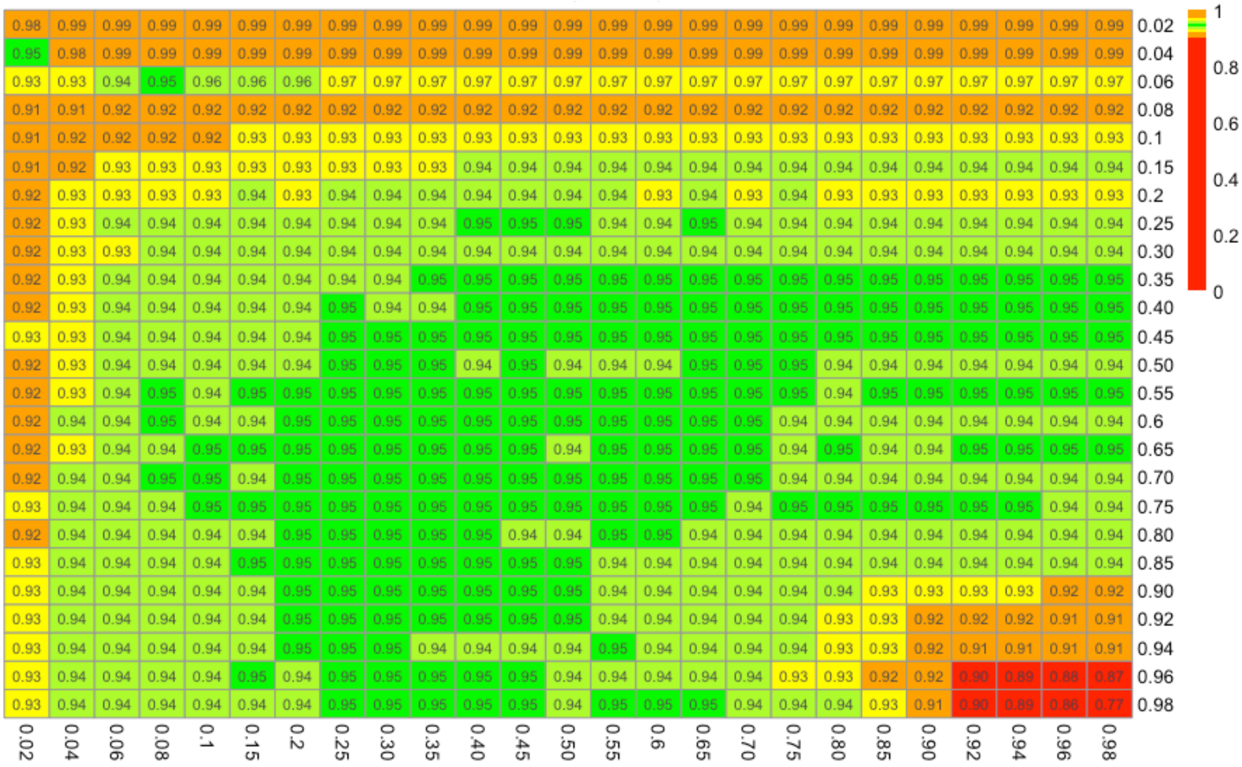

(g) n_{xrf}=300, n_{yrf}=300

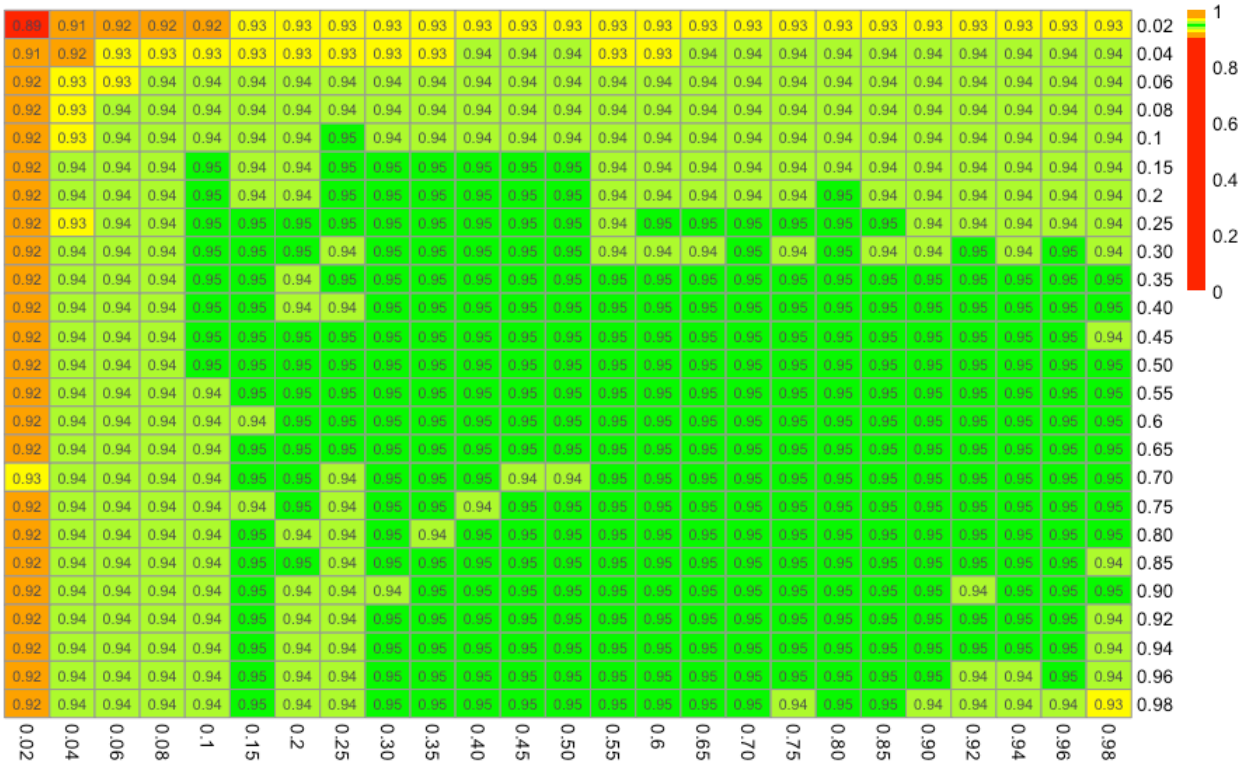

(h) n_{xrf}=400, n_{yrf}=50

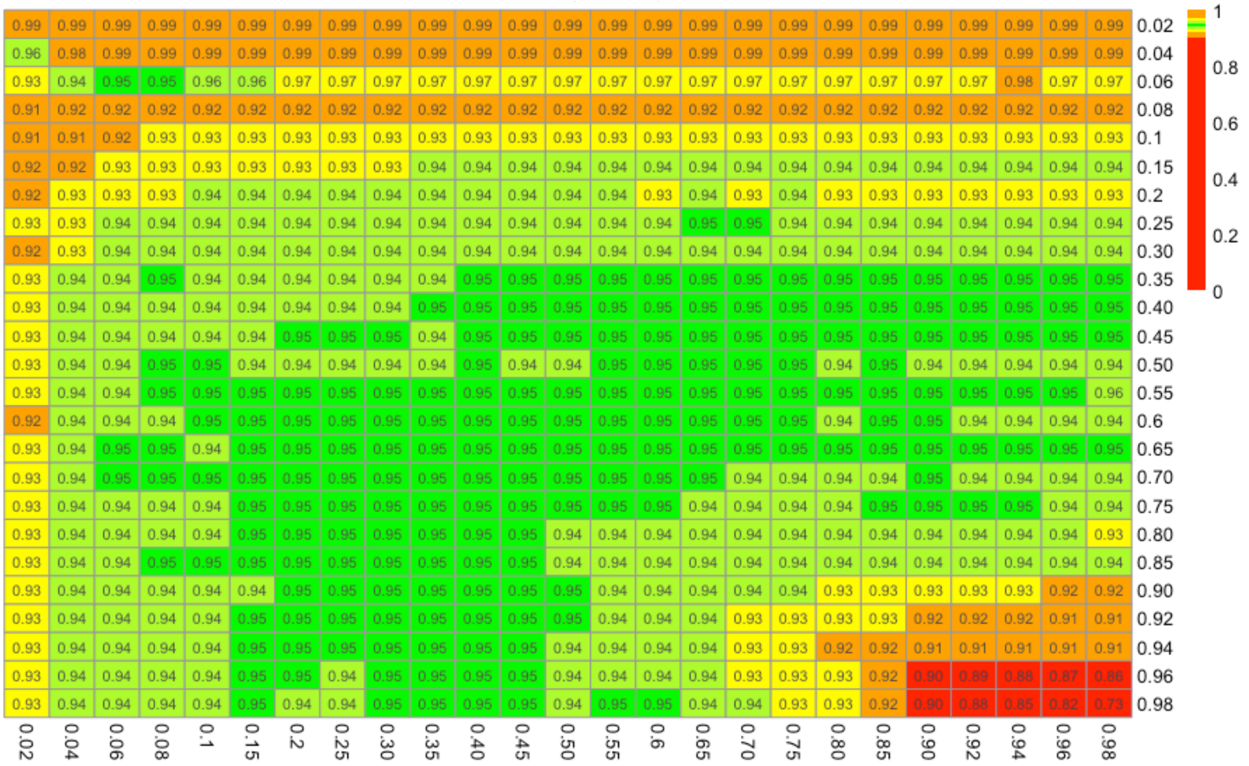

(i) n_{xrf}=400, n_{yrf}=400

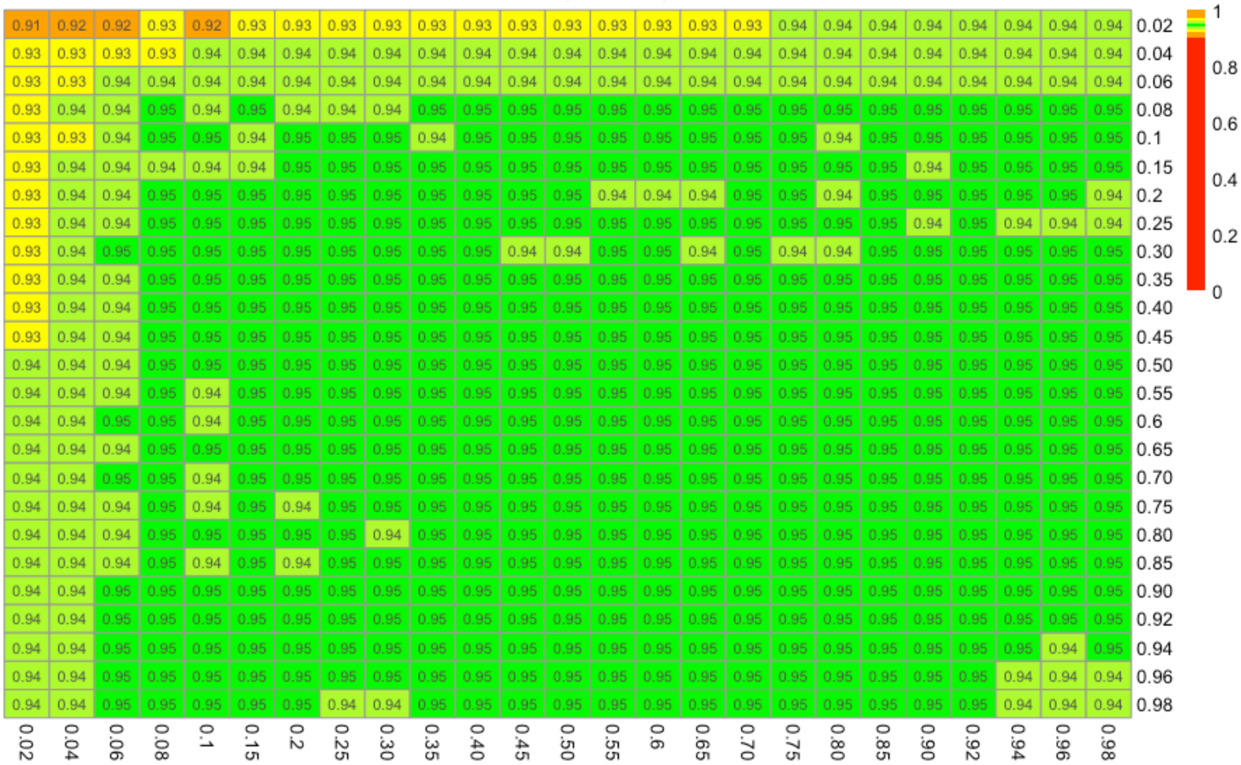

(j) n_{xrf}=500, n_{yrf}=50

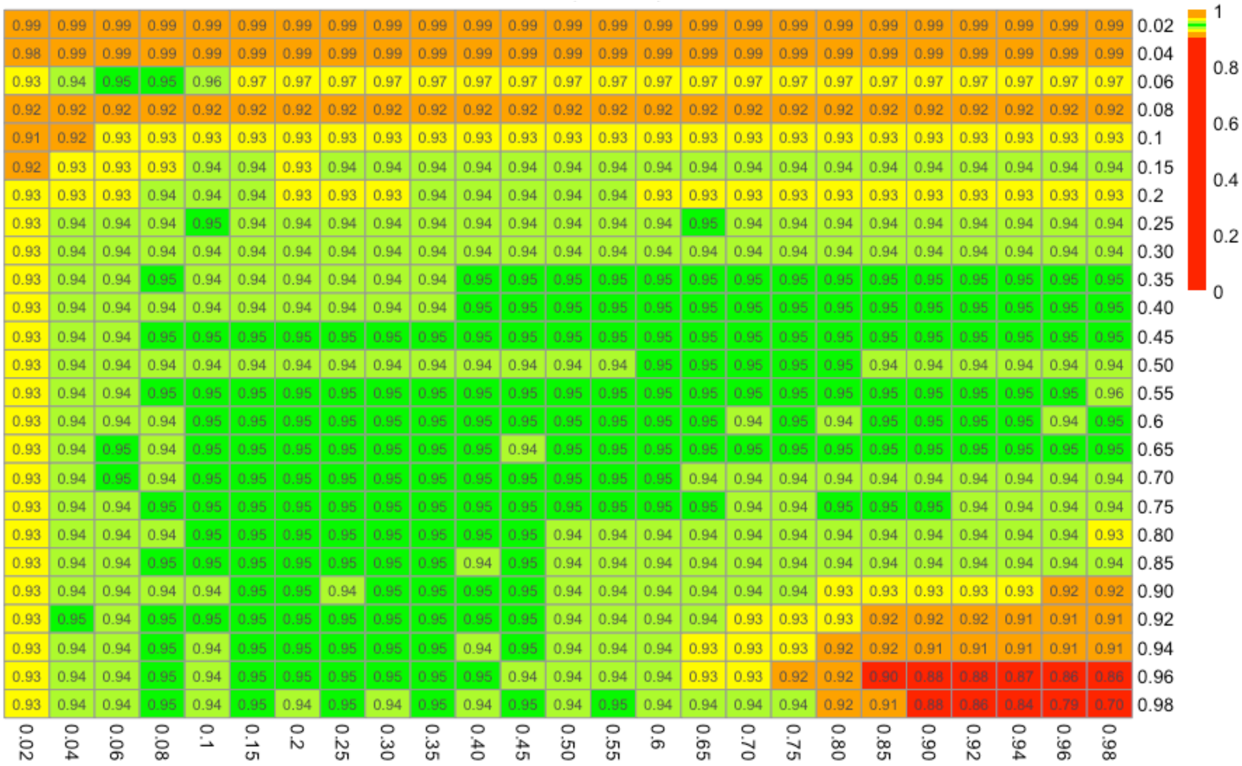

(k) n_{xrf}=500, n_{yrf}=500

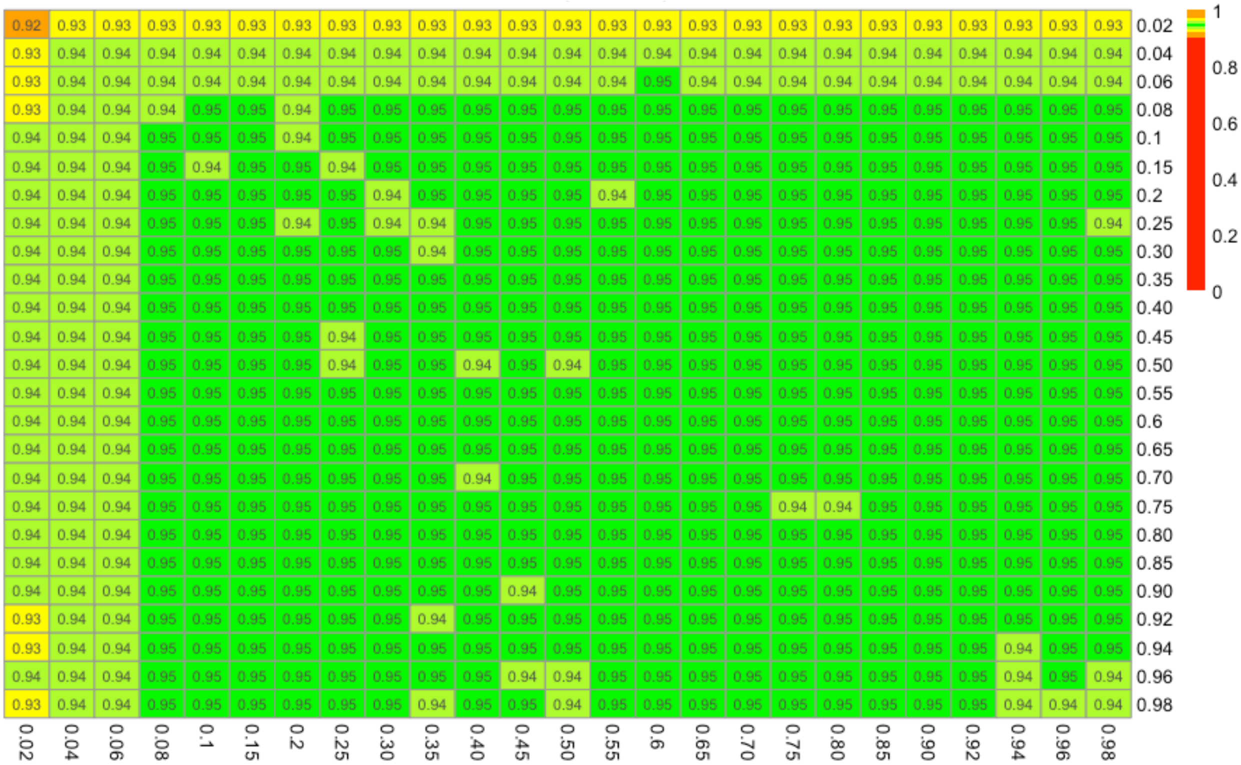

Appendix S4 Figure 4: Proportion of invalid confidence intervals constructed using the exact method

(a) n_{xrf} = 50, n_{yrf} = 50

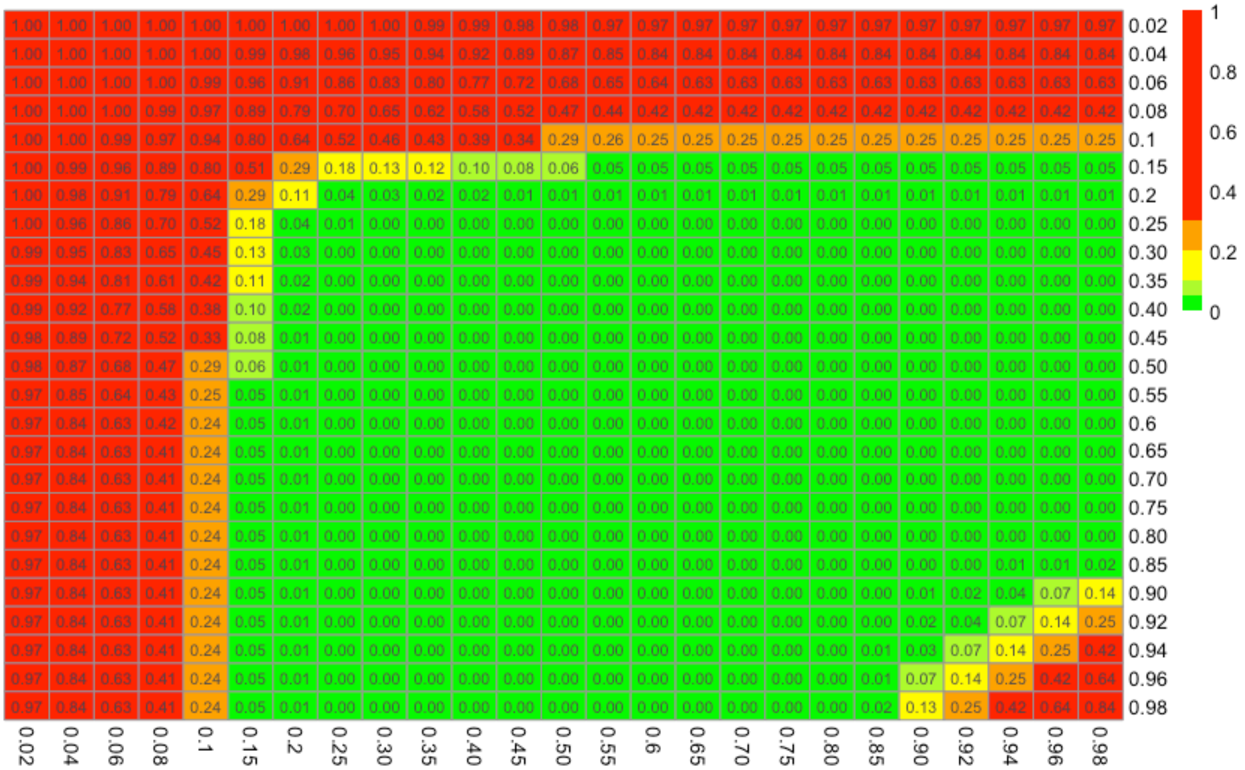

(b) n_{xrf} = 100, n_{yrf} = 50

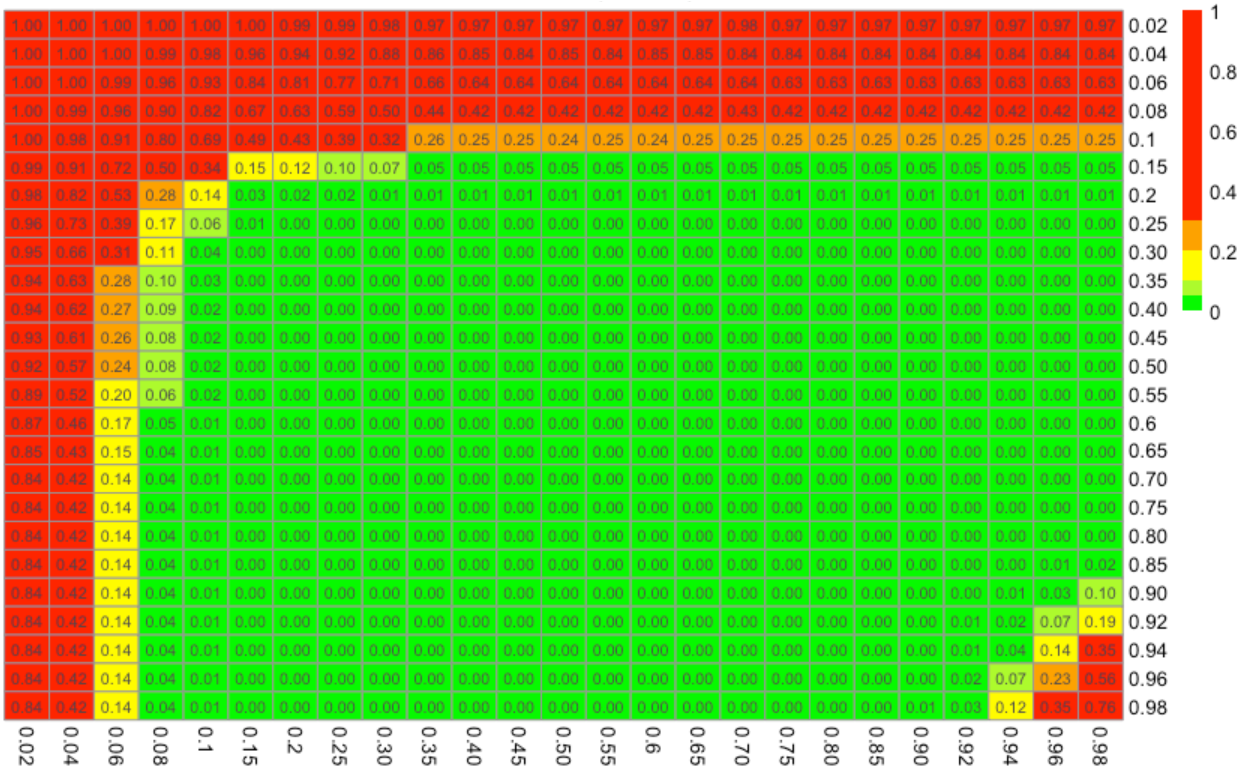

(c) $n_{xrf} = 100$, $n_{yrf} = 100$

	0.02	0.04	0.06	0.08	0.1	0.15	0.2	0.25	0.30	0.35	0.40	0.45	0.50	0.55	0.6	0.65	0.70	0.75	0.80	0.85	0.90	0.92	0.94	0.96	0.98
0.02	1.00	1.00	1.00	1.00	0.99	0.96	0.94	0.94	0.94	0.92	0.87	0.85	0.84	0.84	0.84	0.84	0.84	0.84	0.84	0.84	0.84	0.84	0.84	0.84	0.84
0.04	1.00	0.99	0.97	0.93	0.86	0.71	0.64	0.63	0.62	0.57	0.49	0.44	0.42	0.42	0.42	0.43	0.43	0.42	0.42	0.42	0.42	0.42	0.42	0.42	0.42
0.06	1.00	0.97	0.88	0.75	0.61	0.38	0.29	0.28	0.27	0.23	0.18	0.15	0.14	0.14	0.14	0.14	0.14	0.15	0.15	0.15	0.15	0.15	0.15	0.15	0.15
0.08	0.99	0.92	0.75	0.53	0.36	0.15	0.10	0.09	0.09	0.08	0.05	0.04	0.04	0.04	0.04	0.04	0.04	0.04	0.04	0.04	0.04	0.04	0.04	0.04	0.04
0.1	0.99	0.87	0.61	0.36	0.19	0.05	0.03	0.02	0.02	0.02	0.01	0.01	0.01	0.01	0.01	0.01	0.01	0.01	0.01	0.01	0.01	0.01	0.01	0.01	0.01
0.15	0.96	0.71	0.37	0.15	0.05	0.00	0.00	0.00	0.00	0.00	0.00	0.00	0.00	0.00	0.00	0.00	0.00	0.00	0.00	0.00	0.00	0.00	0.00	0.00	0.00
0.2	0.94	0.63	0.28	0.10	0.03	0.00	0.00	0.00	0.00	0.00	0.00	0.00	0.00	0.00	0.00	0.00	0.00	0.00	0.00	0.00	0.00	0.00	0.00	0.00	0.00
0.25	0.94	0.62	0.27	0.09	0.02	0.00	0.00	0.00	0.00	0.00	0.00	0.00	0.00	0.00	0.00	0.00	0.00	0.00	0.00	0.00	0.00	0.00	0.00	0.00	0.00
0.30	0.94	0.61	0.26	0.09	0.02	0.00	0.00	0.00	0.00	0.00	0.00	0.00	0.00	0.00	0.00	0.00	0.00	0.00	0.00	0.00	0.00	0.00	0.00	0.00	0.00
0.35	0.91	0.56	0.23	0.07	0.02	0.00	0.00	0.00	0.00	0.00	0.00	0.00	0.00	0.00	0.00	0.00	0.00	0.00	0.00	0.00	0.00	0.00	0.00	0.00	0.00
0.40	0.87	0.48	0.18	0.05	0.01	0.00	0.00	0.00	0.00	0.00	0.00	0.00	0.00	0.00	0.00	0.00	0.00	0.00	0.00	0.00	0.00	0.00	0.00	0.00	0.00
0.45	0.85	0.43	0.15	0.04	0.01	0.00	0.00	0.00	0.00	0.00	0.00	0.00	0.00	0.00	0.00	0.00	0.00	0.00	0.00	0.00	0.00	0.00	0.00	0.00	0.00
0.50	0.84	0.42	0.14	0.04	0.01	0.00	0.00	0.00	0.00	0.00	0.00	0.00	0.00	0.00	0.00	0.00	0.00	0.00	0.00	0.00	0.00	0.00	0.00	0.00	0.00
0.55	0.84	0.42	0.14	0.04	0.01	0.00	0.00	0.00	0.00	0.00	0.00	0.00	0.00	0.00	0.00	0.00	0.00	0.00	0.00	0.00	0.00	0.00	0.00	0.00	0.00
0.6	0.84	0.42	0.14	0.04	0.01	0.00	0.00	0.00	0.00	0.00	0.00	0.00	0.00	0.00	0.00	0.00	0.00	0.00	0.00	0.00	0.00	0.00	0.00	0.00	0.00
0.65	0.84	0.42	0.14	0.04	0.01	0.00	0.00	0.00	0.00	0.00	0.00	0.00	0.00	0.00	0.00	0.00	0.00	0.00	0.00	0.00	0.00	0.00	0.00	0.00	0.00
0.70	0.84	0.42	0.14	0.04	0.01	0.00	0.00	0.00	0.00	0.00	0.00	0.00	0.00	0.00	0.00	0.00	0.00	0.00	0.00	0.00	0.00	0.00	0.00	0.00	0.00
0.75	0.84	0.42	0.14	0.04	0.01	0.00	0.00	0.00	0.00	0.00	0.00	0.00	0.00	0.00	0.00	0.00	0.00	0.00	0.00	0.00	0.00	0.00	0.00	0.00	0.00
0.80	0.84	0.42	0.14	0.04	0.01	0.00	0.00	0.00	0.00	0.00	0.00	0.00	0.00	0.00	0.00	0.00	0.00	0.00	0.00	0.00	0.00	0.00	0.00	0.00	0.00
0.85	0.84	0.42	0.14	0.04	0.01	0.00	0.00	0.00	0.00	0.00	0.00	0.00	0.00	0.00	0.00	0.00	0.00	0.00	0.00	0.00	0.00	0.00	0.00	0.00	0.00
0.90	0.84	0.42	0.14	0.04	0.01	0.00	0.00	0.00	0.00	0.00	0.00	0.00	0.00	0.00	0.00	0.00	0.00	0.00	0.00	0.00	0.00	0.00	0.00	0.00	0.00
0.92	0.84	0.42	0.14	0.04	0.01	0.00	0.00	0.00	0.00	0.00	0.00	0.00	0.00	0.00	0.00	0.00	0.00	0.00	0.00	0.00	0.00	0.00	0.00	0.00	0.01
0.94	0.84	0.42	0.14	0.04	0.01	0.00	0.00	0.00	0.00	0.00	0.00	0.00	0.00	0.00	0.00	0.00	0.00	0.00	0.00	0.00	0.00	0.00	0.00	0.01	0.04
0.96	0.84	0.42	0.14	0.04	0.01	0.00	0.00	0.00	0.00	0.00	0.00	0.00	0.00	0.00	0.00	0.00	0.00	0.00	0.00	0.00	0.00	0.00	0.01	0.04	0.14
0.98	0.84	0.42	0.14	0.04	0.01	0.00	0.00	0.00	0.00	0.00	0.00	0.00	0.00	0.00	0.00	0.00	0.00	0.00	0.00	0.00	0.00	0.01	0.04	0.14	0.42

(d) $n_{xrf} = 200$, $n_{yrf} = 50$

	0.02	0.04	0.06	0.08	0.1	0.15	0.2	0.25	0.30	0.35	0.40	0.45	0.50	0.55	0.6	0.65	0.70	0.75	0.80	0.85	0.90	0.92	0.94	0.96	0.98
0.02	1.00	1.00	1.00	1.00	1.00	0.99	0.98	0.97	0.97	0.97	0.97	0.97	0.97	0.97	0.97	0.97	0.97	0.97	0.97	0.97	0.97	0.97	0.97	0.97	0.97
0.04	1.00	0.99	0.97	0.96	0.95	0.92	0.86	0.84	0.84	0.84	0.85	0.85	0.84	0.85	0.85	0.84	0.84	0.84	0.85	0.84	0.84	0.84	0.84	0.84	0.84
0.06	1.00	0.96	0.90	0.84	0.82	0.77	0.66	0.64	0.64	0.64	0.64	0.64	0.64	0.64	0.64	0.64	0.64	0.64	0.64	0.64	0.63	0.63	0.63	0.63	0.63
0.08	0.99	0.90	0.77	0.67	0.64	0.58	0.45	0.42	0.42	0.42	0.42	0.42	0.42	0.42	0.42	0.42	0.42	0.42	0.43	0.43	0.42	0.42	0.42	0.42	0.42
0.1	0.98	0.81	0.61	0.48	0.44	0.39	0.27	0.24	0.24	0.24	0.25	0.24	0.24	0.24	0.25	0.24	0.24	0.24	0.25	0.25	0.25	0.25	0.25	0.25	0.25
0.15	0.91	0.51	0.25	0.15	0.12	0.10	0.05	0.05	0.05	0.05	0.05	0.05	0.05	0.05	0.05	0.05	0.05	0.05	0.05	0.05	0.05	0.05	0.05	0.05	0.05
0.2	0.83	0.30	0.08	0.03	0.02	0.02	0.01	0.01	0.01	0.01	0.01	0.01	0.01	0.01	0.01	0.01	0.01	0.01	0.01	0.01	0.01	0.01	0.01	0.01	0.01
0.25	0.75	0.18	0.03	0.01	0.00	0.00	0.00	0.00	0.00	0.00	0.00	0.00	0.00	0.00	0.00	0.00	0.00	0.00	0.00	0.00	0.00	0.00	0.00	0.00	0.00
0.30	0.68	0.13	0.01	0.00	0.00	0.00	0.00	0.00	0.00	0.00	0.00	0.00	0.00	0.00	0.00	0.00	0.00	0.00	0.00	0.00	0.00	0.00	0.00	0.00	0.00
0.35	0.64	0.10	0.01	0.00	0.00	0.00	0.00	0.00	0.00	0.00	0.00	0.00	0.00	0.00	0.00	0.00	0.00	0.00	0.00	0.00	0.00	0.00	0.00	0.00	0.00
0.40	0.62	0.10	0.01	0.00	0.00	0.00	0.00	0.00	0.00	0.00	0.00	0.00	0.00	0.00	0.00	0.00	0.00	0.00	0.00	0.00	0.00	0.00	0.00	0.00	0.00
0.45	0.62	0.09	0.01	0.00	0.00	0.00	0.00	0.00	0.00	0.00	0.00	0.00	0.00	0.00	0.00	0.00	0.00	0.00	0.00	0.00	0.00	0.00	0.00	0.00	0.00
0.50	0.61	0.09	0.01	0.00	0.00	0.00	0.00	0.00	0.00	0.00	0.00	0.00	0.00	0.00	0.00	0.00	0.00	0.00	0.00	0.00	0.00	0.00	0.00	0.00	0.00
0.55	0.58	0.08	0.01	0.00	0.00	0.00	0.00	0.00	0.00	0.00	0.00	0.00	0.00	0.00	0.00	0.00	0.00	0.00	0.00	0.00	0.00	0.00	0.00	0.00	0.00
0.6	0.53	0.07	0.00	0.00	0.00	0.00	0.00	0.00	0.00	0.00	0.00	0.00	0.00	0.00	0.00	0.00	0.00	0.00	0.00	0.00	0.00	0.00	0.00	0.00	0.00
0.65	0.47	0.05	0.00	0.00	0.00	0.00	0.00	0.00	0.00	0.00	0.00	0.00	0.00	0.00	0.00	0.00	0.00	0.00	0.00	0.00	0.00	0.00	0.00	0.00	0.00
0.70	0.43	0.04	0.00	0.00	0.00	0.00	0.00	0.00	0.00	0.00	0.00	0.00	0.00	0.00	0.00	0.00	0.00	0.00	0.00	0.00	0.00	0.00	0.00	0.00	0.00
0.75	0.42	0.04	0.00	0.00	0.00	0.00	0.00	0.00	0.00	0.00	0.00	0.00	0.00	0.00	0.00	0.00	0.00	0.00	0.00	0.00	0.00	0.00	0.00	0.00	0.00
0.80	0.42	0.04	0.00	0.00	0.00	0.00	0.00	0.00	0.00	0.00	0.00	0.00	0.00	0.00	0.00	0.00	0.00	0.00	0.00	0.00	0.00	0.00	0.00	0.00	0.00
0.85	0.42	0.04	0.00	0.00	0.00	0.00	0.00	0.00	0.00	0.00	0.00	0.00	0.00	0.00	0.00	0.00	0.00	0.00	0.00	0.00	0.00	0.00	0.00	0.00	0.01
0.90	0.42	0.04	0.00	0.00	0.00	0.00	0.00	0.00	0.00	0.00	0.00	0.00	0.00	0.00	0.00	0.00	0.00	0.00	0.00	0.00	0.00	0.00	0.00	0.01	0.07
0.92	0.42	0.04	0.00	0.00	0.00	0.00	0.00	0.00	0.00	0.00	0.00	0.00	0.00	0.00	0.00	0.00	0.00	0.00	0.00	0.00	0.00	0.00	0.00	0.01	0.13
0.94	0.42	0.04	0.00	0.00	0.00	0.00	0.00	0.00	0.00	0.00	0.00	0.00	0.00	0.00	0.00	0.00	0.00	0.00	0.00	0.00	0.00	0.00	0.00	0.02	0.23
0.96	0.42	0.04	0.00	0.00	0.00	0.00	0.00	0.00	0.00	0.00	0.00	0.00	0.00	0.00	0.00	0.00	0.00	0.00	0.00	0.00	0.00	0.00	0.00	0.04	0.35
0.98	0.42	0.04	0.00	0.00	0.00	0.00	0.00	0.00	0.00	0.00	0.00	0.00	0.00	0.00	0.00	0.00	0.00	0.00	0.00	0.00	0.00	0.00	0.00	0.05	0.47

(e) $n_{xrf} = 200$, $n_{yrf} = 200$

	0.02	0.04	0.06	0.08	0.1	0.15	0.2	0.25	0.30	0.35	0.40	0.45	0.50	0.55	0.6	0.65	0.70	0.75	0.80	0.85	0.90	0.92	0.94	0.96	0.98
0.02	0.99	0.93	0.83	0.73	0.66	0.63	0.63	0.60	0.49	0.42	0.42	0.42	0.42	0.42	0.42	0.42	0.42	0.42	0.43	0.43	0.42	0.42	0.42	0.42	0.42
0.04	0.93	0.56	0.28	0.17	0.12	0.10	0.10	0.09	0.06	0.04	0.04	0.04	0.04	0.04	0.04	0.04	0.04	0.04	0.04	0.04	0.04	0.04	0.04	0.04	0.04
0.06	0.83	0.28	0.06	0.02	0.01	0.01	0.01	0.01	0.00	0.00	0.00	0.00	0.00	0.00	0.00	0.00	0.00	0.00	0.00	0.00	0.00	0.00	0.00	0.00	0.00
0.08	0.73	0.16	0.02	0.00	0.00	0.00	0.00	0.00	0.00	0.00	0.00	0.00	0.00	0.00	0.00	0.00	0.00	0.00	0.00	0.00	0.00	0.00	0.00	0.00	0.00
0.1	0.66	0.11	0.01	0.00	0.00	0.00	0.00	0.00	0.00	0.00	0.00	0.00	0.00	0.00	0.00	0.00	0.00	0.00	0.00	0.00	0.00	0.00	0.00	0.00	0.00
0.15	0.62	0.09	0.01	0.00	0.00	0.00	0.00	0.00	0.00	0.00	0.00	0.00	0.00	0.00	0.00	0.00	0.00	0.00	0.00	0.00	0.00	0.00	0.00	0.00	0.00
0.2	0.62	0.09	0.01	0.00	0.00	0.00	0.00	0.00	0.00	0.00	0.00	0.00	0.00	0.00	0.00	0.00	0.00	0.00	0.00	0.00	0.00	0.00	0.00	0.00	0.00
0.25	0.60	0.09	0.01	0.00	0.00	0.00	0.00	0.00	0.00	0.00	0.00	0.00	0.00	0.00	0.00	0.00	0.00	0.00	0.00	0.00	0.00	0.00	0.00	0.00	0.00
0.30	0.49	0.06	0.00	0.00	0.00	0.00	0.00	0.00	0.00	0.00	0.00	0.00	0.00	0.00	0.00	0.00	0.00	0.00	0.00	0.00	0.00	0.00	0.00	0.00	0.00
0.35	0.42	0.04	0.00	0.00	0.00	0.00	0.00	0.00	0.00	0.00	0.00	0.00	0.00	0.00	0.00	0.00	0.00	0.00	0.00	0.00	0.00	0.00	0.00	0.00	0.00
0.40	0.42	0.04	0.00	0.00	0.00	0.00	0.00	0.00	0.00	0.00	0.00	0.00	0.00	0.00	0.00	0.00	0.00	0.00	0.00	0.00	0.00	0.00	0.00	0.00	0.00
0.45	0.42	0.04	0.00	0.00	0.00	0.00	0.00	0.00	0.00	0.00	0.00	0.00	0.00	0.00	0.00	0.00	0.00	0.00	0.00	0.00	0.00	0.00	0.00	0.00	0.00
0.50	0.42	0.04	0.00	0.00	0.00	0.00	0.00	0.00	0.00	0.00	0.00	0.00	0.00	0.00	0.00	0.00	0.00	0.00	0.00	0.00	0.00	0.00	0.00	0.00	0.00
0.55	0.42	0.04	0.00	0.00	0.00	0.00	0.00	0.00	0.00	0.00	0.00	0.00	0.00	0.00	0.00	0.00	0.00	0.00	0.00	0.00	0.00	0.00	0.00	0.00	0.00
0.6	0.42	0.04	0.00	0.00	0.00	0.00	0.00	0.00	0.00	0.00	0.00	0.00	0.00	0.00	0.00	0.00	0.00	0.00	0.00	0.00	0.00	0.00	0.00	0.00	0.00
0.65	0.42	0.04	0.00	0.00	0.00	0.00	0.00	0.00	0.00	0.00	0.00	0.00	0.00	0.00	0.00	0.00	0.00	0.00	0.00	0.00	0.00	0.00	0.00	0.00	0.00
0.70	0.42	0.04	0.00	0.00	0.00	0.00	0.00	0.00	0.00	0.00	0.00	0.00	0.00	0.00	0.00	0.00	0.00	0.00	0.00	0.00	0.00	0.00	0.00	0.00	0.00
0.75	0.42	0.04	0.00	0.00	0.00	0.00	0.00	0.00	0.00	0.00	0.00	0.00	0.00	0.00	0.00	0.00	0.00	0.00	0.00	0.00	0.00	0.00	0.00	0.00	0.00
0.80	0.42	0.04	0.00	0.00	0.00	0.00	0.00	0.00	0.00	0.00	0.00	0.00	0.00	0.00	0.00	0.00	0.00	0.00	0.00	0.00	0.00	0.00	0.00	0.00	0.00
0.85	0.42	0.04	0.00	0.00	0.00	0.00	0.00	0.00	0.00	0.00	0.00	0.00	0.00	0.00	0.00	0.00	0.00	0.00	0.00	0.00	0.00	0.00	0.00	0.00	0.00
0.90	0.42	0.04	0.00	0.00	0.00	0.00	0.00	0.00	0.00	0.00	0.00	0.00	0.00	0.00	0.00	0.00	0.00	0.00	0.00	0.00	0.00	0.00	0.00	0.00	0.00
0.92	0.42	0.04	0.00	0.00	0.00	0.00	0.00	0.00	0.00	0.00	0.00	0.00	0.00	0.00	0.00	0.00	0.00	0.00	0.00	0.00	0.00	0.00	0.00	0.00	0.00
0.94	0.42	0.04	0.00	0.00	0.00	0.00	0.00	0.00	0.00	0.00	0.00	0.00	0.00	0.00	0.00	0.00	0.00	0.00	0.00	0.00	0.00	0.00	0.00	0.00	0.00
0.96	0.42	0.04	0.00	0.00	0.00	0.00	0.00	0.00	0.00	0.00	0.00	0.00	0.00	0.00	0.00	0.00	0.00	0.00	0.00	0.00	0.00	0.00	0.00	0.00	0.00
0.98	0.42	0.04	0.00	0.00	0.00	0.00	0.00	0.00	0.00	0.00	0.00	0.00	0.00	0.00	0.00	0.00	0.00	0.00	0.00	0.00	0.00	0.00	0.00	0.00	0.04

(f) $n_{xrf} = 300$, $n_{yrf} = 50$

	0.02	0.04	0.06	0.08	0.1	0.15	0.2	0.25	0.30	0.35	0.40	0.45	0.50	0.55	0.6	0.65	0.70	0.75	0.80	0.85	0.90	0.92	0.94	0.96	0.98
0.02	1.00	1.00	1.00	1.00	0.99	0.97	0.97	0.97	0.97	0.97	0.97	0.97	0.97	0.97	0.97	0.97	0.97	0.97	0.97	0.97	0.97	0.97	0.97	0.97	0.97
0.04	1.00	0.98	0.95	0.94	0.93	0.85	0.84	0.85	0.85	0.85	0.85	0.84	0.84	0.84	0.85	0.85	0.85	0.85	0.84	0.84	0.84	0.84	0.84	0.84	0.84
0.06	0.99	0.91	0.83	0.81	0.79	0.65	0.64	0.64	0.64	0.63	0.63	0.63	0.63	0.63	0.63	0.63	0.64	0.64	0.64	0.64	0.63	0.63	0.63	0.63	0.63
0.08	0.96	0.78	0.66	0.63	0.60	0.43	0.42	0.42	0.42	0.42	0.42	0.42	0.42	0.42	0.42	0.42	0.42	0.42	0.42	0.42	0.42	0.42	0.42	0.42	0.42
0.1	0.91	0.63	0.47	0.43	0.41	0.25	0.24	0.24	0.24	0.24	0.24	0.24	0.24	0.24	0.24	0.24	0.24	0.24	0.24	0.24	0.25	0.25	0.25	0.25	0.25
0.15	0.73	0.26	0.14	0.12	0.10	0.05	0.05	0.05	0.05	0.05	0.05	0.05	0.05	0.05	0.05	0.05	0.05	0.05	0.05	0.05	0.05	0.05	0.05	0.05	0.05
0.2	0.55	0.08	0.03	0.02	0.02	0.01	0.01	0.01	0.01	0.01	0.01	0.01	0.01	0.01	0.01	0.01	0.01	0.01	0.01	0.01	0.01	0.01	0.01	0.01	0.01
0.25	0.42	0.03	0.00	0.00	0.00	0.00	0.00	0.00	0.00	0.00	0.00	0.00	0.00	0.00	0.00	0.00	0.00	0.00	0.00	0.00	0.00	0.00	0.00	0.00	0.00
0.30	0.34	0.01	0.00	0.00	0.00	0.00	0.00	0.00	0.00	0.00	0.00	0.00	0.00	0.00	0.00	0.00	0.00	0.00	0.00	0.00	0.00	0.00	0.00	0.00	0.00
0.35	0.30	0.01	0.00	0.00	0.00	0.00	0.00	0.00	0.00	0.00	0.00	0.00	0.00	0.00	0.00	0.00	0.00	0.00	0.00	0.00	0.00	0.00	0.00	0.00	0.00
0.40	0.28	0.01	0.00	0.00	0.00	0.00	0.00	0.00	0.00	0.00	0.00	0.00	0.00	0.00	0.00	0.00	0.00	0.00	0.00	0.00	0.00	0.00	0.00	0.00	0.00
0.45	0.27	0.01	0.00	0.00	0.00	0.00	0.00	0.00	0.00	0.00	0.00	0.00	0.00	0.00	0.00	0.00	0.00	0.00	0.00	0.00	0.00	0.00	0.00	0.00	0.00
0.50	0.27	0.01	0.00	0.00	0.00	0.00	0.00	0.00	0.00	0.00	0.00	0.00	0.00	0.00	0.00	0.00	0.00	0.00	0.00	0.00	0.00	0.00	0.00	0.00	0.00
0.55	0.26	0.01	0.00	0.00	0.00	0.00	0.00	0.00	0.00	0.00	0.00	0.00	0.00	0.00	0.00	0.00	0.00	0.00	0.00	0.00	0.00	0.00	0.00	0.00	0.00
0.6	0.24	0.01	0.00	0.00	0.00	0.00	0.00	0.00	0.00	0.00	0.00	0.00	0.00	0.00	0.00	0.00	0.00	0.00	0.00	0.00	0.00	0.00	0.00	0.00	0.00
0.65	0.21	0.00	0.00	0.00	0.00	0.00	0.00	0.00	0.00	0.00	0.00	0.00	0.00	0.00	0.00	0.00	0.00	0.00	0.00	0.00	0.00	0.00	0.00	0.00	0.00
0.70	0.18	0.00	0.00	0.00	0.00	0.00	0.00	0.00	0.00	0.00	0.00	0.00	0.00	0.00	0.00	0.00	0.00	0.00	0.00	0.00	0.00	0.00	0.00	0.00	0.00
0.75	0.15	0.00	0.00	0.00	0.00	0.00	0.00	0.00	0.00	0.00	0.00	0.00	0.00	0.00	0.00	0.00	0.00	0.00	0.00	0.00	0.00	0.00	0.00	0.00	0.00
0.80	0.15	0.00	0.00	0.00	0.00	0.00	0.00	0.00	0.00	0.00	0.00	0.00	0.00	0.00	0.00	0.00	0.00	0.00	0.00	0.00	0.00	0.00	0.00	0.00	0.00
0.85	0.15	0.00	0.00	0.00	0.00	0.00	0.00	0.00	0.00	0.00	0.00	0.00	0.00	0.00	0.00	0.00	0.00	0.00	0.00	0.00	0.00	0.00	0.00	0.00	0.01
0.90	0.15	0.00	0.00	0.00	0.00	0.00	0.00	0.00	0.00	0.00	0.00	0.00	0.00	0.00	0.00	0.00	0.00	0.00	0.00	0.00	0.00	0.00	0.00	0.00	0.04
0.92	0.15	0.00	0.00	0.00	0.00	0.00	0.00	0.00	0.00	0.00	0.00	0.00	0.00	0.00	0.00	0.00	0.00	0.00	0.00	0.00	0.00	0.00	0.00	0.01	0.09
0.94	0.15	0.00	0.00	0.00	0.00	0.00	0.00	0.00	0.00	0.00	0.00	0.00	0.00	0.00	0.00	0.00	0.00	0.00	0.00	0.00	0.00	0.00	0.00	0.01	0.16
0.96	0.15	0.00	0.00	0.00	0.00	0.00	0.00	0.00	0.00	0.00	0.00	0.00	0.00	0.00	0.00	0.00	0.00	0.00	0.00	0.00	0.00	0.00	0.00	0.01	0.26
0.98	0.15	0.00	0.00	0.00	0.00	0.00	0.00	0.00	0.00	0.00	0.00	0.00	0.00	0.00	0.00	0.00	0.00	0.00	0.00	0.00	0.00	0.00	0.00	0.02	0.33

(g) n_{xrf} = 300, n_{yrf} = 300

0.02	0.04	0.06	0.08	0.1	0.15	0.2	0.25	0.30	0.35	0.40	0.45	0.50	0.55	0.6	0.65	0.70	0.75	0.80	0.85	0.90	0.92	0.94	0.96	0.98	
0.91	0.56	0.37	0.30	0.28	0.28	0.27	0.18	0.15	0.15	0.15	0.15	0.15	0.15	0.15	0.15	0.15	0.15	0.14	0.15	0.15	0.15	0.15	0.15	0.15	0.02
0.56	0.07	0.02	0.01	0.01	0.01	0.01	0.00	0.00	0.00	0.00	0.00	0.00	0.00	0.00	0.00	0.00	0.00	0.00	0.00	0.00	0.00	0.00	0.00	0.00	0.04
0.36	0.02	0.00	0.00	0.00	0.00	0.00	0.00	0.00	0.00	0.00	0.00	0.00	0.00	0.00	0.00	0.00	0.00	0.00	0.00	0.00	0.00	0.00	0.00	0.00	0.06
0.29	0.01	0.00	0.00	0.00	0.00	0.00	0.00	0.00	0.00	0.00	0.00	0.00	0.00	0.00	0.00	0.00	0.00	0.00	0.00	0.00	0.00	0.00	0.00	0.00	0.08
0.27	0.01	0.00	0.00	0.00	0.00	0.00	0.00	0.00	0.00	0.00	0.00	0.00	0.00	0.00	0.00	0.00	0.00	0.00	0.00	0.00	0.00	0.00	0.00	0.00	0.1
0.27	0.01	0.00	0.00	0.00	0.00	0.00	0.00	0.00	0.00	0.00	0.00	0.00	0.00	0.00	0.00	0.00	0.00	0.00	0.00	0.00	0.00	0.00	0.00	0.00	0.15
0.26	0.01	0.00	0.00	0.00	0.00	0.00	0.00	0.00	0.00	0.00	0.00	0.00	0.00	0.00	0.00	0.00	0.00	0.00	0.00	0.00	0.00	0.00	0.00	0.00	0.2
0.18	0.00	0.00	0.00	0.00	0.00	0.00	0.00	0.00	0.00	0.00	0.00	0.00	0.00	0.00	0.00	0.00	0.00	0.00	0.00	0.00	0.00	0.00	0.00	0.00	0.25
0.15	0.00	0.00	0.00	0.00	0.00	0.00	0.00	0.00	0.00	0.00	0.00	0.00	0.00	0.00	0.00	0.00	0.00	0.00	0.00	0.00	0.00	0.00	0.00	0.00	0.30
0.15	0.00	0.00	0.00	0.00	0.00	0.00	0.00	0.00	0.00	0.00	0.00	0.00	0.00	0.00	0.00	0.00	0.00	0.00	0.00	0.00	0.00	0.00	0.00	0.00	0.35
0.15	0.00	0.00	0.00	0.00	0.00	0.00	0.00	0.00	0.00	0.00	0.00	0.00	0.00	0.00	0.00	0.00	0.00	0.00	0.00	0.00	0.00	0.00	0.00	0.00	0.40
0.15	0.00	0.00	0.00	0.00	0.00	0.00	0.00	0.00	0.00	0.00	0.00	0.00	0.00	0.00	0.00	0.00	0.00	0.00	0.00	0.00	0.00	0.00	0.00	0.00	0.45
0.15	0.00	0.00	0.00	0.00	0.00	0.00	0.00	0.00	0.00	0.00	0.00	0.00	0.00	0.00	0.00	0.00	0.00	0.00	0.00	0.00	0.00	0.00	0.00	0.00	0.50
0.15	0.00	0.00	0.00	0.00	0.00	0.00	0.00	0.00	0.00	0.00	0.00	0.00	0.00	0.00	0.00	0.00	0.00	0.00	0.00	0.00	0.00	0.00	0.00	0.00	0.55
0.15	0.00	0.00	0.00	0.00	0.00	0.00	0.00	0.00	0.00	0.00	0.00	0.00	0.00	0.00	0.00	0.00	0.00	0.00	0.00	0.00	0.00	0.00	0.00	0.00	0.6
0.15	0.00	0.00	0.00	0.00	0.00	0.00	0.00	0.00	0.00	0.00	0.00	0.00	0.00	0.00	0.00	0.00	0.00	0.00	0.00	0.00	0.00	0.00	0.00	0.00	0.65
0.15	0.00	0.00	0.00	0.00	0.00	0.00	0.00	0.00	0.00	0.00	0.00	0.00	0.00	0.00	0.00	0.00	0.00	0.00	0.00	0.00	0.00	0.00	0.00	0.00	0.70
0.15	0.00	0.00	0.00	0.00	0.00	0.00	0.00	0.00	0.00	0.00	0.00	0.00	0.00	0.00	0.00	0.00	0.00	0.00	0.00	0.00	0.00	0.00	0.00	0.00	0.75
0.15	0.00	0.00	0.00	0.00	0.00	0.00	0.00	0.00	0.00	0.00	0.00	0.00	0.00	0.00	0.00	0.00	0.00	0.00	0.00	0.00	0.00	0.00	0.00	0.00	0.80
0.15	0.00	0.00	0.00	0.00	0.00	0.00	0.00	0.00	0.00	0.00	0.00	0.00	0.00	0.00	0.00	0.00	0.00	0.00	0.00	0.00	0.00	0.00	0.00	0.00	0.85
0.15	0.00	0.00	0.00	0.00	0.00	0.00	0.00	0.00	0.00	0.00	0.00	0.00	0.00	0.00	0.00	0.00	0.00	0.00	0.00	0.00	0.00	0.00	0.00	0.00	0.90
0.15	0.00	0.00	0.00	0.00	0.00	0.00	0.00	0.00	0.00	0.00	0.00	0.00	0.00	0.00	0.00	0.00	0.00	0.00	0.00	0.00	0.00	0.00	0.00	0.00	0.92
0.15	0.00	0.00	0.00	0.00	0.00	0.00	0.00	0.00	0.00	0.00	0.00	0.00	0.00	0.00	0.00	0.00	0.00	0.00	0.00	0.00	0.00	0.00	0.00	0.00	0.94
0.15	0.00	0.00	0.00	0.00	0.00	0.00	0.00	0.00	0.00	0.00	0.00	0.00	0.00	0.00	0.00	0.00	0.00	0.00	0.00	0.00	0.00	0.00	0.00	0.00	0.96
0.15	0.00	0.00	0.00	0.00	0.00	0.00	0.00	0.00	0.00	0.00	0.00	0.00	0.00	0.00	0.00	0.00	0.00	0.00	0.00	0.00	0.00	0.00	0.00	0.00	0.98

Colour scale: 1, 0.8, 0.6, 0.4, 0.2, 0

(h) n_{xrf} = 400, n_{yrf} = 50

0.02	0.04	0.06	0.08	0.1	0.15	0.2	0.25	0.30	0.35	0.40	0.45	0.50	0.55	0.6	0.65	0.70	0.75	0.80	0.85	0.90	0.92	0.94	0.96	0.98	
1.00	1.00	1.00	0.99	0.98	0.97	0.97	0.97	0.97	0.97	0.97	0.97	0.97	0.97	0.97	0.97	0.97	0.97	0.97	0.97	0.97	0.97	0.97	0.97	0.97	0.02
0.99	0.96	0.94	0.92	0.87	0.84	0.85	0.85	0.85	0.85	0.84	0.84	0.84	0.84	0.84	0.85	0.85	0.85	0.85	0.84	0.84	0.84	0.84	0.84	0.84	0.04
0.97	0.86	0.81	0.78	0.69	0.63	0.63	0.64	0.63	0.63	0.63	0.63	0.63	0.63	0.63	0.63	0.63	0.64	0.63	0.63	0.64	0.64	0.63	0.63	0.63	0.06
0.91	0.69	0.63	0.59	0.48	0.42	0.41	0.42	0.42	0.42	0.42	0.41	0.41	0.41	0.42	0.42	0.42	0.42	0.41	0.42	0.42	0.43	0.42	0.42	0.42	0.08
0.82	0.50	0.43	0.40	0.30	0.24	0.24	0.24	0.25	0.24	0.24	0.24	0.24	0.24	0.24	0.24	0.25	0.24	0.24	0.24	0.24	0.25	0.25	0.25	0.25	0.1
0.54	0.16	0.12	0.10	0.06	0.05	0.05	0.05	0.05	0.05	0.05	0.05	0.05	0.05	0.05	0.05	0.05	0.05	0.05	0.05	0.04	0.05	0.05	0.05	0.05	0.15
0.31	0.03	0.02	0.02	0.01	0.01	0.01	0.01	0.01	0.01	0.01	0.01	0.01	0.01	0.01	0.01	0.01	0.01	0.01	0.01	0.01	0.01	0.01	0.01	0.01	0.2
0.19	0.01	0.00	0.00	0.00	0.00	0.00	0.00	0.00	0.00	0.00	0.00	0.00	0.00	0.00	0.00	0.00	0.00	0.00	0.00	0.00	0.00	0.00	0.00	0.00	0.25
0.13	0.00	0.00	0.00	0.00	0.00	0.00	0.00	0.00	0.00	0.00	0.00	0.00	0.00	0.00	0.00	0.00	0.00	0.00	0.00	0.00	0.00	0.00	0.00	0.00	0.30
0.11	0.00	0.00	0.00	0.00	0.00	0.00	0.00	0.00	0.00	0.00	0.00	0.00	0.00	0.00	0.00	0.00	0.00	0.00	0.00	0.00	0.00	0.00	0.00	0.00	0.35
0.10	0.00	0.00	0.00	0.00	0.00	0.00	0.00	0.00	0.00	0.00	0.00	0.00	0.00	0.00	0.00	0.00	0.00	0.00	0.00	0.00	0.00	0.00	0.00	0.00	0.40
0.10	0.00	0.00	0.00	0.00	0.00	0.00	0.00	0.00	0.00	0.00	0.00	0.00	0.00	0.00	0.00	0.00	0.00	0.00	0.00	0.00	0.00	0.00	0.00	0.00	0.45
0.09	0.00	0.00	0.00	0.00	0.00	0.00	0.00	0.00	0.00	0.00	0.00	0.00	0.00	0.00	0.00	0.00	0.00	0.00	0.00	0.00	0.00	0.00	0.00	0.00	0.50
0.09	0.00	0.00	0.00	0.00	0.00	0.00	0.00	0.00	0.00	0.00	0.00	0.00	0.00	0.00	0.00	0.00	0.00	0.00	0.00	0.00	0.00	0.00	0.00	0.00	0.55
0.09	0.00	0.00	0.00	0.00	0.00	0.00	0.00	0.00	0.00	0.00	0.00	0.00	0.00	0.00	0.00	0.00	0.00	0.00	0.00	0.00	0.00	0.00	0.00	0.00	0.6
0.07	0.00	0.00	0.00	0.00	0.00	0.00	0.00	0.00	0.00	0.00	0.00	0.00	0.00	0.00	0.00	0.00	0.00	0.00	0.00	0.00	0.00	0.00	0.00	0.00	0.65
0.06	0.00	0.00	0.00	0.00	0.00	0.00	0.00	0.00	0.00	0.00	0.00	0.00	0.00	0.00	0.00	0.00	0.00	0.00	0.00	0.00	0.00	0.00	0.00	0.00	0.70
0.05	0.00	0.00	0.00	0.00	0.00	0.00	0.00	0.00	0.00	0.00	0.00	0.00	0.00	0.00	0.00	0.00	0.00	0.00	0.00	0.00	0.00	0.00	0.00	0.00	0.75
0.04	0.00	0.00	0.00	0.00	0.00	0.00	0.00	0.00	0.00	0.00	0.00	0.00	0.00	0.00	0.00	0.00	0.00	0.00	0.00	0.00	0.00	0.00	0.00	0.00	0.80
0.04	0.00	0.00	0.00	0.00	0.00	0.00	0.00	0.00	0.00	0.00	0.00	0.00	0.00	0.00	0.00	0.00	0.00	0.00	0.00	0.00	0.00	0.00	0.00	0.01	0.85
0.04	0.00	0.00	0.00	0.00	0.00	0.00	0.00	0.00	0.00	0.00	0.00	0.00	0.00	0.00	0.00	0.00	0.00	0.00	0.00	0.00	0.00	0.00	0.00	0.04	0.90
0.04	0.00	0.00	0.00	0.00	0.00	0.00	0.00	0.00	0.00	0.00	0.00	0.00	0.00	0.00	0.00	0.00	0.00	0.00	0.00	0.00	0.00	0.00	0.00	0.09	0.92
0.04	0.00	0.00	0.00	0.00	0.00	0.00	0.00	0.00	0.00	0.00	0.00	0.00	0.00	0.00	0.00	0.00	0.00	0.00	0.00	0.00	0.00	0.00	0.00	0.16	0.94
0.04	0.00	0.00	0.00	0.00	0.00	0.00	0.00	0.00	0.00	0.00	0.00	0.00	0.00	0.00	0.00	0.00	0.00	0.00	0.00	0.00	0.00	0.00	0.01	0.25	0.96
0.04	0.00	0.00	0.00	0.00	0.00	0.00	0.00	0.00	0.00	0.00	0.00	0.00	0.00	0.00	0.00	0.00	0.00	0.00	0.00	0.00	0.00	0.00	0.01	0.29	0.98

Colour scale: 1, 0.8, 0.6, 0.4, 0.2, 0

(i) n_{xrf} = 400, n_{yrf} = 400

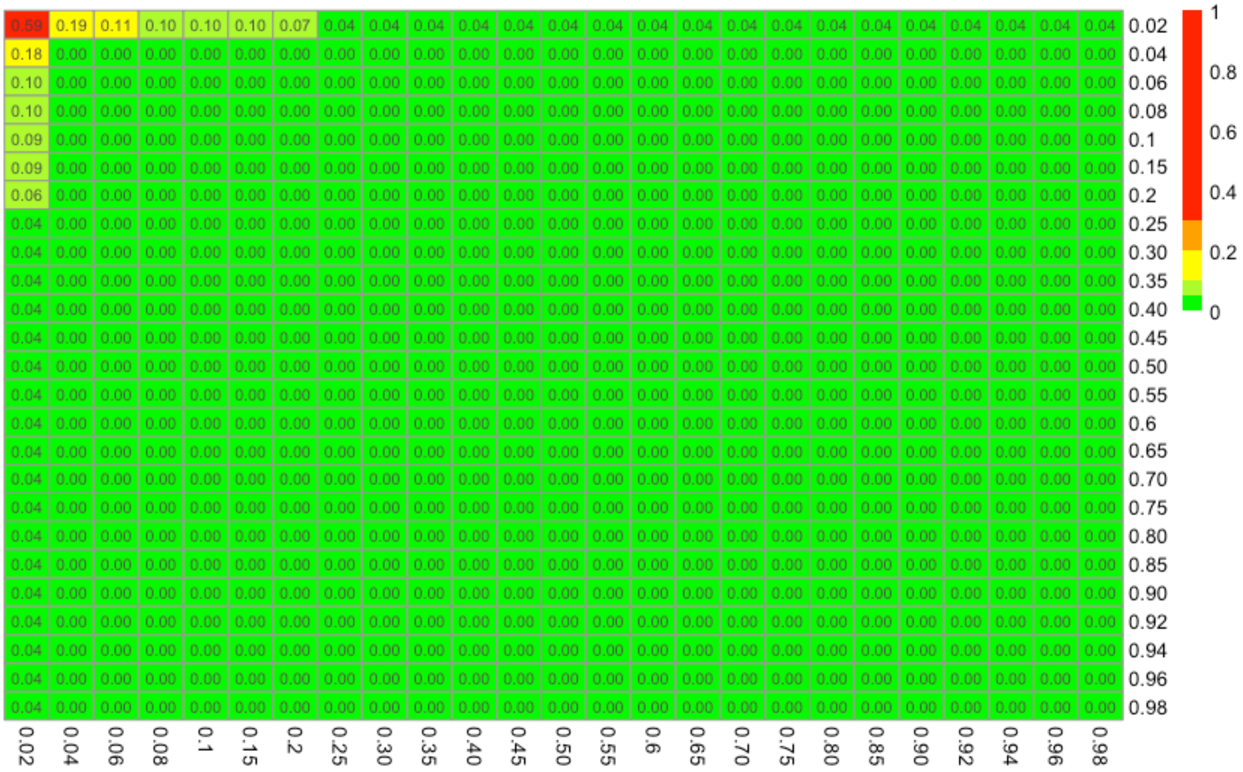

(j) n_{xrf} = 500, n_{yrf} = 50

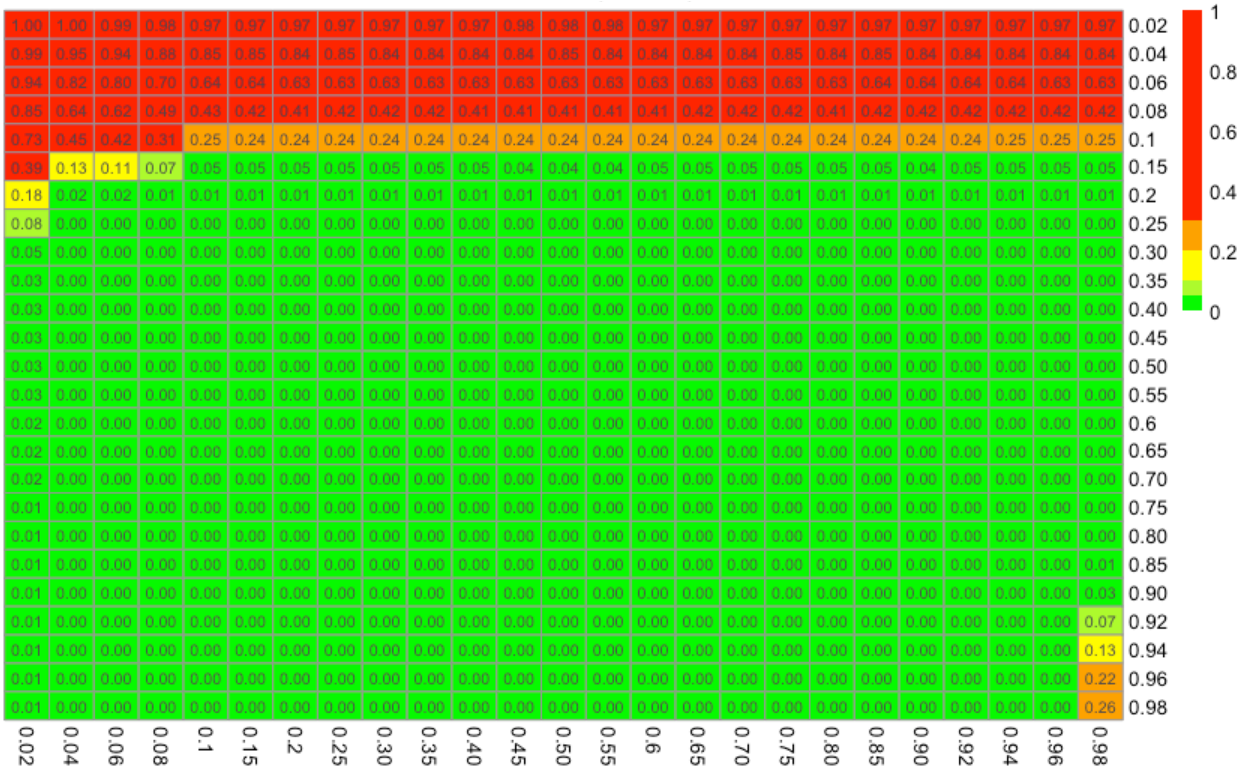

(k) n_{xrf} = 500, n_{yrf} = 500

0.02	0.04	0.06	0.08	0.1	0.15	0.2	0.25	0.30	0.35	0.40	0.45	0.50	0.55	0.6	0.65	0.70	0.75	0.80	0.85	0.90	0.92	0.94	0.96	0.98	
0.26	0.05	0.03	0.03	0.03	0.03	0.01	0.01	0.01	0.01	0.01	0.01	0.01	0.01	0.01	0.01	0.01	0.01	0.01	0.01	0.01	0.01	0.01	0.01	0.01	0.02
0.04	0.00	0.00	0.00	0.00	0.00	0.00	0.00	0.00	0.00	0.00	0.00	0.00	0.00	0.00	0.00	0.00	0.00	0.00	0.00	0.00	0.00	0.00	0.00	0.00	0.04
0.03	0.00	0.00	0.00	0.00	0.00	0.00	0.00	0.00	0.00	0.00	0.00	0.00	0.00	0.00	0.00	0.00	0.00	0.00	0.00	0.00	0.00	0.00	0.00	0.00	0.06
0.03	0.00	0.00	0.00	0.00	0.00	0.00	0.00	0.00	0.00	0.00	0.00	0.00	0.00	0.00	0.00	0.00	0.00	0.00	0.00	0.00	0.00	0.00	0.00	0.00	0.08
0.03	0.00	0.00	0.00	0.00	0.00	0.00	0.00	0.00	0.00	0.00	0.00	0.00	0.00	0.00	0.00	0.00	0.00	0.00	0.00	0.00	0.00	0.00	0.00	0.00	0.1
0.03	0.00	0.00	0.00	0.00	0.00	0.00	0.00	0.00	0.00	0.00	0.00	0.00	0.00	0.00	0.00	0.00	0.00	0.00	0.00	0.00	0.00	0.00	0.00	0.00	0.15
0.01	0.00	0.00	0.00	0.00	0.00	0.00	0.00	0.00	0.00	0.00	0.00	0.00	0.00	0.00	0.00	0.00	0.00	0.00	0.00	0.00	0.00	0.00	0.00	0.00	0.2
0.01	0.00	0.00	0.00	0.00	0.00	0.00	0.00	0.00	0.00	0.00	0.00	0.00	0.00	0.00	0.00	0.00	0.00	0.00	0.00	0.00	0.00	0.00	0.00	0.00	0.25
0.01	0.00	0.00	0.00	0.00	0.00	0.00	0.00	0.00	0.00	0.00	0.00	0.00	0.00	0.00	0.00	0.00	0.00	0.00	0.00	0.00	0.00	0.00	0.00	0.00	0.30
0.01	0.00	0.00	0.00	0.00	0.00	0.00	0.00	0.00	0.00	0.00	0.00	0.00	0.00	0.00	0.00	0.00	0.00	0.00	0.00	0.00	0.00	0.00	0.00	0.00	0.35
0.01	0.00	0.00	0.00	0.00	0.00	0.00	0.00	0.00	0.00	0.00	0.00	0.00	0.00	0.00	0.00	0.00	0.00	0.00	0.00	0.00	0.00	0.00	0.00	0.00	0.40
0.01	0.00	0.00	0.00	0.00	0.00	0.00	0.00	0.00	0.00	0.00	0.00	0.00	0.00	0.00	0.00	0.00	0.00	0.00	0.00	0.00	0.00	0.00	0.00	0.00	0.45
0.01	0.00	0.00	0.00	0.00	0.00	0.00	0.00	0.00	0.00	0.00	0.00	0.00	0.00	0.00	0.00	0.00	0.00	0.00	0.00	0.00	0.00	0.00	0.00	0.00	0.50
0.01	0.00	0.00	0.00	0.00	0.00	0.00	0.00	0.00	0.00	0.00	0.00	0.00	0.00	0.00	0.00	0.00	0.00	0.00	0.00	0.00	0.00	0.00	0.00	0.00	0.55
0.01	0.00	0.00	0.00	0.00	0.00	0.00	0.00	0.00	0.00	0.00	0.00	0.00	0.00	0.00	0.00	0.00	0.00	0.00	0.00	0.00	0.00	0.00	0.00	0.00	0.6
0.01	0.00	0.00	0.00	0.00	0.00	0.00	0.00	0.00	0.00	0.00	0.00	0.00	0.00	0.00	0.00	0.00	0.00	0.00	0.00	0.00	0.00	0.00	0.00	0.00	0.65
0.01	0.00	0.00	0.00	0.00	0.00	0.00	0.00	0.00	0.00	0.00	0.00	0.00	0.00	0.00	0.00	0.00	0.00	0.00	0.00	0.00	0.00	0.00	0.00	0.00	0.70
0.01	0.00	0.00	0.00	0.00	0.00	0.00	0.00	0.00	0.00	0.00	0.00	0.00	0.00	0.00	0.00	0.00	0.00	0.00	0.00	0.00	0.00	0.00	0.00	0.00	0.75
0.01	0.00	0.00	0.00	0.00	0.00	0.00	0.00	0.00	0.00	0.00	0.00	0.00	0.00	0.00	0.00	0.00	0.00	0.00	0.00	0.00	0.00	0.00	0.00	0.00	0.80
0.01	0.00	0.00	0.00	0.00	0.00	0.00	0.00	0.00	0.00	0.00	0.00	0.00	0.00	0.00	0.00	0.00	0.00	0.00	0.00	0.00	0.00	0.00	0.00	0.00	0.85
0.01	0.00	0.00	0.00	0.00	0.00	0.00	0.00	0.00	0.00	0.00	0.00	0.00	0.00	0.00	0.00	0.00	0.00	0.00	0.00	0.00	0.00	0.00	0.00	0.00	0.90
0.01	0.00	0.00	0.00	0.00	0.00	0.00	0.00	0.00	0.00	0.00	0.00	0.00	0.00	0.00	0.00	0.00	0.00	0.00	0.00	0.00	0.00	0.00	0.00	0.00	0.92
0.01	0.00	0.00	0.00	0.00	0.00	0.00	0.00	0.00	0.00	0.00	0.00	0.00	0.00	0.00	0.00	0.00	0.00	0.00	0.00	0.00	0.00	0.00	0.00	0.00	0.94
0.01	0.00	0.00	0.00	0.00	0.00	0.00	0.00	0.00	0.00	0.00	0.00	0.00	0.00	0.00	0.00	0.00	0.00	0.00	0.00	0.00	0.00	0.00	0.00	0.00	0.96
0.01	0.00	0.00	0.00	0.00	0.00	0.00	0.00	0.00	0.00	0.00	0.00	0.00	0.00	0.00	0.00	0.00	0.00	0.00	0.00	0.00	0.00	0.00	0.00	0.00	0.98

Colour scale: 1, 0.8, 0.6, 0.4, 0.2, 0

Appendix S4 Figure 5: Proportion of undefined confidence intervals constructed using the delta method

(a) $n_{xrf} = 50$, $n_{yrf} = 50$

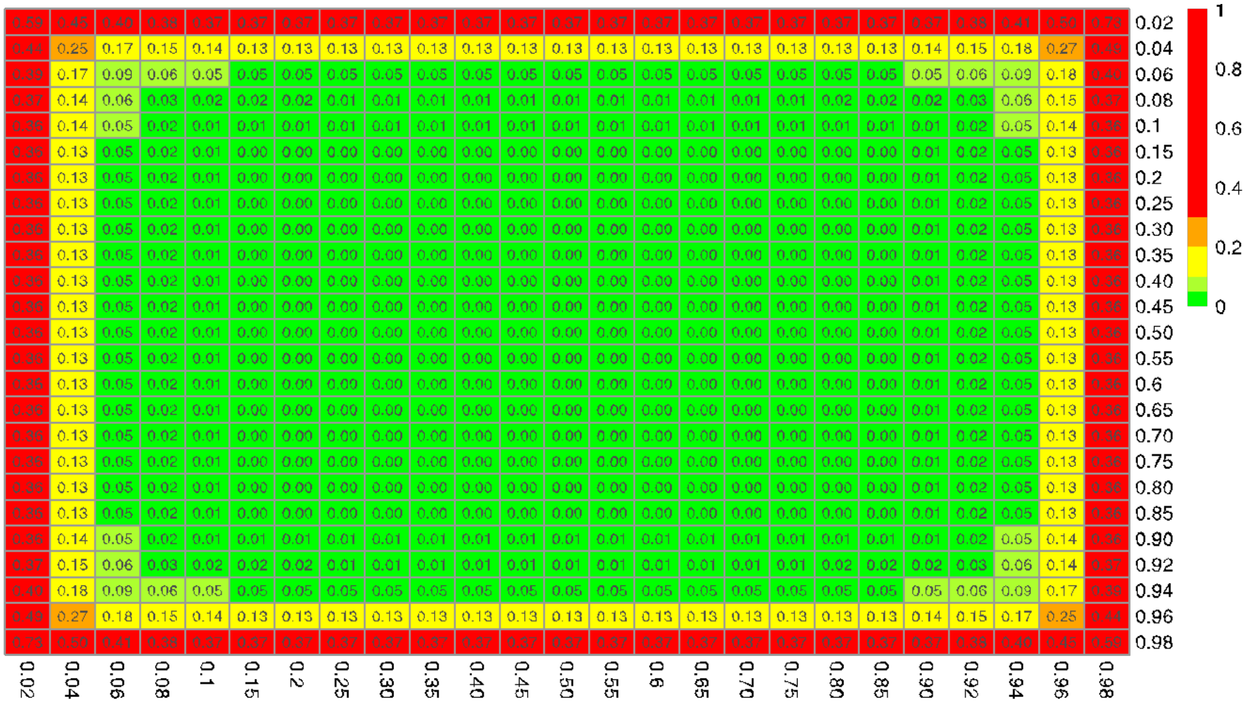

(b) $n_{xrf} = 100$, $n_{yrf} = 50$

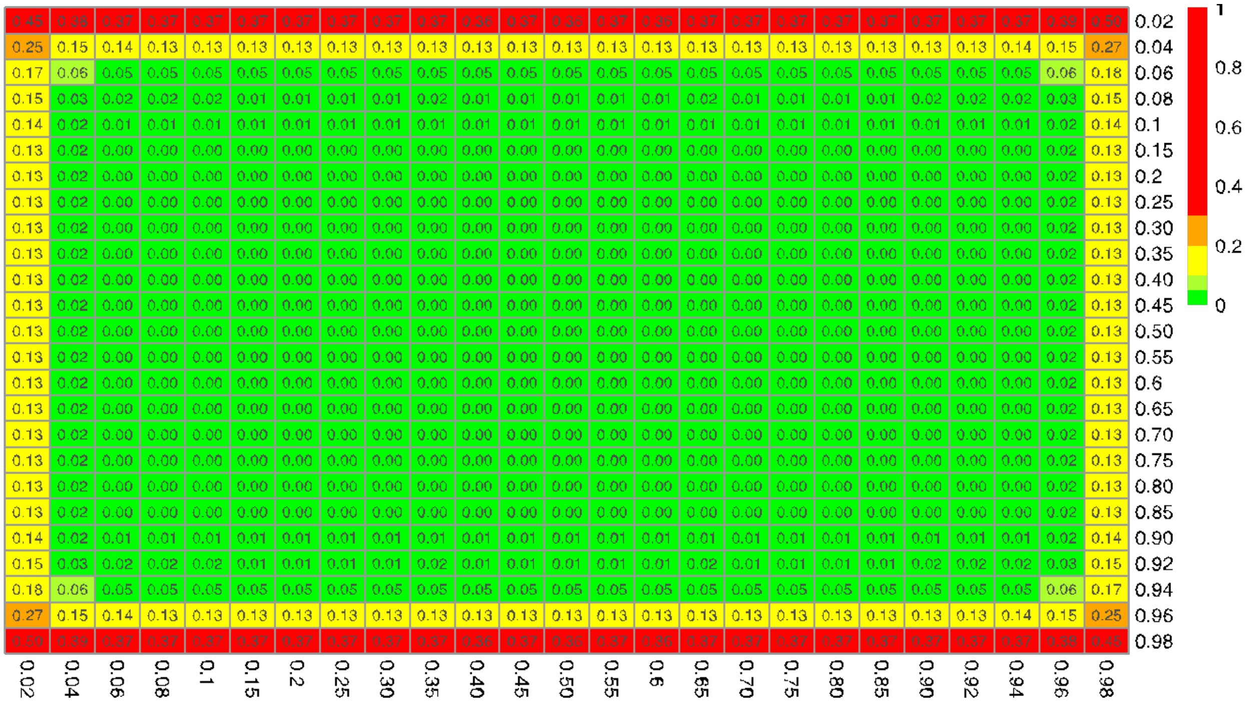

(c) n_{xrf} = 100, n_{yrf} = 100

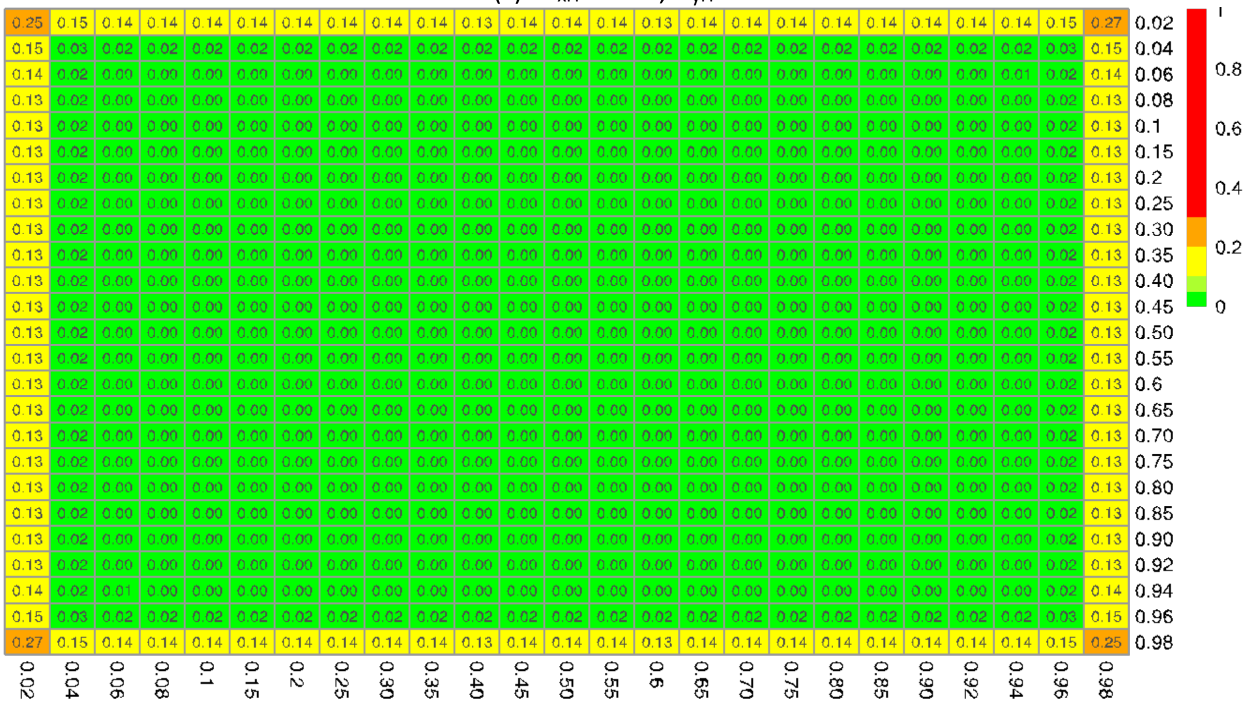

(d) n_{xrf} = 200, n_{yrf} = 50

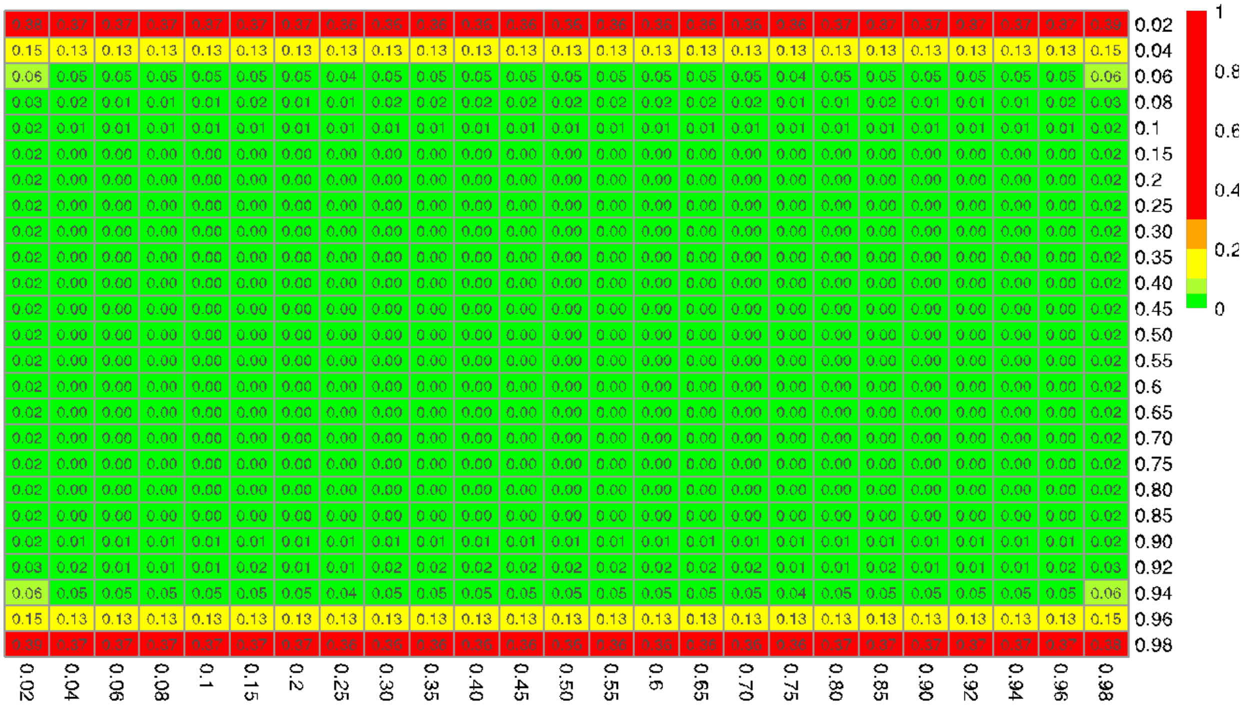

(e) n_{xrf} = 200, n_{yrf} = 200

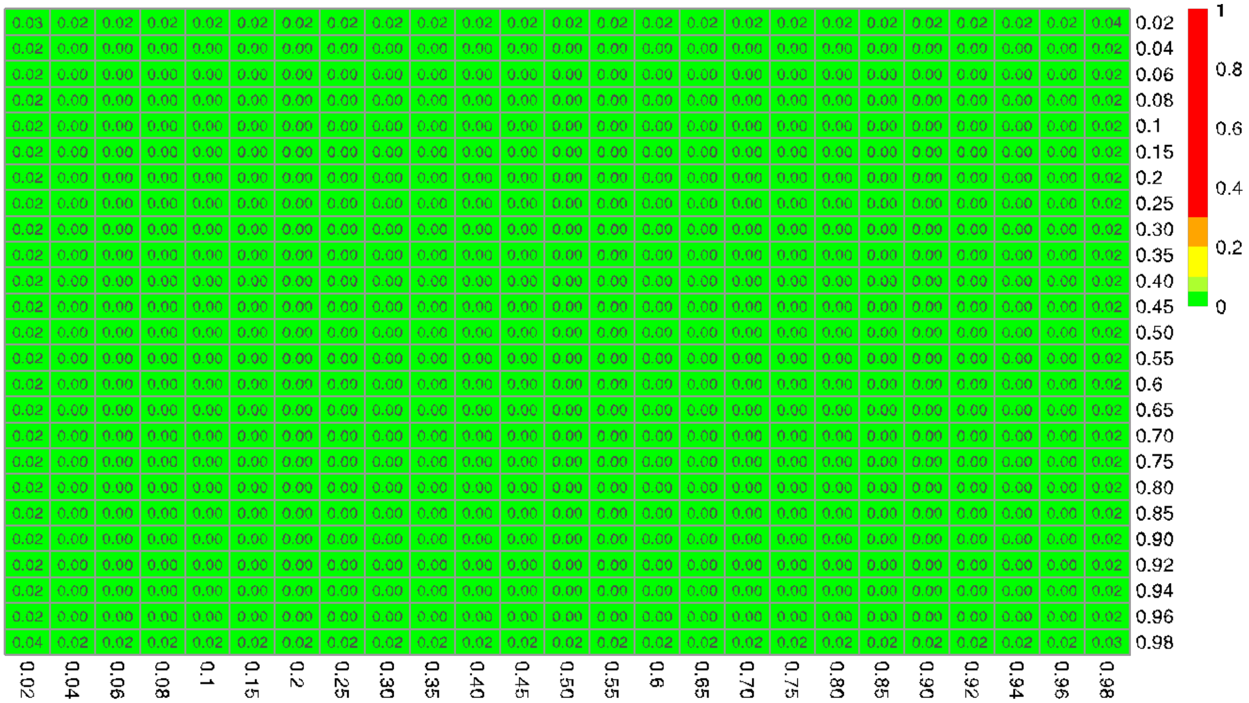

(f) n_{xrf} = 300, n_{yrf} = 50

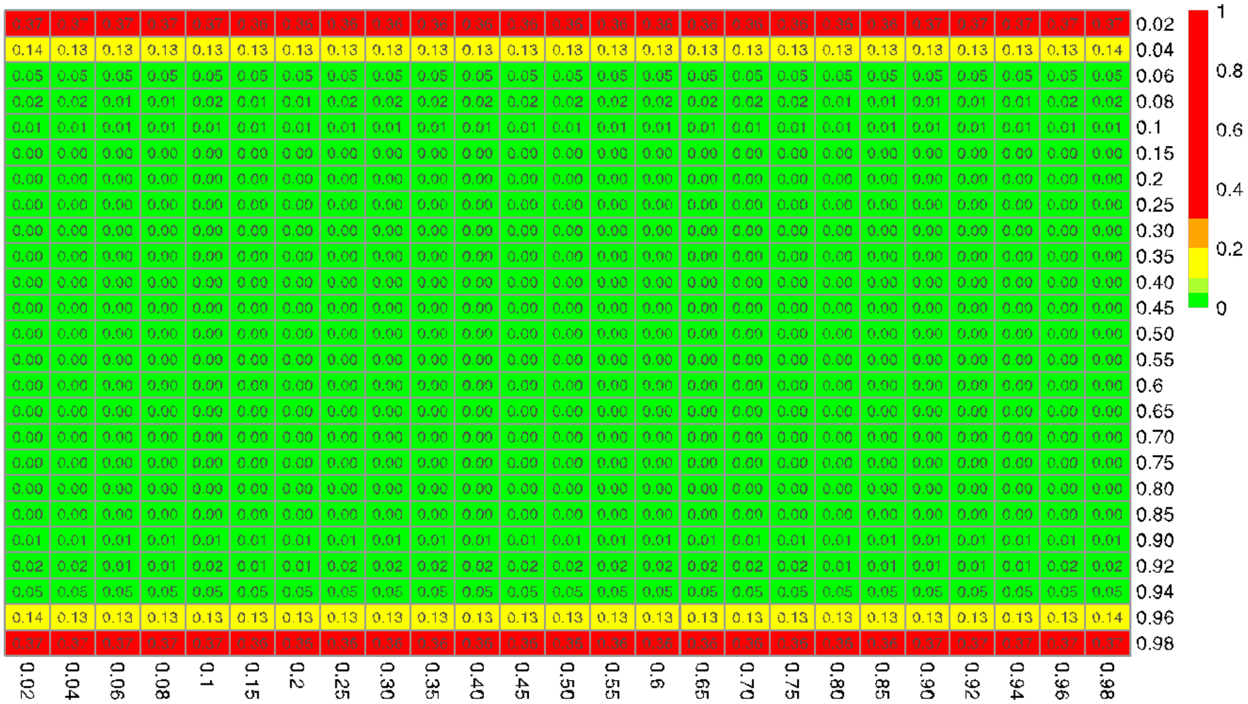

(g) $n_{xrf} = 300, n_{yrf} = 300$

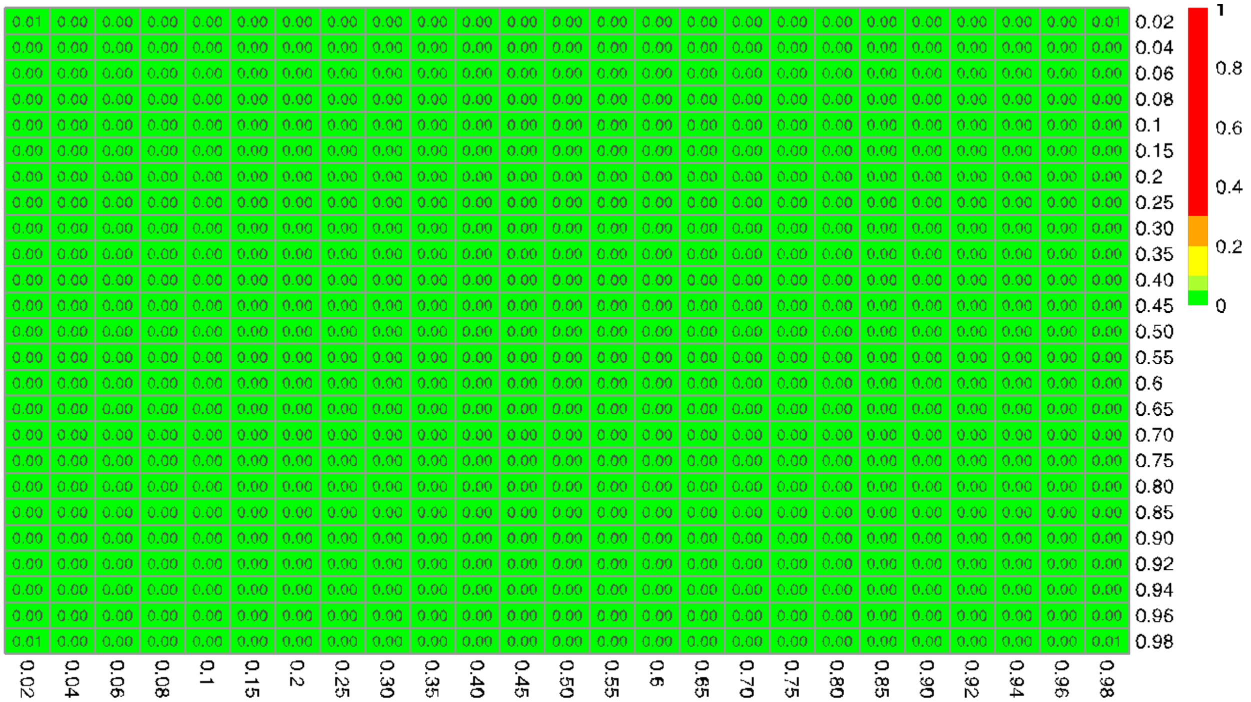

(h) $n_{xrf} = 400, n_{yrf} = 50$

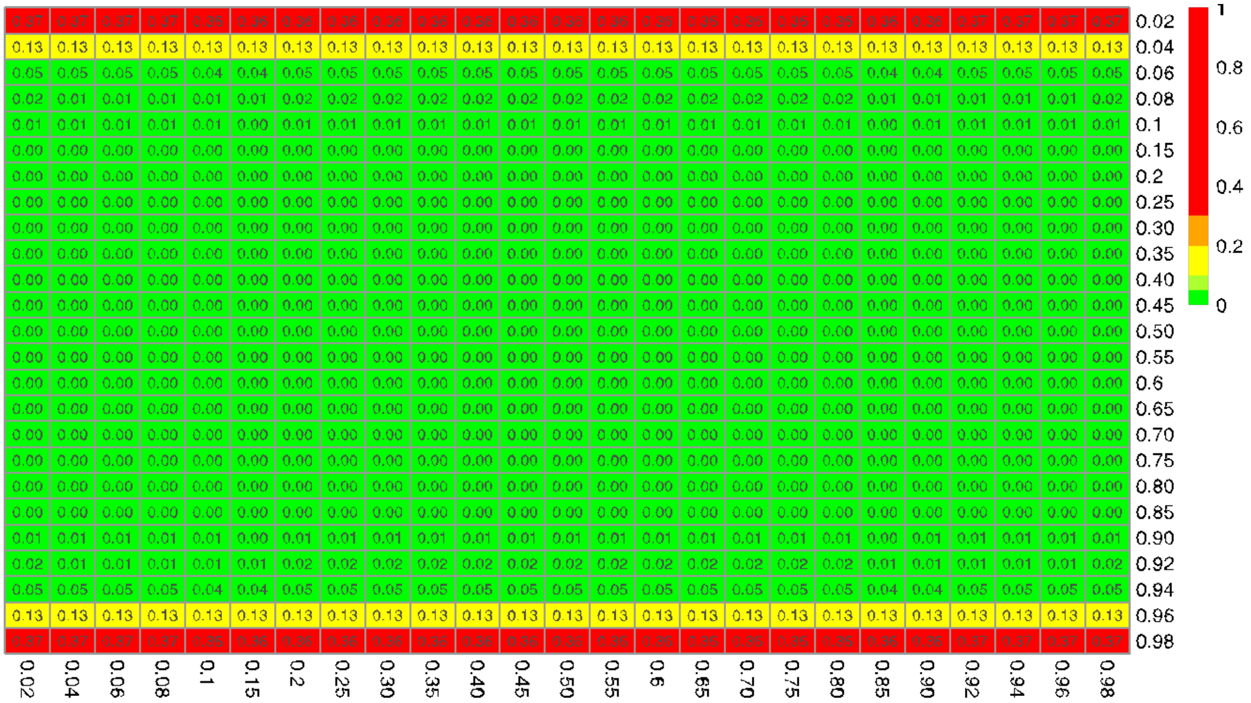

(i) n_{xrf} = 400, n_{yrf} = 400

	0.02	0.04	0.06	0.08	0.1	0.15	0.2	0.25	0.30	0.35	0.40	0.45	0.50	0.55	0.6	0.65	0.70	0.75	0.80	0.85	0.90	0.92	0.94	0.96	0.98
0.02	0.00	0.00	0.00	0.00	0.00	0.00	0.00	0.00	0.00	0.00	0.00	0.00	0.00	0.00	0.00	0.00	0.00	0.00	0.00	0.00	0.00	0.00	0.00	0.00	0.00
0.04	0.00	0.00	0.00	0.00	0.00	0.00	0.00	0.00	0.00	0.00	0.00	0.00	0.00	0.00	0.00	0.00	0.00	0.00	0.00	0.00	0.00	0.00	0.00	0.00	0.00
0.06	0.00	0.00	0.00	0.00	0.00	0.00	0.00	0.00	0.00	0.00	0.00	0.00	0.00	0.00	0.00	0.00	0.00	0.00	0.00	0.00	0.00	0.00	0.00	0.00	0.00
0.08	0.00	0.00	0.00	0.00	0.00	0.00	0.00	0.00	0.00	0.00	0.00	0.00	0.00	0.00	0.00	0.00	0.00	0.00	0.00	0.00	0.00	0.00	0.00	0.00	0.00
0.1	0.00	0.00	0.00	0.00	0.00	0.00	0.00	0.00	0.00	0.00	0.00	0.00	0.00	0.00	0.00	0.00	0.00	0.00	0.00	0.00	0.00	0.00	0.00	0.00	0.00
0.15	0.00	0.00	0.00	0.00	0.00	0.00	0.00	0.00	0.00	0.00	0.00	0.00	0.00	0.00	0.00	0.00	0.00	0.00	0.00	0.00	0.00	0.00	0.00	0.00	0.00
0.2	0.00	0.00	0.00	0.00	0.00	0.00	0.00	0.00	0.00	0.00	0.00	0.00	0.00	0.00	0.00	0.00	0.00	0.00	0.00	0.00	0.00	0.00	0.00	0.00	0.00
0.25	0.00	0.00	0.00	0.00	0.00	0.00	0.00	0.00	0.00	0.00	0.00	0.00	0.00	0.00	0.00	0.00	0.00	0.00	0.00	0.00	0.00	0.00	0.00	0.00	0.00
0.30	0.00	0.00	0.00	0.00	0.00	0.00	0.00	0.00	0.00	0.00	0.00	0.00	0.00	0.00	0.00	0.00	0.00	0.00	0.00	0.00	0.00	0.00	0.00	0.00	0.00
0.35	0.00	0.00	0.00	0.00	0.00	0.00	0.00	0.00	0.00	0.00	0.00	0.00	0.00	0.00	0.00	0.00	0.00	0.00	0.00	0.00	0.00	0.00	0.00	0.00	0.00
0.40	0.00	0.00	0.00	0.00	0.00	0.00	0.00	0.00	0.00	0.00	0.00	0.00	0.00	0.00	0.00	0.00	0.00	0.00	0.00	0.00	0.00	0.00	0.00	0.00	0.00
0.45	0.00	0.00	0.00	0.00	0.00	0.00	0.00	0.00	0.00	0.00	0.00	0.00	0.00	0.00	0.00	0.00	0.00	0.00	0.00	0.00	0.00	0.00	0.00	0.00	0.00
0.50	0.00	0.00	0.00	0.00	0.00	0.00	0.00	0.00	0.00	0.00	0.00	0.00	0.00	0.00	0.00	0.00	0.00	0.00	0.00	0.00	0.00	0.00	0.00	0.00	0.00
0.55	0.00	0.00	0.00	0.00	0.00	0.00	0.00	0.00	0.00	0.00	0.00	0.00	0.00	0.00	0.00	0.00	0.00	0.00	0.00	0.00	0.00	0.00	0.00	0.00	0.00
0.6	0.00	0.00	0.00	0.00	0.00	0.00	0.00	0.00	0.00	0.00	0.00	0.00	0.00	0.00	0.00	0.00	0.00	0.00	0.00	0.00	0.00	0.00	0.00	0.00	0.00
0.65	0.00	0.00	0.00	0.00	0.00	0.00	0.00	0.00	0.00	0.00	0.00	0.00	0.00	0.00	0.00	0.00	0.00	0.00	0.00	0.00	0.00	0.00	0.00	0.00	0.00
0.70	0.00	0.00	0.00	0.00	0.00	0.00	0.00	0.00	0.00	0.00	0.00	0.00	0.00	0.00	0.00	0.00	0.00	0.00	0.00	0.00	0.00	0.00	0.00	0.00	0.00
0.75	0.00	0.00	0.00	0.00	0.00	0.00	0.00	0.00	0.00	0.00	0.00	0.00	0.00	0.00	0.00	0.00	0.00	0.00	0.00	0.00	0.00	0.00	0.00	0.00	0.00
0.80	0.00	0.00	0.00	0.00	0.00	0.00	0.00	0.00	0.00	0.00	0.00	0.00	0.00	0.00	0.00	0.00	0.00	0.00	0.00	0.00	0.00	0.00	0.00	0.00	0.00
0.85	0.00	0.00	0.00	0.00	0.00	0.00	0.00	0.00	0.00	0.00	0.00	0.00	0.00	0.00	0.00	0.00	0.00	0.00	0.00	0.00	0.00	0.00	0.00	0.00	0.00
0.90	0.00	0.00	0.00	0.00	0.00	0.00	0.00	0.00	0.00	0.00	0.00	0.00	0.00	0.00	0.00	0.00	0.00	0.00	0.00	0.00	0.00	0.00	0.00	0.00	0.00
0.92	0.00	0.00	0.00	0.00	0.00	0.00	0.00	0.00	0.00	0.00	0.00	0.00	0.00	0.00	0.00	0.00	0.00	0.00	0.00	0.00	0.00	0.00	0.00	0.00	0.00
0.94	0.00	0.00	0.00	0.00	0.00	0.00	0.00	0.00	0.00	0.00	0.00	0.00	0.00	0.00	0.00	0.00	0.00	0.00	0.00	0.00	0.00	0.00	0.00	0.00	0.00
0.96	0.00	0.00	0.00	0.00	0.00	0.00	0.00	0.00	0.00	0.00	0.00	0.00	0.00	0.00	0.00	0.00	0.00	0.00	0.00	0.00	0.00	0.00	0.00	0.00	0.00
0.98	0.00	0.00	0.00	0.00	0.00	0.00	0.00	0.00	0.00	0.00	0.00	0.00	0.00	0.00	0.00	0.00	0.00	0.00	0.00	0.00	0.00	0.00	0.00	0.00	0.00

(j) n_{xrf} = 500, n_{yrf} = 50

	0.02	0.04	0.06	0.08	0.1	0.15	0.2	0.25	0.30	0.35	0.40	0.45	0.50	0.55	0.6	0.65	0.70	0.75	0.80	0.85	0.90	0.92	0.94	0.96	0.98
0.02	0.37	0.37	0.37	0.36	0.36	0.36	0.36	0.36	0.36	0.35	0.36	0.36	0.36	0.36	0.35	0.35	0.36	0.36	0.36	0.36	0.36	0.36	0.37	0.37	0.37
0.04	0.13	0.13	0.13	0.13	0.13	0.13	0.13	0.13	0.13	0.13	0.13	0.13	0.13	0.13	0.13	0.13	0.13	0.13	0.13	0.13	0.13	0.13	0.13	0.13	0.13
0.06	0.05	0.05	0.05	0.04	0.04	0.05	0.05	0.05	0.05	0.05	0.04	0.04	0.04	0.04	0.04	0.05	0.05	0.05	0.05	0.05	0.04	0.04	0.05	0.05	0.05
0.08	0.02	0.01	0.02	0.01	0.01	0.01	0.01	0.02	0.01	0.02	0.01	0.02	0.02	0.02	0.01	0.02	0.01	0.02	0.01	0.01	0.01	0.01	0.02	0.01	0.02
0.1	0.01	0.01	0.01	0.01	0.01	0.01	0.01	0.01	0.01	0.01	0.01	0.01	0.01	0.01	0.01	0.01	0.01	0.01	0.01	0.01	0.01	0.01	0.01	0.01	0.01
0.15	0.00	0.00	0.00	0.00	0.00	0.00	0.00	0.00	0.00	0.00	0.00	0.00	0.00	0.00	0.00	0.00	0.00	0.00	0.00	0.00	0.00	0.00	0.00	0.00	0.00
0.2	0.00	0.00	0.00	0.00	0.00	0.00	0.00	0.00	0.00	0.00	0.00	0.00	0.00	0.00	0.00	0.00	0.00	0.00	0.00	0.00	0.00	0.00	0.00	0.00	0.00
0.25	0.00	0.00	0.00	0.00	0.00	0.00	0.00	0.00	0.00	0.00	0.00	0.00	0.00	0.00	0.00	0.00	0.00	0.00	0.00	0.00	0.00	0.00	0.00	0.00	0.00
0.30	0.00	0.00	0.00	0.00	0.00	0.00	0.00	0.00	0.00	0.00	0.00	0.00	0.00	0.00	0.00	0.00	0.00	0.00	0.00	0.00	0.00	0.00	0.00	0.00	0.00
0.35	0.00	0.00	0.00	0.00	0.00	0.00	0.00	0.00	0.00	0.00	0.00	0.00	0.00	0.00	0.00	0.00	0.00	0.00	0.00	0.00	0.00	0.00	0.00	0.00	0.00
0.40	0.00	0.00	0.00	0.00	0.00	0.00	0.00	0.00	0.00	0.00	0.00	0.00	0.00	0.00	0.00	0.00	0.00	0.00	0.00	0.00	0.00	0.00	0.00	0.00	0.00
0.45	0.00	0.00	0.00	0.00	0.00	0.00	0.00	0.00	0.00	0.00	0.00	0.00	0.00	0.00	0.00	0.00	0.00	0.00	0.00	0.00	0.00	0.00	0.00	0.00	0.00
0.50	0.00	0.00	0.00	0.00	0.00	0.00	0.00	0.00	0.00	0.00	0.00	0.00	0.00	0.00	0.00	0.00	0.00	0.00	0.00	0.00	0.00	0.00	0.00	0.00	0.00
0.55	0.00	0.00	0.00	0.00	0.00	0.00	0.00	0.00	0.00	0.00	0.00	0.00	0.00	0.00	0.00	0.00	0.00	0.00	0.00	0.00	0.00	0.00	0.00	0.00	0.00
0.6	0.00	0.00	0.00	0.00	0.00	0.00	0.00	0.00	0.00	0.00	0.00	0.00	0.00	0.00	0.00	0.00	0.00	0.00	0.00	0.00	0.00	0.00	0.00	0.00	0.00
0.65	0.00	0.00	0.00	0.00	0.00	0.00	0.00	0.00	0.00	0.00	0.00	0.00	0.00	0.00	0.00	0.00	0.00	0.00	0.00	0.00	0.00	0.00	0.00	0.00	0.00
0.70	0.00	0.00	0.00	0.00	0.00	0.00	0.00	0.00	0.00	0.00	0.00	0.00	0.00	0.00	0.00	0.00	0.00	0.00	0.00	0.00	0.00	0.00	0.00	0.00	0.00
0.75	0.00	0.00	0.00	0.00	0.00	0.00	0.00	0.00	0.00	0.00	0.00	0.00	0.00	0.00	0.00	0.00	0.00	0.00	0.00	0.00	0.00	0.00	0.00	0.00	0.00
0.80	0.00	0.00	0.00	0.00	0.00	0.00	0.00	0.00	0.00	0.00	0.00	0.00	0.00	0.00	0.00	0.00	0.00	0.00	0.00	0.00	0.00	0.00	0.00	0.00	0.00
0.85	0.00	0.00	0.00	0.00	0.00	0.00	0.00	0.00	0.00	0.00	0.00	0.00	0.00	0.00	0.00	0.00	0.00	0.00	0.00	0.00	0.00	0.00	0.00	0.00	0.00
0.90	0.01	0.01	0.01	0.01	0.01	0.01	0.01	0.01	0.01	0.01	0.01	0.01	0.01	0.01	0.01	0.01	0.01	0.01	0.01	0.01	0.01	0.01	0.01	0.01	0.01
0.92	0.02	0.01	0.02	0.01	0.01	0.01	0.01	0.02	0.01	0.02	0.01	0.02	0.02	0.02	0.01	0.02	0.01	0.02	0.01	0.01	0.01	0.01	0.02	0.01	0.02
0.94	0.05	0.05	0.05	0.04	0.04	0.05	0.05	0.05	0.05	0.05	0.04	0.04	0.04	0.04	0.04	0.05	0.05	0.05	0.05	0.05	0.04	0.04	0.05	0.05	0.05
0.96	0.13	0.13	0.13	0.13	0.13	0.13	0.13	0.13	0.13	0.13	0.13	0.13	0.13	0.13	0.13	0.13	0.13	0.13	0.13	0.13	0.13	0.13	0.13	0.13	0.13
0.98	0.37	0.37	0.37	0.36	0.36	0.36	0.36	0.36	0.36	0.35	0.36	0.36	0.36	0.36	0.35	0.35	0.36	0.36	0.36	0.36	0.36	0.36	0.37	0.37	0.37

(k) n_{xrf} = 500, n_{yrf} = 500

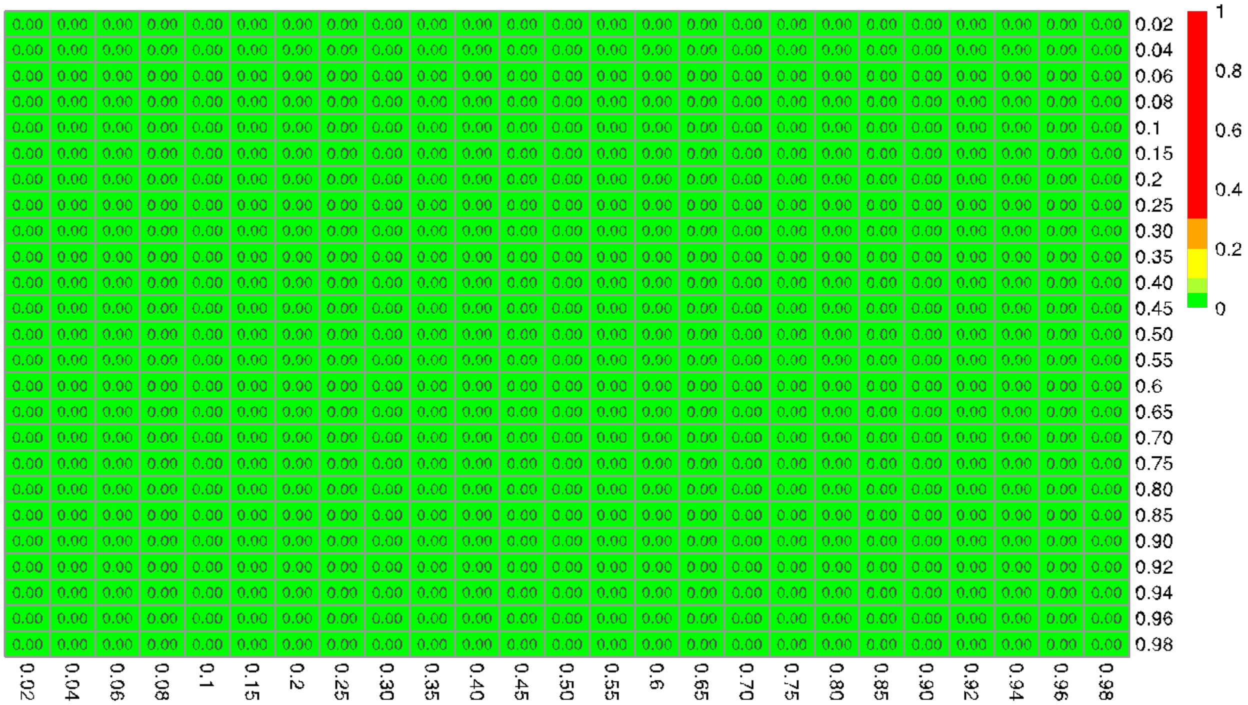

Appendix S4 Figure 6: Estimated 95% coverage probabilities for the confidence intervals constructed using the exact method, without removing the degenerate intervals.

(a) n_{xrf} = 50, n_{yrf} = 50

	0.02	0.04	0.06	0.08	0.1	0.15	0.2	0.25	0.30	0.35	0.40	0.45	0.50	0.55	0.6	0.65	0.70	0.75	0.80	0.85	0.90	0.92	0.94	0.96	0.98
0.02	0.41	0.55	0.60	0.62	0.62	0.63	0.63	0.63	0.63	0.63	0.63	0.63	0.63	0.63	0.63	0.63	0.63	0.63	0.63	0.63	0.63	0.63	0.63	0.63	0.63
0.04	0.56	0.75	0.79	0.78	0.83	0.83	0.84	0.85	0.86	0.86	0.86	0.86	0.86	0.86	0.86	0.86	0.86	0.86	0.86	0.86	0.86	0.86	0.86	0.86	0.86
0.06	0.61	0.79	0.83	0.85	0.85	0.85	0.88	0.87	0.89	0.88	0.87	0.88	0.87	0.88	0.87	0.88	0.87	0.86	0.87	0.86	0.86	0.86	0.86	0.86	0.86
0.08	0.63	0.78	0.85	0.87	0.86	0.89	0.88	0.89	0.88	0.89	0.89	0.89	0.90	0.90	0.90	0.90	0.90	0.91	0.91	0.91	0.91	0.91	0.91	0.91	0.91
0.1	0.63	0.83	0.86	0.86	0.87	0.88	0.89	0.89	0.89	0.89	0.90	0.90	0.90	0.90	0.90	0.90	0.90	0.90	0.89	0.89	0.89	0.88	0.88	0.88	0.88
0.15	0.64	0.83	0.85	0.89	0.88	0.88	0.90	0.91	0.91	0.91	0.92	0.92	0.92	0.92	0.92	0.93	0.92	0.93	0.92	0.93	0.93	0.93	0.93	0.93	0.94
0.2	0.64	0.84	0.88	0.89	0.89	0.90	0.91	0.92	0.92	0.93	0.92	0.93	0.93	0.92	0.93	0.93	0.93	0.93	0.93	0.93	0.93	0.93	0.93	0.94	0.94
0.25	0.64	0.86	0.87	0.89	0.89	0.91	0.92	0.93	0.93	0.93	0.93	0.93	0.94	0.93	0.93	0.93	0.93	0.93	0.93	0.93	0.94	0.94	0.94	0.94	0.93
0.30	0.64	0.86	0.89	0.89	0.90	0.91	0.92	0.93	0.94	0.93	0.93	0.94	0.94	0.93	0.94	0.93	0.93	0.94	0.94	0.94	0.94	0.93	0.94	0.93	0.94
0.35	0.64	0.86	0.88	0.89	0.90	0.91	0.92	0.93	0.93	0.93	0.94	0.94	0.94	0.94	0.93	0.94	0.93	0.94	0.94	0.93	0.94	0.94	0.94	0.94	0.94
0.40	0.64	0.86	0.87	0.90	0.91	0.92	0.92	0.93	0.93	0.94	0.94	0.93	0.94	0.94	0.94	0.94	0.94	0.94	0.94	0.94	0.94	0.94	0.94	0.94	0.94
0.45	0.64	0.86	0.88	0.90	0.90	0.92	0.93	0.93	0.93	0.93	0.93	0.94	0.94	0.94	0.94	0.94	0.94	0.94	0.94	0.94	0.94	0.94	0.94	0.94	0.93
0.50	0.64	0.87	0.87	0.90	0.90	0.92	0.93	0.93	0.93	0.94	0.94	0.94	0.94	0.94	0.94	0.94	0.94	0.94	0.94	0.94	0.94	0.94	0.94	0.94	0.94
0.55	0.64	0.87	0.88	0.90	0.91	0.92	0.93	0.93	0.94	0.94	0.94	0.94	0.94	0.94	0.94	0.94	0.94	0.94	0.94	0.94	0.94	0.94	0.94	0.94	0.94
0.6	0.64	0.87	0.87	0.90	0.90	0.92	0.93	0.93	0.94	0.94	0.94	0.94	0.94	0.94	0.94	0.94	0.94	0.94	0.94	0.94	0.94	0.94	0.94	0.94	0.94
0.65	0.64	0.87	0.88	0.91	0.90	0.93	0.93	0.93	0.93	0.94	0.94	0.94	0.94	0.94	0.94	0.95	0.94	0.94	0.94	0.94	0.94	0.94	0.94	0.94	0.94
0.70	0.64	0.87	0.87	0.91	0.91	0.92	0.93	0.94	0.93	0.94	0.94	0.94	0.94	0.94	0.94	0.94	0.94	0.94	0.94	0.94	0.94	0.94	0.94	0.94	0.93
0.75	0.64	0.87	0.86	0.91	0.90	0.93	0.93	0.93	0.94	0.94	0.94	0.95	0.94	0.94	0.94	0.94	0.94	0.94	0.94	0.94	0.93	0.94	0.94	0.94	0.93
0.80	0.64	0.87	0.87	0.91	0.90	0.93	0.93	0.94	0.94	0.94	0.94	0.94	0.94	0.94	0.94	0.94	0.94	0.94	0.94	0.94	0.93	0.94	0.94	0.94	0.92
0.85	0.64	0.87	0.86	0.91	0.90	0.93	0.93	0.94	0.94	0.94	0.94	0.94	0.94	0.94	0.94	0.94	0.94	0.94	0.93	0.93	0.93	0.93	0.95	0.91	0.93
0.90	0.64	0.87	0.87	0.91	0.89	0.93	0.93	0.94	0.94	0.94	0.94	0.94	0.94	0.94	0.94	0.94	0.94	0.94	0.93	0.93	0.93	0.94	0.91	0.92	0.94
0.92	0.64	0.86	0.87	0.91	0.89	0.93	0.93	0.94	0.93	0.94	0.94	0.94	0.94	0.94	0.94	0.94	0.94	0.94	0.94	0.93	0.94	0.91	0.92	0.94	0.87
0.94	0.64	0.86	0.87	0.91	0.88	0.93	0.93	0.94	0.94	0.94	0.94	0.95	0.94	0.94	0.94	0.94	0.94	0.94	0.95	0.95	0.91	0.92	0.94	0.87	0.91
0.96	0.64	0.86	0.87	0.91	0.88	0.93	0.94	0.94	0.93	0.94	0.94	0.94	0.94	0.94	0.94	0.94	0.94	0.94	0.94	0.92	0.92	0.94	0.87	0.91	0.80
0.98	0.64	0.86	0.86	0.91	0.88	0.94	0.94	0.94	0.93	0.94	0.94	0.93	0.94	0.94	0.94	0.94	0.93	0.93	0.92	0.93	0.93	0.88	0.91	0.80	0.86

Colour scale: 0, 0.2, 0.4, 0.6, 0.8
