# Supplementary material for: Variance estimation for effective coverage measures: A simulation study
Source: J Glob Health. 2020 Mar 14;10(1):010506. doi: 10.7189/jogh-10-010506 (PMC7101480; doi:10.7189/jogh-10-010506)
Supplement: Online Supplementary Document [file jogh-10-010506-s001.zip › jogh-10-010506-s001/Appendix S1.pdf]

Appendix S1: Effective coverage definition and estimation

Let X be a binary random variable measuring an individual's use of health services, for instance X=1 if a woman sought antenatal care (ANC) at all or at a certain type of facility and X=0 if not. Define Px=Pr(X=1). The probability can be described as the *coverage* of the intervention in question. Let Y denote a binary random variable representing the *readiness* of some specific aspect of the woman's service environment, for example, whether or not a specific type of facility, such as a public hospital, satisfies specific criteria, in which case Y=1 for satisfactory and Y=0 for unsatisfactory.[1] Define Py=Pr(Y=1). In our application, the data for estimating Px and Py come from different sources.

For example, household survey data may be used to estimate *coverage* and health facility assessment data may be used to estimate *readiness* and the.

The objective is to estimate P, the effective coverage, defined in this example as the probability that a randomly selected woman sought ANC at a health facility and that this facility had a satisfactory level of readiness. Since X and Y are both binary and are independent,

$$\mathrm{P} = Pr(XY = 1) = Pr(X = 1, Y = 1) = Pr\,(X = 1) * Pr\,(Y = 1) = Px * Py\,. \qquad (1)$$

Thus, if Px=0.5 and Py=0.5, the effective coverage is P=0.25. This is the probability that a woman's ANC is provided in a facility of a specific type that has a satisfactory level of readiness.

Suppose that there are two types of facilities at which it is possible to seek ANC: public hospitals (*f*=1) and private hospitals (*f*=2). At the national level, Py1=Pr(Y1=1) is the probability that a randomly selected public hospital satisfies the readiness criteria and Py2=Pr(Y2=1) is the similar probability for private hospitals. Px1=Pr(X1=1) is the probability that ANC is sought in a public hospital and Px2=Pr(X2=1) is the corresponding probability for private hospitals. Because the two types of facilities are distinct, and a single visit can only be in one facility of either type, the addition rule in probability theory applies:

$$\mathrm{P} = Pr(XY = 1) = P\,r(X1 * Y1 = 1) + P\,r(X2 * Y2 = 1) \qquad (2)$$

If there are more than two types of facilities, (2) can be expanded appropriately.

Suppose now that a country has two regions, r=1 and r=2 (where here we are back to the setting with one facility type). Following standard procedures for conditional probabilities, the national-level effective coverage will be given by

$$\mathrm{P} = \Pr(XY = 1) = \Pr(XY = 1 \mid r = 1)\Pr(r = 1) + \Pr(XY = 1 \mid r = 2)\Pr(r = 2) \qquad (3)$$

In words, the effective coverage is calculated within each region, and the weighted average of the two regional estimates yields the national estimate. If there are more than two regions, (3) must be expanded appropriately. If there is any variation in Px or Py across regions or facility types, this should be taken into account by using (3), because the estimate of P will almost always be affected.

For example, consider two regions, Region 1 and Region 2, which are of equal size. Suppose that in Region 1, Px=0.25 and Py=0.25, while in Region 2, Px=0.75 and Py=0.75. Using the national data, the

[1] In a parallel derivation shown in Appendix S3, Y is not binary but is a scale that ranges between 0 and 1, for example as the fraction of potential criteria that a facility satisfies.

national coverage would be 0.5 and the national readiness would be 0.5, as above, giving P=0.25. However, using the disaggregated formula (3), $\mathrm{P} = Pr\ (XY = 1)\ = 0.25 * 0.25 * 0.5 +$ 0.75*0.75*0.5=0.3125, which is higher than the unadjusted estimate.

As another possibility, say that in Region 1, Px=0.25 and Py=0.75; in Region 2, Px=0.75 and Py=0.25, and again the two regions are of equal size. The national coverage would be 0.5 and the national readiness would be 0.5, as above, but Region 1 has lower coverage and better readiness; Region 2 has the opposite combination. The two regions have the same effective coverage, 0.25*0.75=0.1875, and the national estimate is $\mathrm{P} = Pr\ (XY = 1)\ = 0.25 * 0.75 * 0.5 + 0.75 * 0.25 * 0.5 = 0.1875$, which is lower than the unadjusted estimate of 0.25.

These two hypothetical examples are sufficient to show that an estimate that takes subnational variation into account is generally different from one that does not. The adjusted estimate can be either higher or lower than the unadjusted estimate.

Similarly, consider variation by facility type. If at the national level the proportion of women who receive 4 standard ANC visits is 0.5, and the probability that a facility is ready to provide this service is 0.5, then the $\mathrm{P} = Pr\ (XY = 1)$ would be erroneously calculated to be 0.25, but if the proportion of women going to the few hospitals is 0.4, where the probability of readiness is actually 0.8 and the proportion of women going to primary facilities is 0.1, where the probability of readiness is 0.4, $\mathrm{P} = Pr\ (XY = 1)\ = 0.4 * 0.8 + 0.1 * 0.4 = 0.36$.
