# Supplementary material for: Variance estimation for effective coverage measures: A simulation study
Source: J Glob Health. 2020 Mar 14;10(1):010506. doi: 10.7189/jogh-10-010506 (PMC7101480; doi:10.7189/jogh-10-010506)
Supplement: Online Supplementary Document [file jogh-10-010506-s001.zip › jogh-10-010506-s001/Appendix S3.pdf]

Appendix S3. Variance calculations with an interval–level measure of service readiness

It was assumed that Y is binary, only taking the values 0 and 1, but a more nuanced measure of readiness in the service environment would be a scale. If this scale is constructed to be in a range from 0 to 1, perhaps as the proportion of a set of criteria that are satisfied by a specific type of facility, then the interpretation and procedures are very similar, because Py is the expected value or population mean of the binary version of Y. Effective coverage would be defined as the expected level of readiness due to the specific type of facility, for a randomly selected woman's last birth. In the formulas, P_y would be replaced by μ_y, the population mean of Y; and p_y would be replaced by $\underline{y}$, the sample mean of Y, etc.

For the direct method, suppose that $\underline{Y}_{rf}$ is the average of n_{yrf} continuous random variables bounded on (0,1) with mean P_{yrf}, and suppose that $\underline{X}_{rf}$ is the average of n_{xrf} independent binary random variables, with prevalence P_{xrf}; in particular, first suppose that $\underline{Y}_{rf}$ is the average of independent random variables from a distribution with mean $\mu_{yrf=}P_{yrf}$ and variance σ^2. Then $\underline{Y}_{rf}$ follows a normal distribution with mean P_{yrf} and $se^2(\underline{Y}_{rf}) = \sigma^2/n_{yrf}$. The expression for $se^2(\underline{X}_{rf}\underline{Y}_{rf})$ is then given by

$$se^2(\underline{X}_{rf}\underline{Y}_{rf}) = [(P_{xrf}(1 - P_{xrf}))/n_{xrf} + P_{xrf}{}^2][\sigma^2/n_{yrf} + P_{yrf}{}^2] - \left(P_{yrf}P_{xrf}\right)^2$$

We can estimate $se^2(\underline{Y}_{rf})$ with $s^2(\underline{Y}_{rf}) = [\frac{1}{n_{yrf}}(\frac{\sum(Y_i-Y)^2}{(n_{yrf}-1)})]$. It follows that $se^2(\underline{X}_{rf}\underline{Y}_{rf})$ can be estimated by

$$s^2(\underline{X}_{rf}\underline{Y}_{rf}) = [(p_{xrf}^2(1 - p_{xrf}^2))/n_{xrf} + p_{xrf}^2]\,[[\frac{1}{n_{yrf}}(\frac{\sum(Y_i - Y)^2}{(n_{yrf} - 1)})] + p_{yrf}^2] - \left(p_{xrf}p_{yrf}\right)^2$$

To estimate the variance at the regional level, we have:

$$s^2(\underline{X}_r\underline{Y}_r) = \sum_f \quad [(p_{xrf}^2(1 - p_{xrf}^2))/n_{xrf} + p_{xrf}^2][[\frac{1}{n_{yrf}}(\frac{\sum(Y_i - Y)^2}{(n_{yrf} - 1)})] + p_{yrf}^2] - \left(p_{xrf}p_{yrf}\right)^2$$

And to estimate the variance at the national level, we have:

$$s^2(\underline{X}\,\underline{Y}) = \sum_r \quad w_r^2 \sum_f \quad [(p_{xrf}^2(1 - p_{xrf}^2))/n_{xrf} + p_{xrf}^2][[\frac{1}{n_{yrf}}(\frac{\sum(Y_i - Y)^2}{(n_{yrf} - 1)})] + p_{yrf}^2] - \left(p_{xrf}p_{yrf}\right)^2$$

If the data arise from a complex survey design, the components needed to compute $s^2(\underline{X}_{rf}\underline{Y}_{rf})$ can be obtained using software that takes into account the sampling design.

In the application of the delta model, the logit model for Y would be replaced by a linear probability model. The only change to the formulas given in Appendix 2 is that $E1 = \frac{\partial p_{1rf}}{\partial b_{1rf}} = 1$. Equations 18-20 in the paper would be replaced by 27-29:

$$\frac{\partial F_{rf}}{\partial b_{1rf}} = \frac{1}{[p_{1rf}(1-p_{rf})]} \quad \text{and} \quad \frac{\partial F_{rf}}{\partial b_{2rf}} = (1-p_{2rf})/(1-p_{rf}) \tag{27}$$

$$\frac{\partial F_r}{\partial b_{1rf}} = p_{2rf}/[p_r(1-p_r)] \quad \text{and} \quad \frac{\partial F_r}{\partial b_{2rf}} = p_{rf}(1-p_{2rf})/[p_r(1-p_r)] \tag{28}$$

$$\frac{\partial F}{\partial b_{1rf}} = w_r p_{2rf}/[p\ \ (1-p\ )] \quad \text{and} \quad \frac{\partial F}{\partial b_{2rf}} = w_r p_{rf}(1-p_{2rf})/[p\ \ (1-p\ )] \tag{29}$$

Note that in the formulas for both the exact and delta methods, independence of the different effective coverage estimates is assumed.
