# Supplementary material for: Variance estimation for effective coverage measures: A simulation study
Source: J Glob Health. 2020 Mar 14;10(1):010506. doi: 10.7189/jogh-10-010506 (PMC7101480; doi:10.7189/jogh-10-010506)
Supplement: Online Supplementary Document [file jogh-10-010506-s001.zip › jogh-10-010506-s001/Appendix S2.pdf]

Appendix S2. Partial derivatives relevant to the chain rule of differentiation for delta method

Define

$A1 = \frac{\partial F_{rf}}{\partial p_{rf}} = 1/[p_{rf}(1 - p_{rf})]$; $A2 = \frac{\partial F_r}{\partial p_r} = 1/[p_r(1 - p_r)]$; $A3 = \frac{\partial F}{\partial p} = 1/[p\,(1 - p\,)]$;

$B = \frac{\partial p}{\partial p_r} = w_r$; $C = \frac{\partial p_r}{\partial p_{rf}}$ =1; $D1 = \frac{\partial p_{rf}}{\partial p_{1rf}} = p_{2rf}$; $D2 = \frac{\partial p_{rf}}{\partial p_{2rf}} = p_{1rf}$; and

$E1 = \frac{\partial p_{1rf}}{\partial b_{1rf}} = p_{1rf}(1 - p_{1rf})$; $E2 = \frac{\partial p_{2rf}}{\partial b_{2rf}} = p_{2rf}(1 - p_{2rf})$.

Then $\frac{\partial F_{rf}}{\partial b_{krf}} = A1 * Dk * Ek$; $\frac{\partial F_r}{\partial b_{krf}} = A2 * C * Dk * Ek$; and $\frac{\partial F}{\partial b_{krf}} = A3 * B * C * Dk * Ek$
