# Supplementary material for: Variance estimation for effective coverage measures: A simulation study
Source: J Glob Health. 2020 Mar 14;10(1):010506. doi: 10.7189/jogh-10-010506 (PMC7101480; doi:10.7189/jogh-10-010506)
Supplement: Online Supplementary Document [file jogh-10-010506-s001.zip › jogh-10-010506-s001/ECvar_v2ProgramDesc.docx]

ec_var

This global program calculates effective coverage point estimates and confidence intervals based on the delta and exact methods, saves individual method results and returns new summary dataset with point estimates and selected confidence intervals. Users can specify stratifying variables that apply to both datasets (e.g., sub-national region) with the ecstrata option and/or population characteristics to create separate estimates for a sub-group with the csubset option. Use of ecstrata is recommended whenever surveys have been sampled by strata such as region to provide more accurate estimation of the overall estimate.

Analyst defines working directory or file path using global drive and provides the input data as specified below. Output is saved as a new dataset; a second dataset with stratum-specific results by facility type is saved for reference, using the delta method only (ECd_`stratum’_`factype’.dta). Written for Stata version 15. Authors: Tom Pullum, Hannah Leslie, Sara Sauer, Lindsay Mallick, Wenjuan Wang. Last update: January 22, 2020.

**ec_var** *methods* , cdata(str) qdata(str) factype(str) qvar(str) [QCONtinuous ecstrata(str) cstrata(str) cclus(str) cwts(str) csubset(str) qstrata(str) qclus(str) qwts(str) cfpc(str) qfpc(str) alpha(real 0.05) saveas(str) show]

Main

methods: one or both of ‘delta’ and ‘exact’, or ‘both’. Can be capitalized.

Options

Required

cdata: name of dataset with coverage data in working directory or $drive folder

qdata: name of dataset with readiness/quality data in working directory or $drive folder

factype: name of variable found in both datasets. In qdata, it is the type of facility. In cdata, it is the facility used for those accessing the service, which can include a level for “None / home / informal.” Values must align between datasets; minimum value should be 1.

qvar: name of variable in qdata indicating the level of readiness per facility. Specify option qcontinuous if interval.

Optional

qcontinuous: indicates readiness/quality variable is interval and not binary. Programs default to binary if not specified.

ecstrata: name of variable to request stratified estimates, for instance by sub-national region, in addition to an overall estimate. Minimum value should be 1 or greater. Recommended for use even for overall estimates when survey data have been collected by strata such as region to improve the overall estimate.

csubset: name of variable to request estimates separated by a categorical trait in the population (coverage) data, for instance wealth quintile or educational attainment. Covariate should be numbered consecutively and non-missing or with missing as a labeled consecutive value. Results require assumption of no systematic difference in health system ‘quality’ based on this trait within strata of health facility type (and region or other sampling strata as specified with ecstrata). Overall estimates should be calculated separately.

Sample design considerations

The programs will use survey settings if the datasets provided have been saved already svyset. Specify the design options below to set sampling design for data that are not already svyset; these options will not override an existing svyset command. The options defined below do not need to match between the two datasets; they are applied independently.

cstrata / qstrata: variable name for sampling strata in coverage data/readiness data respectively to use in svyset (these do not need to match ecstrata).

cclus/ qclus: variable name for primary sampling unit or cluster in coverage data / readiness data respectively to use in svyset.

cwts / qwts: variable name for sampling weights in coverage data/readiness data respectively to use in svyset.

cfpc / qfpc: variable to provide finite sample correction in survey set up for coverage data / readiness data respectively. See help svyset for details.

Output options

alpha: set desired level for confidence interval. Default = 0.05 for 95% CI

saveas: name the dataset created with summary results. Default = ECresults.dta

show: include this option to print the summary results (coverage, effective coverage, and confidence interval for selected estimation method) on Stata interface in addition to saving the results as a dataset.

ec_delta

Specific program to evaluate the point estimate(s) of effective coverage and to use the Delta method to obtain confidence intervals; called by ec_var. Options listed below. Output includes ccoeff.dta (coverage coefficients), qcoeff.dta (readiness coefficients), ectmp2.dta (effective coverage coefficients), and ECd_all.dta (effective coverage coefficients with confidence intervals). At the moment coverage indicators must be binary for correct estimation; readiness indicators can be binary or continuous.

Options (as for ec_var)

Required

cdata: name of dataset with coverage data in working directory or $drive folder

qdata: name of dataset with readiness/quality data in working directory or $drive folder

factype: name of variable found in both datasets. In qdata, it is the type of facility. In cdata, it is the facility used for those accessing the service, which can include a level for “None / home / informal.” Values must align between datasets; minimum value should be 1.

qvar: name of variable in qdata indicating the level of readiness. Specify option qcontinuous if interval.

Optional

qcontinuous: indicates readiness/quality variable is interval and not binary.

ecstrata: name of variable to request stratified estimates, for instance by sub-national region, in addition to an overall estimate. Minimum value should be 1 or greater. Recommended for use even for overall estimates when survey data have been collected by strata such as region to improve the overall estimate.

csubset: name of variable to request estimates separated by a categorical trait in the population (coverage) data, for instance wealth quintile or educational attainment. Covariate should be numbered consecutively and non-missing or with missing as a labeled consecutive value. Results require assumption of no systematic difference in health system ‘quality’ based on this trait within strata of health facility type (and region or other sampling strata as specified with ecstrata). Overall estimates should be calculated separately.

Sample design considerations

The program will use survey settings if the datasets provided have been saved already svyset. Specify the design options below to set sampling design for data that are not already svyset; these options will not override an existing svyset command.

cstrata / qstrata: variable name for sampling strata in coverage data/readiness data respectively

cclus/ qclus: variable name for primary sampling unit or cluster in coverage data / readiness data respectively

cwts / qwts: variable name for sampling weights in coverage data/readiness data respectively

cfpc / qfpc: variable to provide finite sample correction in survey set up for coverage data / readiness data respectively. See help svyset for details.

Output options

alpha: set desired level for confidence interval. Default = 0.05 for 95% CI

ec_exact

Specific program to evaluate the exact method of variance of a product to construct confidence intervals around effective coverage estimates, called by ec_var. Input options defined below. Output is ECe_all.dta. At the moment the program will work only with binary coverage indicators; readiness indicators can be binary or continuous. Must run via ec_var or after running ec_delta; executes based on input data ectmp2.dta produced by ec_delta.

Options (as for ec_var)

Required

factype: name of variable found in both datasets. In qdata, it is the type of facility. In cdata, it is the facility used for those accessing the service, which can include a level for “None / home / informal.” Values must align between datasets; minimum value should be 1.

Optional

qcontinuous: indicates readiness/quality variable is interval and not binary.

ecstrata: name of variable to request stratified estimates, for instance by sub-national region, in addition to an overall estimate

Output options

alpha: set desired level for confidence interval. Default = 0.05 for 95% CI
