# Supplementary material for: Erratum: Variance estimation for effective coverage measures: A simulation study
Source: J Glob Health. 2021 Nov 15;11:01009. doi: 10.7189/jogh.11.01009 (PMC8576354; doi:10.7189/jogh.11.01009)
Supplement: Online Supplementary Document [file jogh-11-01009-s001.zip › Appendix S6 Corrected.pdf]

Appendix S6: Sub-national results, input-adjusted coverage of antenatal care in Senegal

Readiness measure	Region	Adjusted coverage	95% CI, Delta method		95% CI, exact method	
**Coverage measure: 1 ANC visit**						
BP	Dakar	90.7%	70.2%	97.6%	80.4%	101.0%
BP	Ziguinchor	96.3%	37.4%	99.9%	86.0%	106.6%
BP	Diourbel	89.4%	70.7%	96.7%	84.2%	94.6%
BP	Saint-Louis	93.9%	67.8%	99.1%	86.7%	101.0%
BP	Tambacounda	80.5%	51.2%	94.2%	72.3%	88.6%
BP	Kaola	90.1%	77.2%	96.1%	83.9%	96.3%
BP	Thiès	88.4%	66.2%	96.7%	77.9%	98.8%
BP	Louga	83.6%	65.4%	93.2%	74.3%	92.9%
BP	Fatick	89.4%	69.6%	96.9%	79.0%	99.7%
BP	Kolda	80.8%	55.8%	93.4%	72.7%	88.9%
BP	Matam	89.6%	68.4%	97.2%	78.2%	101.0%
BP	Kaffrine	92.6%	72.5%	98.3%	85.9%	99.3%
BP	Kedougou	78.6%	58.8%	90.4%	61.8%	95.4%
BP	Sedhiou	97.8%	15.3%	100.0%	90.9%	104.7%
Diagnostics	Dakar	15.9%	9.7%	24.9%	11.6%	20.1%
Diagnostics	Ziguinchor	0.0%				
Diagnostics	Diourbel	7.8%	4.8%	12.6%	1.9%	13.7%
Diagnostics	Saint-Louis	4.3%	1.9%	9.7%	2.0%	6.7%
Diagnostics	Tambacounda	9.8%	2.9%	28.3%	-5.5%	25.0%
Diagnostics	Kaola	17.8%	9.9%	29.8%	9.0%	26.5%
Diagnostics	Thiès	19.2%	11.4%	30.4%	9.5%	28.9%
Diagnostics	Louga	8.8%	4.0%	18.2%	-2.2%	19.7%
Diagnostics	Fatick	11.1%	4.8%	23.3%	-2.4%	24.5%
Diagnostics	Kolda	18.2%	8.6%	34.6%	13.2%	23.2%
Diagnostics	Matam	7.4%	3.3%	15.9%	-4.3%	19.2%
Diagnostics	Kaffrine	7.6%	4.6%	12.3%	-5.5%	20.7%
Diagnostics	Kedougou	0.0%				
Diagnostics	Sedhiou	1.5%	0.6%	3.6%	-0.3%	3.3%
Sum score	Dakar	66.8%	57.3%	75.1%	61.7%	71.9%
Sum score	Ziguinchor	73.6%	61.6%	82.9%	65.9%	81.3%
Sum score	Diourbel	57.1%	49.1%	64.7%	53.9%	60.3%
Sum score	Saint-Louis	67.0%	57.8%	75.0%	61.6%	72.4%
Sum score	Tambacounda	62.0%	44.3%	77.0%	54.6%	69.4%
Sum score	Kaola	62.0%	54.7%	68.7%	57.9%	66.0%
Sum score	Thiès	64.3%	52.9%	74.2%	59.3%	69.2%
Sum score	Louga	59.5%	50.1%	68.4%	53.8%	65.3%
Sum score	Fatick	61.6%	52.8%	69.7%	55.2%	68.0%
Sum score	Kolda	60.5%	45.3%	74.0%	53.3%	67.8%

Sum score	Matam	63.6%	54.4%	71.9%	56.8%	70.4%
Sum score	Kaffrine	69.1%	59.5%	77.4%	64.4%	73.8%
Sum score	Kedougou	59.5%	46.7%	71.1%	50.4%	68.5%
Sum score	Sedhiou	79.1%	67.9%	87.2%	74.4%	83.8%
**Coverage measure: 4 ANC visits**						
BP	Dakar	65.5%	54.9%	74.8%	56.6%	74.4%
BP	Ziguinchor	54.3%	45.0%	63.4%	44.7%	64.0%
BP	Diourbel	50.2%	41.9%	58.4%	45.3%	55.0%
BP	Saint-Louis	59.0%	47.2%	69.8%	51.7%	66.3%
BP	Tambacounda	33.2%	24.2%	43.7%	26.9%	39.6%
BP	Kaola	53.4%	44.7%	61.9%	47.0%	59.8%
BP	Thiès	53.5%	45.7%	61.1%	46.9%	60.0%
BP	Louga	41.6%	32.7%	51.1%	34.7%	48.5%
BP	Fatick	42.1%	33.5%	51.2%	34.5%	49.7%
BP	Kolda	37.2%	28.7%	46.5%	30.3%	44.1%
BP	Matam	46.4%	37.2%	55.8%	37.3%	55.4%
BP	Kaffrine	45.0%	38.2%	52.0%	38.1%	51.8%
BP	Kedougou	43.2%	32.9%	54.2%	28.7%	57.7%
BP	Sedhiou	47.6%	36.5%	59.0%	39.4%	55.9%
Diagnostics	Dakar	13.3%	8.0%	21.3%	9.7%	16.8%
Diagnostics	Ziguinchor	0.0%				
Diagnostics	Diourbel	5.5%	3.1%	9.5%	1.8%	9.2%
Diagnostics	Saint-Louis	3.4%	1.4%	8.3%	1.3%	5.5%
Diagnostics	Tambacounda	5.4%	2.0%	13.7%	-1.7%	12.5%
Diagnostics	Kaola	11.8%	6.8%	19.8%	6.3%	17.4%
Diagnostics	Thiès	11.4%	7.5%	16.8%	6.3%	16.4%
Diagnostics	Louga	5.6%	2.5%	12.3%	-1.5%	12.8%
Diagnostics	Fatick	5.5%	2.4%	12.1%	-0.9%	11.8%
Diagnostics	Kolda	8.5%	4.3%	16.3%	4.9%	12.2%
Diagnostics	Matam	4.5%	1.9%	10.3%	-2.6%	11.6%
Diagnostics	Kaffrine	3.8%	2.4%	5.9%	-2.8%	10.4%
Diagnostics	Kedougou	0.0%				
Diagnostics	Sedhiou	0.9%	0.3%	2.6%	-0.5%	2.2%
Sum score	Dakar	48.5%	41.0%	56.0%	44.2%	52.8%
Sum score	Ziguinchor	41.5%	34.5%	48.9%	34.5%	48.5%
Sum score	Diourbel	31.8%	26.7%	37.4%	29.2%	34.5%
Sum score	Saint-Louis	42.3%	34.1%	50.8%	37.1%	47.4%
Sum score	Tambacounda	26.4%	19.4%	34.7%	21.3%	31.4%
Sum score	Kaola	37.0%	30.8%	43.5%	33.1%	40.8%
Sum score	Thiès	38.3%	32.7%	44.3%	34.5%	42.2%
Sum score	Louga	29.9%	23.7%	36.9%	25.3%	34.5%
Sum score	Fatick	29.1%	23.4%	35.7%	24.3%	33.9%

Sum score	Kolda	27.8%	21.4%	35.4%	22.3%	33.3%
Sum score	Matam	33.1%	26.8%	40.0%	27.2%	39.0%
Sum score	Kaffrine	33.6%	28.4%	39.2%	29.0%	38.2%
Sum score	Kedougou	33.0%	25.3%	41.8%	25.3%	40.7%
Sum score	Sedhiou	38.5%	29.7%	48.0%	32.4%	44.6%
**Coverage measure: 8 ANC visits**						
BP	Dakar	0.9%	0.3%	3.0%	0.2%	1.6%
BP	Ziguinchor	0.2%	0.0%	1.6%	-0.5%	0.9%
BP	Diourbel	0.3%	0.0%	1.9%	-0.1%	0.7%
BP	Saint-Louis	0.0%				
BP	Tambacounda	0.0%				
BP	Kaola	0.4%	0.1%	2.9%	-0.3%	1.2%
BP	Thiès	0.2%	0.0%	1.7%	-0.2%	0.6%
BP	Louga	0.2%	0.0%	1.3%	-0.3%	0.7%
BP	Fatick	0.0%				
BP	Kolda	0.7%	0.2%	3.0%	-0.4%	1.9%
BP	Matam	0.2%	0.0%	1.4%	-0.5%	0.9%
BP	Kaffrine	0.0%				
BP	Kedougou	0.0%				
BP	Sedhiou	0.0%				
Diagnostics	Dakar	0.4%	0.1%	2.2%	0.0%	0.8%
Diagnostics	Ziguinchor	0.0%				
Diagnostics	Diourbel	0.3%	0.0%	1.9%	-0.1%	0.7%
Diagnostics	Saint-Louis	0.0%				
Diagnostics	Tambacounda	0.0%				
Diagnostics	Kaola	0.0%				
Diagnostics	Thiès	0.1%	0.0%	0.7%	-0.1%	0.3%
Diagnostics	Louga	0.1%	0.0%	0.8%	-0.2%	0.4%
Diagnostics	Fatick	0.0%				
Diagnostics	Kolda	0.3%	0.0%	2.3%	-0.4%	1.1%
Diagnostics	Matam	0.2%	0.0%	1.4%	-0.5%	0.9%
Diagnostics	Kaffrine	0.0%				
Diagnostics	Kedougou	0.0%				
Diagnostics	Sedhiou	0.0%				
Sum score	Dakar	0.7%	0.2%	2.3%	0.2%	1.2%
Sum score	Ziguinchor	0.2%	0.0%	1.2%	-0.4%	0.7%
Sum score	Diourbel	0.2%	0.0%	1.2%		
Sum score	Saint-Louis	0.0%				
Sum score	Tambacounda	0.0%				
Sum score	Kaola	0.4%	0.1%	2.4%		
Sum score	Thiès	0.2%	0.0%	1.1%	-0.1%	0.4%
Sum score	Louga	0.2%	0.0%	1.1%	-0.2%	0.6%

Sum score	Fatick	0.0%				
Sum score	Kolda	0.6%	0.1%	2.3%	-0.3%	1.4%
Sum score	Matam	0.2%	0.0%	1.2%		
Sum score	Kaffrine	0.0%				
Sum score	Kedougou	0.0%				
Sum score	Sedhiou	0.0%				

Yellow highlights indicate regions where variance could not be calculated due to input-adjusted coverage equal to 0% or 100% or standard deviation of 0; pink highlights show invalid confidence intervals.
