# Supplementary figures and images for: Erratum: Variance estimation for effective coverage measures: A simulation study
Source: J Glob Health. 2021 Nov 15;11:01009. doi: 10.7189/jogh.11.01009 (PMC8576354; doi:10.7189/jogh.11.01009)

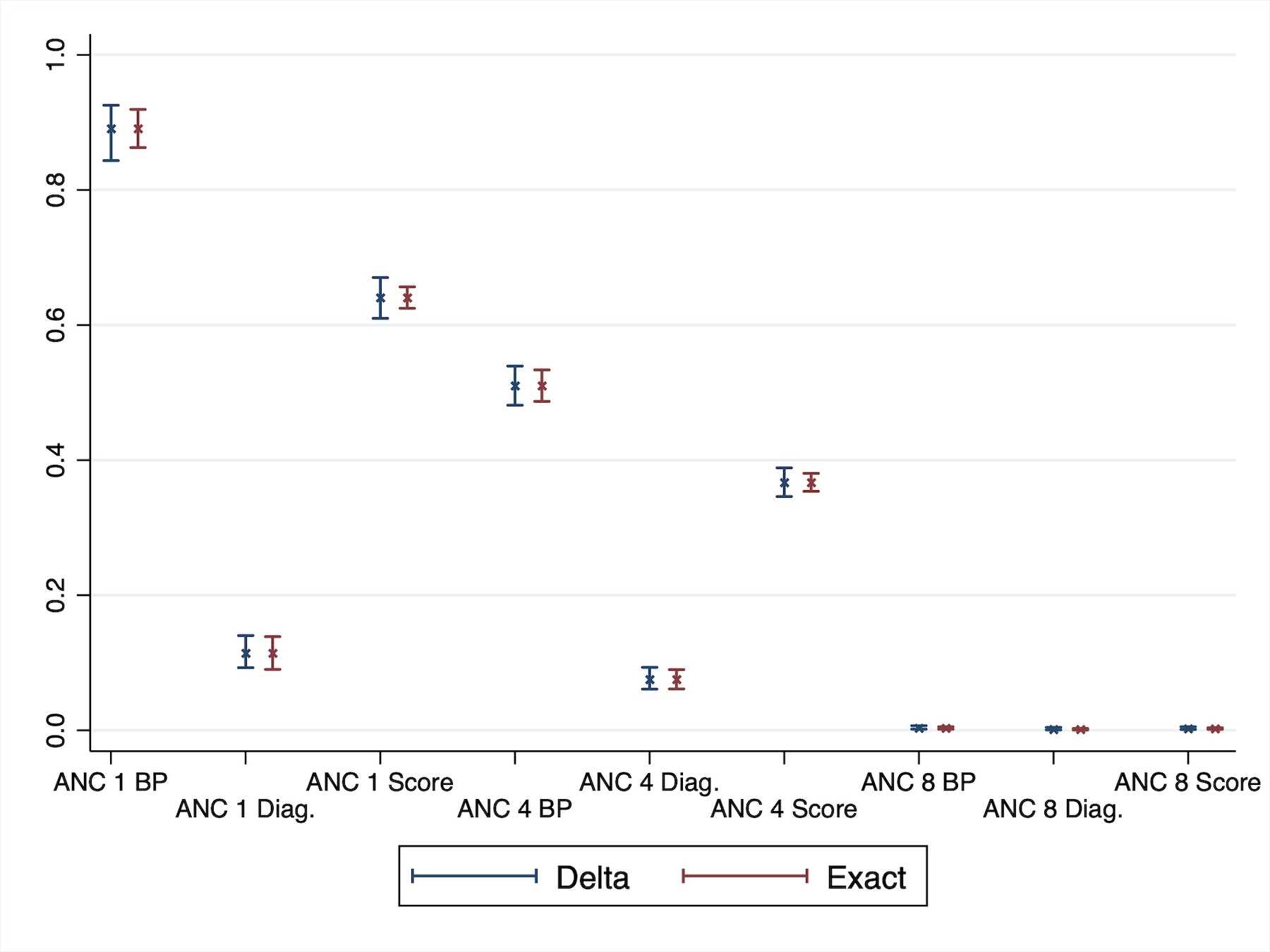

Supplement: Online Supplementary Document [file jogh-11-01009-s001.zip › Fig5_cor.jpg]
